# Establishment of atropisomerism in 3-indolyl furanoids: a synthetic, experimental and theoretical perspective†

**DOI:** 10.1039/c9ra05350f

**Published:** 2019-07-18

**Authors:** Sourav Chatterjee, Pinaki Bhattacharjee, Glenn L. Butterfoss, Anushree Achari, Parasuraman Jaisankar

**Affiliations:** Laboratory of Catalysis and Chemical Biology, Department of Organic and Medicinal Chemistry, CSIR-Indian Institute of Chemical Biology 4 Raja S. C. Mullick Road Kolkata-700 032 India jaisankar@iicb.res.in; Center for Genomics and Systems Biology, New York University Abu Dhabi Abu Dhabi-129188 United Arab Emirates

## Abstract

Introduction of axial chirality in bioactive 3-indolyl furanoids has been achieved by systematic alteration of functional groups around the stereogenic axis, keeping in mind that atropisomerically pure analogues may possess different binding affinities and selectivities towards a target protein. The kinetics of racemization of axially chiral 3-indolyl furanoids have been studied through chiral HPLC analysis, electronic circular dichroism (ECD) spectroscopy, and computational modeling. The results identify the configurational parameters for optically pure 3-indolyl furanoids to exist as stable and isolable atropisomeric form.

## Abstract 2

A number of axially chiral 3-indolyl furanoids have been synthesized and the individual enantiomers are found to be configurationally stable and isolable at room temperature.

## Graphical abstract

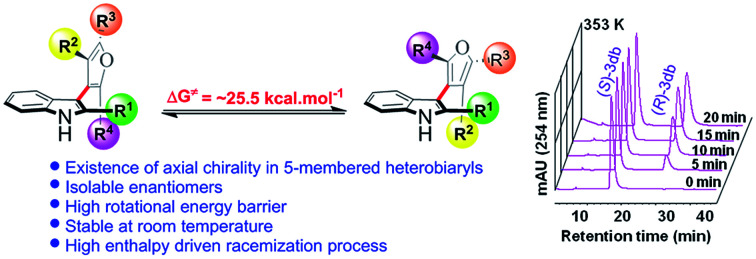

## Introduction

**Fig. 1:**
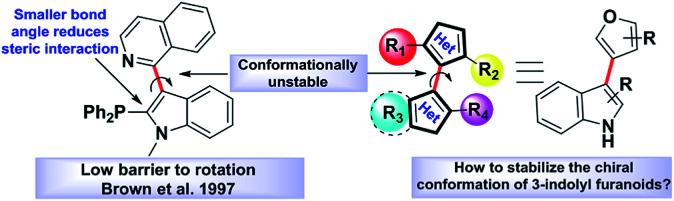
Axial chirality in indole-based heterobiaryls.

Atropisomerism is related to single bonds that join a pair of hindered planar groups leading to axial chirality within a molecule.^1^ Moreover, in contrast to traditional sp^3^ carbon-centred chirality, control of axial chirality and atropisomer selectivity remains highly appealing, especially in the field of complex drug like molecules synthesis, chiral ligand designing and natural product synthesis.^2^ Recent literature precedence suggests that atropisomerically stable enantiomers of the bioactive lead molecules show strikingly differential affinities and selectivity for a specific target protein.^3^ One of the key criteria to this approach is the incorporation of an appropriate substitution onto a bioactive lead molecule in such a fashion that will render the conformationally stable atropisomers. Investigations towards the potential biological applications of 3-indolyl furanoids revealed that they have potential anti-ulcer activity by inhibiting MMP-9,^4*a*^ anti-proliferative activity by inhibiting mitochondrial complex III^4*b*^ and biofilm disruption activities in *Pseudomonas aeruginosa*.^4*b*^ Therefore, we were curious to know whether induction of axial chirality of 3-indolyl furanoids would make one enantiomer have an enhanced biological activity than the other towards a specific target. However, unlike the synthesis of six member atropisomeric heterocycles, five membered atropisomeric heterocycles are not commonly explored and still constitute an significant challenge in the arena of synthetic organic chemistry.^5^ The reduced barrier to rotation resulted due to the modified bond angles of the five membered ring system where the *ortho*-substituents are not held as closely in space to the adjacent aromatic group furnishing lower conformational stability and loss of chirality.^5^ It is well known that changing the dihedral and bite angles of biaryls can drastically affect the conformational stability of a single atropisomer (Fig. 1).^5^

**Fig. 2:**
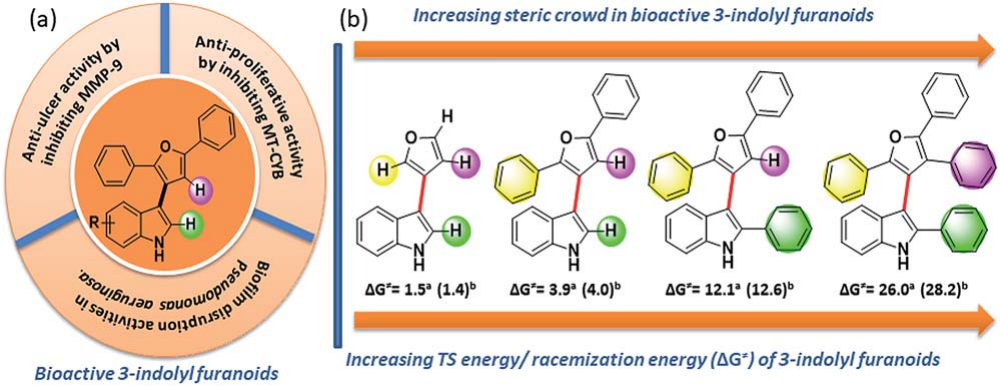
(a) Bioactivity of 3-indolyl furanoids. (b) Sequential increase of steric demand along its stereogenic axis towards conformational stability of bioactive 3-indolyl furanoids.

To design molecules with high energy rotational barrier to separate the two enantiomers and to be able to perform chemical reactions with the enantiomerically pure atropisomers, it is necessary to study the inversion mechanism and the influence of substituents on the di-*ortho*-substituted biaryls in detail. Dynamic HPLC, CD spectroscopy, GC, SFC, electrokinetic chromatography, and electrophoresis have frequently been exploited to explore the potential of these techniques for determining racemization energy barriers of axially chiral molecules.^6^ Several research groups extensively studied racemization kinetics of a wide variety of atropisomers.^7^ However, most of the optically pure separations were found not to be configurationally stable at room temperature because of their very low activation barrier to racemization (Fig. 2).^5*c*^

A true application of enantiopure samples can only be considered if they could be isolated from the racemic mixture and be stable for an extended period of time at room temperature; more precisely, they need to possess significantly higher activation barrier to racemization along the chiral axes. Indeed, though there are a few reports on the atropisomerism chemistry of pyrrole and carbazole containing molecules,^8^ indole atropisomers are often found to be configurationally unstable.^9^

While facing the challenges of inducing axial chirality in five membered heterocycles, our earlier reports successfully addressed the synthesis of fully substituted atropisomeric 3,3′-bipyrroles.^8*e*,*f*^ In continuation of our search for other atropisomeric heterobiaryls, we wanted to investigate if indole-based heterobiaryls like 3-indolyl furanoids could possess axial chirality, and if so, what might be the minimum obligatory condition to induce axial chirality.

## Results and discussion

In order to understand the minimum obligatory condition to obtain axial chirality we employed DFT calculations, which confirmed the importance of steric bulk at all positions adjacent to the stereogenic bond. For example, unsubstituted 3-indolyl furanoid is predicted to have potential energy barrier of 1.5 and 1.4 kcal mol^−1^ and the values for variant of 3da missing the furan C5 phenyl group are predicted to be 12.1 and 12.6 kcal mol^−1^, well below the threshold for atropisomerism. Moreover, DFT models predict a lower barrier for inversion in 3bb than 3db. The methylated indole (3bb), while providing some steric bulk, does not sufficiently strain the transition state.^10^

Driven by our long-standing interest on the development of atropisomeric five membered heterobiaryls and, we first synthesized 3-(2,5-diphenylfuran-3-yl)-1*H*-indole (3aa, Table 1) and in agreement with the DFT calculations, no axial chirality was found along the indole C3-furan C3 axis. Our further attempt was to study 3-(2,5-diphenylfuran-3-yl)-2-phenyl-1*H*-indole (3da, Table 1). But as expected, the presence of steric bulk at indole C2 was insufficient to induce axial chirality as 3da could not be separated on chiral HPLC. These studies demonstrated that an absence of steric bulk at furan C4 in both 3aa and 3da might be responsible for the configurational instability of the products. Similarly, 3ba and 3ca did not show any axial chirality.

**Table 1:** Substrate scope for acid catalyzed synthesis of 3-indolyl furanoids^a^

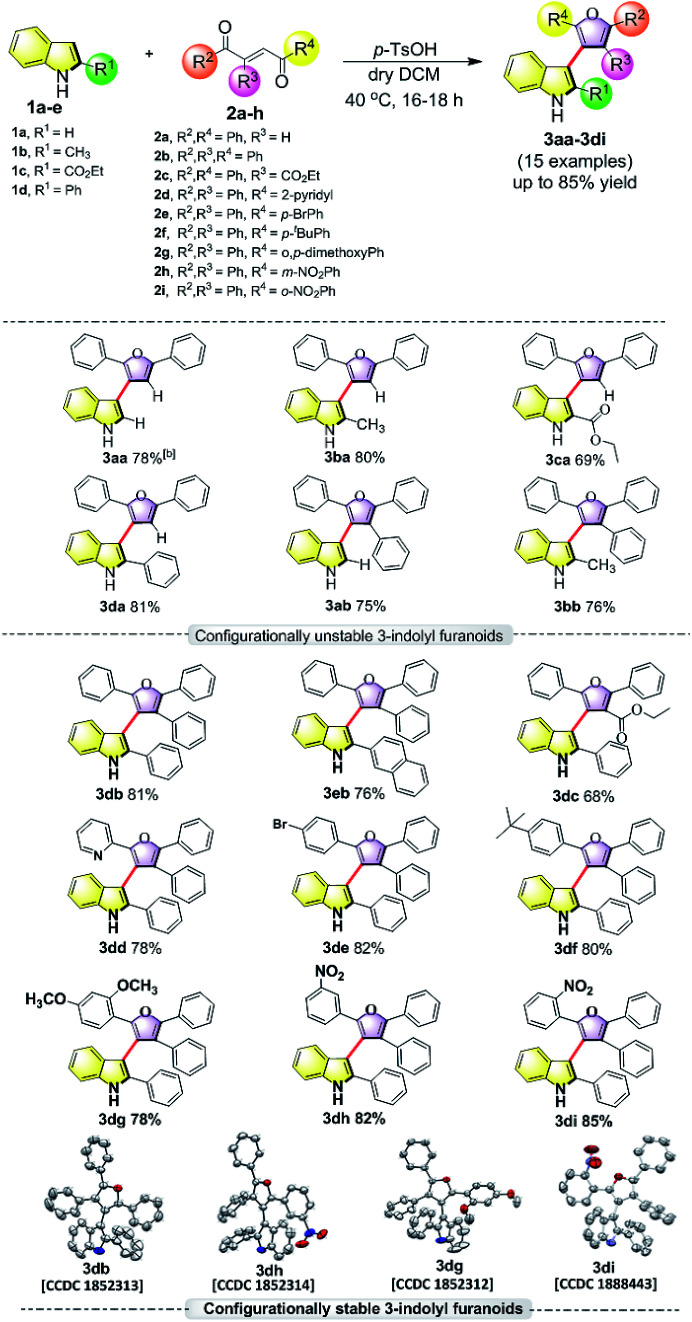

a Reaction conditions: indole (1.0 equiv.), enedione (1.1 equiv.), *p*-TsOH (0.5 equiv.), dry DCM (5.0 mL), 40 °C, 16–18 h.

b Isolated yield by flash chromatography.

Thus, one can ask whether only the substitution at furan C2 and C4 can alone induce axial chirality in the products. In order to ensure this, we studied 3-(2,4,5-triphenylfuran-3-yl)-1*H*-indole (3ab), where in indole C2 is lacking steric demand along its stereogenic axes, as well as 3bb to establish atropisomerism within the system. Interestingly, HPLC elution profile of 3bb was not well resolved but showed a minor plateau between the enantiomer peaks. The similar observation identified for 3dc.^10^ These findings demonstrate that only a complete steric constraint along the stereogenic axes of 3-indolyl furanoids might impart their stereochemical stability. Therefore, we next focused on enantioseparation of fully substituted 3-indolyl furanoids, 2-phenyl-3-(2,4,5-triphenylfuran-3-yl)-1*H*-indole (3db, Table 1). Gratifyingly, the enantiomers of 3db could be separated well on a chiral HPLC time scale at room temperature (300 K). The reactions proceeded well with different substituents addressing structural diversity for both indoles and enediones (Table 1) and a number of atropisomeric 3-indolyl furanoids (3db to 3di, Table 1) were successfully synthesized in good yields (68 to 85%).^10^ In order to understand the mode of action of these bioactive 3-indolyl-furanoids it is essential to have a complete knowledge of the racemization barriers between the enantiomers and other chiroptical properties of atropisomeric 3-indolyl furanoids. Therefore, a fully phenyl substituted atropisomeric 3-indolyl furanoid 3db was considered as an exemplar to understand stereogenic C–C bond rotation dynamics. Accordingly, the 3-indolyl furanoid 3db was separated by HPLC and the optical rotation of the individual enantiomers of 3db was found to be positive (+) for the 1st eluted enantiomer and negative (−) for the 2nd eluted enantiomer.

The CD spectra of the separated enantiomers confirm negative CD signal for (+) 3db with HPLC chromatogram retention time 17.127 min and positive CD signal for (−) 3db with retention time 34.382 min (Fig. 4). The absolute configuration of the two enantiomers of 3db follows from the theoretical CD spectra calculations.

**Fig. 3:**
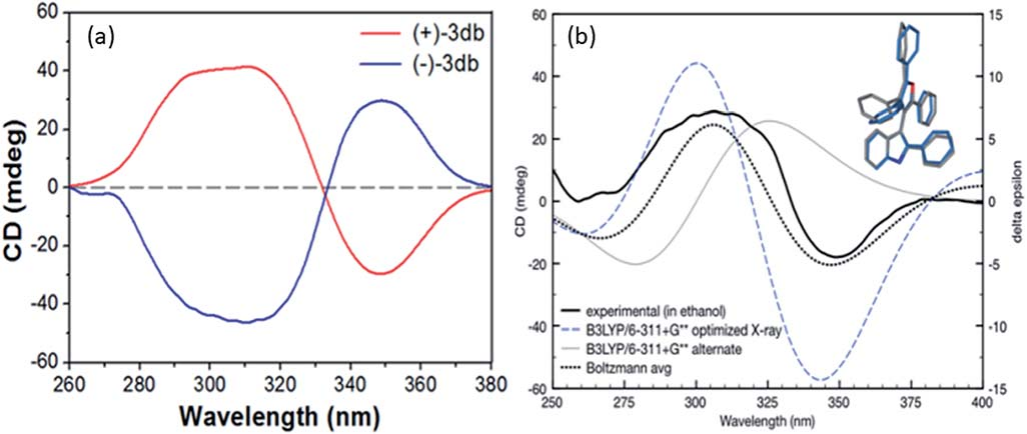
(a) CD spectra of the two enantiomers of 3db. (b) Comparison of experimental CD spectrum (in mdeg) of (+)-3db with theoretical predictions (in delta epsilon). Structure and curve in grey represent the lowest energy conformation observed B3LYP/6-311+G** in implicit ethanol, which coincides with the (*S*)-configuration of one of two crystal conformations. Structure and curve in blue represent the second lowest energy (*S*)-configuration observed. The Boltzmann weighted CD prediction (black dotted curve) of two lowest energy conformations (grey and blue) is also shown.

The predicted spectra of the (*S*)-configuration of the most favorable 3db geometry identified reasonably matched the experimental CD curve (blue line, Fig. 3, these optimizations and spectra are at the B3LYP/6-311+G** level of theory).

**Fig. 4:**
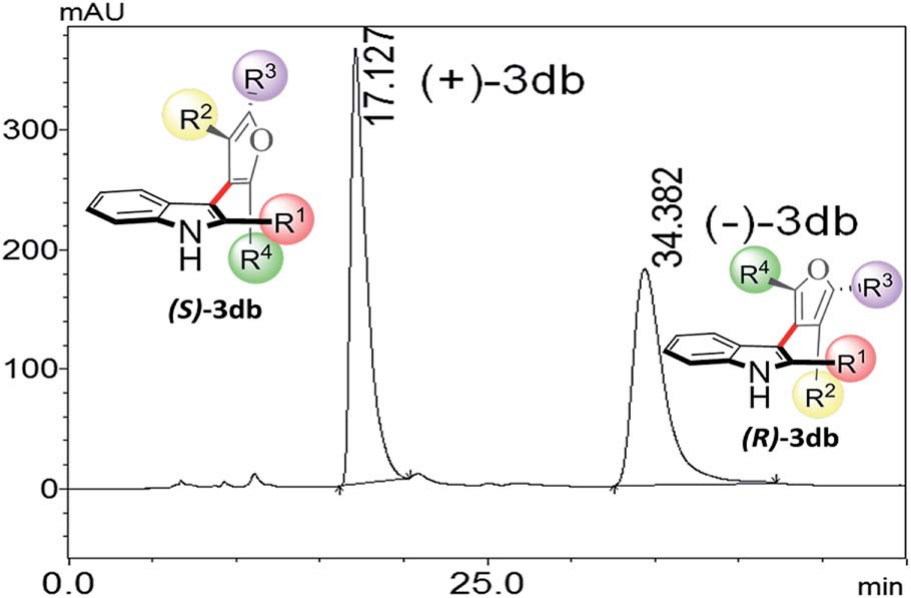
Chiral HPLC profile of 3db.

Fit to the experimental CD of (+)-3db was further improved when we added a Boltzmann weighted contribution of the second lowest energy geometry identified at this level of theory (which had a 60° rotation of the furan C4 phenyl ring, combined prediction is the black dotted line in Fig. 3). Therefore, the absolute configurations of (+)-3db and (−)-3db are confirmed to be *S* and *R*, respectively.

Having two pure atropisomers of 3db in hand, off-column kinetic experiment was performed by chiral HPLC analysis. Gradual decrease of ee of (*S*)-3db was observed with time. Complete racemization was observed after 20 min and 13 h at 353 K and 333 K respectively.^10^ A plot of ee *vs.* time at 353 K and 333 K confirms a first order exponential decay giving a first order rate constant (*k*_rac_) 1.62 × 10^−3^ s^−1^ and 1.44 × 10^−4^ s^−1^ respectively. The activation barrier of racemization (Δ*G*^≠^) as calculated from *k*_enant_*via* the Eyring equation is 25.75 kcal mol^−1^ at 353 K and 25.86 kcal mol^−1^ at 333 K respectively. After determining a high Δ*G*^≠^ for 3db, we measured the stability of enantiopure (*S*)-3db at room temperature (300 K) and obviously, racemization kinetics showed a slow exponential decay of enantiomeric excess with time, resulting in complete racemization after 23 days at 300 K.^10^ The Δ*G*^≠^, determined at room temperature (300 K, 26.29 kcal mol^−1^) was also in agreement with values determined at 353 K and 333 K.^10^

To support HPLC results, isolated enantiopure (*S*)-3db (1.03 mM in EtOH) was subjected to dynamic ECD; at various temperatures. Complete racemization was achieved after 30 min at 353 K and 10 h at 333 K, which were well in accordance with the exponential decay of ee recorded by chiral HPLC.^10^ Finally, other thermodynamical parameters, such as activation enthalpy (Δ*H*^≠^) and activation entropy (Δ*S*^≠^) of the isomerization of atropisomer 3db were determined *via* the Eyring equation^10^ and were found to be 28.86 kcal mol^−1^ and 8.12 cal mol^−1^ K^−1^ for enthalpy (Δ*H*^≠^) and activation entropy (Δ*S*^≠^) respectively.^10^ A large activation enthalpy suggests this process of racemization highly enthalpy driven. A positive value for entropy of activation indicates that the transition state is highly disordered compared to the ground state. Translational, rotational and vibrational degrees of freedom are liberated on going from the ground state to the transition state.

**Fig. 5:**
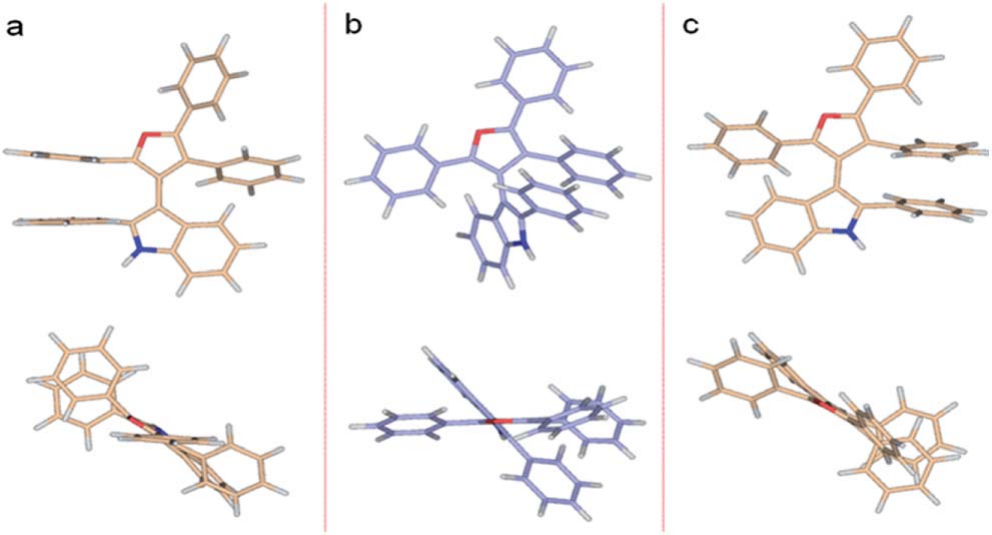
DFT (M062X/6-311G**) optimized structures of 3db. (a) Higher energy TS (Δ*G*^≠^_rac_ = 28.2 kcal mol^−1^), (b) ground state, (c) lower energy TS (Δ*G*^≠^_rac_ = 26.0 kcal mol^−1^).

DFT predictions of the 3db rotational energy barrier agree well with the experimental results. The two Δ*G*^≠^ barriers at the M062X/6-311G**//M062X/6-311G** level of theory (Fig. 5) are 26.0 and 28.2 kcal mol^−1^ (298.15 K). The corresponding Δ*H*^≠^ barriers are 24.3 and 26.3 kcal mol^−1^, respectively, while the potential energies are 25.5 and 27.2 kcal mol^−1^. Barrier heights did not change appreciably when molded in implicit water or ethanol solvents. The lower energy transition state occurs as the furan C4 phenyl passes the indole phenyl group. This successful analysis of axial chirality and racemization kinetics study of 3-indolyl furanoids, examines various steric contributions within the system.

The influence of electron-donating as well as electron-withdrawing groups at C2-phenyl rings of furan towards electronic effect was studied. Significant changes in Δ*G*^≠^ value was observed by changing the substitution at C2 phenyl ring of the furan. In case of 3di, the presence of electron withdrawing nitro group at *ortho* position in C2 phenyl ring of furan has lower Δ*G*^≠^ value (Δ*G*^≠^ = 23.54 kcal mol^−1^) compared to the one that of presence of electron donating methoxy group at *ortho*/*para* position in C2 phenyl ring of furan in 3dg (Δ*G*^≠^ = 24.22 kcal mol^−1^). In case of 3dh, the meta nitro substituent present in C2 phenyl ring of furan showed a decreased Δ*G*^≠^ value (Δ*G*^≠^ = 25.49 kcal mol^−1^) compared to that of 3db wherein C2 phenyl ring of furan is unsubstituted.^10^

**Fig. 6:**
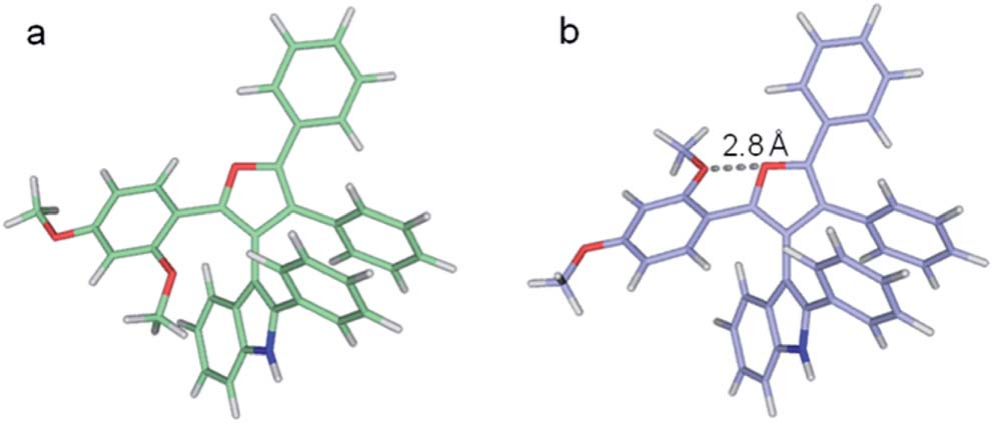
Two optimized structures of 3dg. (a) Lowest energy structure, (b) higher energy local minimum featuring the furan and *ortho*-methoxy oxygens in closer proximity.

At the M062X/6-311G** level of theory, the Δ*G*^≠^ barriers for 3dg, 3dh, and 3di are predicted to be 25.2, 25.3, and 23.4 kcal mol^−1^, respectively. These agree with the experimental inversion rates of 3dh and 3di but are higher than expected for 3dg. On inspection, the 3dg furan C2 methoxy phenyl group can assume various rotational states. In the lowest energy state modeled, the *ortho*-methoxy points towards the indole.^10^ The flipped state puts the *ortho*-methoxy adjacent to the furan oxygen and is predicted to be 3.4 kcal mol^−1^ higher in free energy (Fig. 6). However, the M062X functional accounts for dispersion forces and may overly favor the first state due to contacts between the methoxy and the indole, leading to a larger inversion barrier. Indeed, the second conformation is only 1.0 kcal mol^−1^ more favorable at the B3LYP/6-311G** level of theory (which does not account for dispersion). Notably, in the 3dg crystal structure, the *ortho*-methoxy is in the higher energy orientation.^10^

## Conclusions

In summary, we successfully synthesized and separated the enantiomers of nine axially chiral 3-indolyl furanoids. Individual enantiomers are isolable at room temperature. Racemization kinetics studied by off-column chiral HPLC and electronic circular dichroism (ECD) spectroscopy showed significantly higher activation barrier to rotation (∼25.5 kcal mol^−1^) of 3-indolyl furanoids and the configurational stability for extended periods at room temperature. Electron donating or withdrawing groups may play an important role in the racemization kinetics. The presence of methoxy and nitro group in phenyl ring reduces the energy barrier. Computational modeling further validated the high activation barrier of 3-indolyl furanoids. Our development of these stable atropisomers for biological applications is ongoing and the results will be communicated in due course.

## Conflicts of interest

There are no conflicts to declare.

## Supplementary Material

RA-009-C9RA05350F-s001

RA-009-C9RA05350F-s002
