# Supplementary material for: Establishment of atropisomerism in 3-indolyl furanoids: a synthetic, experimental and theoretical perspective
Source: RSC Adv. 2019 Jul 18;9(39):22384–8. doi: 10.1039/c9ra05350f (PMC9066644; doi:10.1039/c9ra05350f)
Supplement: RA-009-C9RA05350F-s001 [file RA-009-C9RA05350F-s001.pdf]

Electronic Supplementary Material (ESI) for RSC Advances.
This journal is © The Royal Society of Chemistry 2019

Supporting Information

Establishment of atropisomerism in 3-indolyl furanoids: A synthetic , experimental and theoretical perspective

Sourav Chatterjee, [a], ‡ Pinaki Bhattacharjee, [a], ‡ Glenn L.Butterfoss, [b] Anushree Achari, [a] and Parasuraman Jaisankar*[a]

[a]Laboratory of Catalysis and Chemical Biology, Department of Organic and Medicinal Chemistry, CSIR-Indian Institute of Chemical Biology, 4 Raja S. C. Mullick Road, Kolkata - 700 032, India.
[b]Center for Genomics and Systems Biology, New York University, Abu Dhabi, Abu Dhabi-129188, United Arab Emirates.

‡ These authors contributed equally.

** Corresponding authors: Parasuraman Jaisankar; E-mail: jaisankar@iicb.res.in*

Table of contents

Section A: General Information	S02
Section B: Synthetic procedures and spectroscopic data	S02-S08
Section C: ^{1}H NMR, ^{13}C NMR Spectra, and HPLC profiles	S08-S34
Section D: HPLC chromatograms of 3db, 3dg, 3dh and 3di	S35-S63
Section E: Determination of kinetic and thermodynamical parameters	S64-S65
Section F: Temperature dependent ECD analysis	S65-S67
Section G: Verification of kinetic and thermodynamical parameters	S67-S68
Section H: Determination of Enthalpy and Entropy	S69
Section I: X-ray crystal structure determination for **3db**, **3dg**, **3dh**, and **3di**	S70-S78
Section J: Computational results	S78-S89
Section K: References	S90

Section A: General Information

Unless otherwise stated, all reagents were purchased from commercial suppliers and used without further purification. Melting points were determined in open glass capillaries and are uncorrected. All reactions were carried out with Standrad Schlenk techniques under predried nitrogen. ^{1}H NMR spectra were recorded on a Bruker DPX 300 MHz, JEOL JNM-ECZ 400 MHz, and Bruker DRX 600 MHz NMR instrument at ambient temperature either in $CDCl_3$, CD_3OD or DMSO-d_6. ^{13}C NMR spectra were recorded at 75 MHz, 100 MHz, and 150 MHz at ambient temperature. The chemical shifts were recorded in parts per million (ppm) with TMS as internal reference. ^{1}H NMR is reported as follows: Chemical shift, multiplicity (s=singlet, d=doublet, dd=doublet of doublet, brs=broad singlet, t=triplet, q=quartet, m=multiplet), coupling constant and integration. Coupling constants values are given in Hz. All ^{13}C NMR spectra were recorded with complete proton decoupling. Analytical thin-layer chromatography (TLC) was carried out on Merck 20 × 20 cm silica gel 60-F_{254} plates. Column chromatography was done with Biotage flash, silica gel 100-200 mesh. Mass spectral data are corresponds to EIMS or ESIMS and are given in m/z unit. HRMS (m/z) were recorded on a Q-TOF MicroTM mass spectrometer (ESI mode), Waters Xevo G2-XS QTof (ESI mode) and JEOL-JMS 700 (EI mode). Optical rotations were measured on a Perkin Elmer Model-341 polarimeter. Circular Dichroism spectra were recorded on a JASCO J815 unit (Jasco International Co.) equipped with a temperature controller and thermal programmer model PFD 425L/15 in strain free quartz cuvette (55.0 × 10.0 × 2.0 mm internal dimension) having PTFE stopper in "ground type" joint. HPLC analyses were performed on a Shimadzu SPD-M10AVP using Daicel Chiral column. Yield reported was the isolated yield by flash chromatography.

Section B : Synthetic procedures and spectroscopic data

Synthesis of enediones (2b-2i)

(*E*)-1,2,4-triphenylbut-2-ene-1,4-dione (2b). To a stirred solution of benzil (1.0g, 4.75 mmol) and acetophenone (0.55 mL, 4.75 mmol) in methanol (50 mL), was added aq NaOH (209 mg, 5.225 mmol in 10 mL water) dropwise under cold condition. The solution was allowed to stir for 36 h at room temperature. After completion of reaction, methanol was evaporated from the reaction mass, added crushed ice and neutralized with drop wise addition of conc. HCl. A yellowish white precipitate appeared which was filtered through sintered funnel, washed with cold water, and dried. The solid mass was further crystallized in hot methanol to afford 785 mg (53 %) of (*E*)-1,2,4-triphenylbut-2-ene-1,4-dione (**2b)** as pale yellow solid. Analytical data was matched with the literature report.[1]

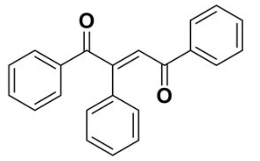

2b

Pale yellow solid; Yield: 53% 785 mg. m.p. 143-148°C; ^{1}H NMR (600 MHz, $CDCl_3$) δ 8.01-7.99 (m, 4H) ,7.65 (s, 1H), 7.63 (d, J= 6Hz, 2H), 7.58 (t, *J* = 12 Hz, 1H), 7.53 (t, *J* = 12 Hz, 1H), 7.49-7.40 (m, 7H).

2d-i were also synthesized following this protocol.

***(E)*-2-benzoyl-1-oxoethyl-4-phenylbut-2-enoate (2c).**[2] A mixture of acetophenone (600 mg, 5.0 mmol), ethyl benzoyl acetate (990 mg, 5.0 mmol), iodine (1.40 g, 5.5 mmol), and CuO (440 mg, 5.5 mmol) in 20 mL DMSO was stirred at 70 °C for 15 h. The reaction mixture was cooled to room temperature, filtered through a pad of celite and eluted with ethyl acetate. To the filtrate, water was added and this aqueous phase was extracted with ethyl acetate. The organic layer was washed with saturated aqueous sodium thiosulphate, water and brine successively. After drying over Na_2SO_4 and evaporation, the crude product (1.3 gm, Yield: ~ 84%) was used for further reaction with 2-phenyl indole.

***(E)*-1,2-diphenyl-4-(pyridin-2-yl)but-2-ene-1,4-dione (2d).** Brownish yellow solid; Yield: 50% 745 mg. m.p. 142-147 °C; ^{1}H NMR (400 MHz, $CDCl_3$) δ 8.73 – 8.66 (m, 1H), 8.44 (s, 1H), 8.03-7.95 (m, 3H), 7.74 (t, *J* = 8 Hz, 1H), 7.66 (d, *J* = 8 Hz, 2H), 7.52-7.46 (m, 1H), 7.44-7.32 (m, 6H); ^{13}C NMR (400 MHz, $CDCl_3$): δ 198.38, 188.27, 156.41, 153.58, 148.96, 137.17, 136.18, 134.89, 133.44, 130.88, 129.19, 128.87, 128.77, 127.59, 127.31, 123.11, 120.16; HRMS (ESI) m/z: Calcd. for $C_{21}H_{16}NO_2$ $[M + H]^+$ 314.1181; found 314.1186.

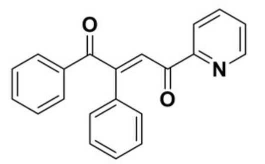

2d

***(E)*-4-(4-bromophenyl)-1,2-diphenylbut-2-ene-1,4-dione (2e).**[3] Yellowish white solid; Yield: 63% 1.17 g. m.p. 143-148 °C; 1H NMR (300 MHz, CDCl3) δ 7.97 (d, *J* = 6 Hz, 2H), 7.85 (d, *J* = 9 Hz, 2H), 7.65-7.58 (m, 4H), 7.56 (s, 1H), 7.52 (d, *J* = 9 Hz, 1H), 7.43 (t, *J* = 9 Hz, 5H).

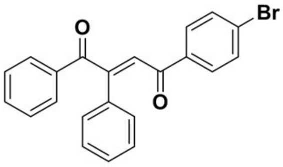

2e

***(E)*-4-(4-(tert-butyl)phenyl)-1,2-diphenylbut-2-ene-1,4-dione (2f).** Yellow solid; Yield: 59% 1.03 g. m.p. 158-163 °C; ^{1}H NMR (600 MHz, $CDCl_3$) δ 8.03 (d, *J* = 12Hz, 2H), 7.99 (d, *J* = 6Hz, 2H), 7.70 (s, 1H), 7.65 (d, *J* = 12 Hz, 2H), 7.50 (d, *J* = 12.0 Hz, 3H), 7.43-7.40 (m, 5H), 1.36 (s, 9H); ^{13}C NMR (600 MHz, $CDCl_3$): δ 197.64, 187.73, 157.16, 155.90, 136.17, 134.90, 134.74, 133.19, 130.61, 129.18, 128.72, 128.66, 128.64, 127.25, 125.69, 121.16, 35.15, 31.60, 31.06, 22.67, 14.17; HRMS (ESI) m/z: Calcd. for $C_{26}H_{24}O_2Na$ $[M + Na]^+$ 391.1674; found 391.1667.

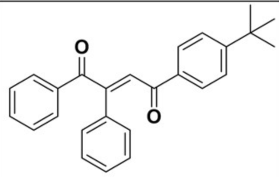

2f

***(E)*-4-(2,4-dimethoxyphenyl)-1,2-diphenylbut-2-ene-1,4-dione (2g).** Light yellow solid; Yield: 54% 956 mg. m.p. 151-156 °C; ^{1}H NMR (600 MHz, $CDCl_3$): δ 8.00 (d, *J* = 12 Hz, 2H), 7.72-7.69 (m, 2H), 7.59-7.56 (m, 2H), 7.50 (t, *J* = 6 Hz, 1H), 7.42-7.36 (m, 5H), 6.51-6.47 (m, 2H), 3.97 (s, 3H), 3.85 (s, 3H); ^{13}C NMR (600 MHz, $CDCl_3$): δ 197.84, 187.86, 164.84, 160.73, 152.72, 136.25, 135.33, 133.69, 132.92, 129.99, 128.97, 128.67, 128.59, 127.18, 121.18, 105.42, 98.45, 55.81, 55.54; HRMS (ESI) m/z: Calcd. for $C_{24}H_{21}O_4$ $[M + H]^+$ 373.1440; found 373.1436.

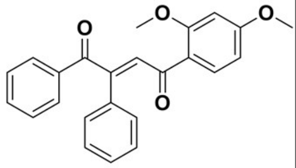

2g

***(E)*-4-(3-nitrophenyl)-1,2-diphenylbut-2-ene-1,4-dione (2h).** Cream yellow solid; Yield: 61% 1.04 g. m.p. 152-157 °C; ^{1}H NMR (600 MHz, $CDCl_3$) δ 8.81 (s, 1H), 8.41 (d, *J* = 6 Hz, 1H), 8.3 (d, *J* = 7.8 Hz, 1H), 7.98 (d, *J* = 12 Hz, 2H), 7.68-7.62 (m, 4H), 7.55 (t, *J* = 7.4 Hz, 1H), 7.46-7.41 (m, 5H); ^{13}C NMR (600

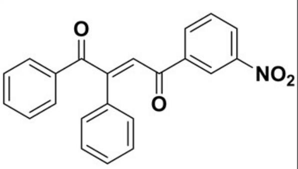

2h

MHz, $CDCl_3$): δ 197.06, 186.08, 158.48, 148.33, 138.50, 135.78, 134.25, 134.13, 133.58, 131.30, 130.03, 129.33, 128.84, 128.67, 127.47, 127.39, 123.28, 119.39; HRMS (ESI) m/z: Calcd. for $C_{22}H_{16}NO_4$ $[M + H]^+$ 358.1079; found 358.1082.

***(E)*-4-(2-nitrophenyl)-1,2-diphenylbut-2-ene-1,4-dione (2i).** Yellowish solid; Yield: 63% 1.07 g. m.p. 150-155 °C; ^1H NMR (400 MHz, $CDCl_3$) δ 8.06 (d, J = 12 Hz, 1H), 8.02 (d, J = 4 Hz, 1H), 8.0 (d, J = 4 Hz, 1H), 7.67 (m, 1H), 7.56 (m, 2H), 7.53-7.48 (m, 3H), 7.47-7.41 (m, 3H), 7.36 (m, 3H), 6.93 (s, 1H); ^{13}C NMR (400 MHz, $CDCl_3$): δ 196.60, 189.49, 155.34, 136.81, 135.59, 134.46, 134.26, 133.81, 131.14, 131.08, 129.28, 129.25, 128.99, 128.86, 127.44, 124.30, 123.14; HRMS (ESI) m/z: Calcd. for $C_{22}H_{16}NO_4$ $[M + H]^+$ 358.1079; found 358.1082.

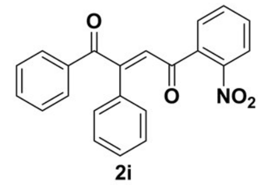

2i

Synthesis of 3-indolyl furanoids

Representative experimental procedure of 3db. To a stirred solution of 2-phenyl indole (**1d**, 19.4 mg, 0.1 mmol) and (*E*)-1,2,4-triphenylbut-2-ene-1,4-dione (**2b**, 37.4 mg, 0.12 mmol) in 1 mL dry dichloromethane, was added *p*-TsOH (9.5 mg, 0.05 mmol). The reaction mixture was then refluxed (40 °C) for 16 h. After completion of reaction (as monitored by TLC), the reaction mixture was diluted with dichloromethane (20 mL) and filtered. The filtrate was washed with water and the extracted organic layer was passed over anhydrous sodium sulphate. The solvent was evaporated to dryness under reduced pressure and the crude reaction mixture was purified by flash chromatography (silica gel 100-200 mesh; eluent: 0.2-0.5% ethyl acetate in hexane) to give the 2-phenyl-3-(2, 4, 5-triphenylfuran-3-yl)-1*H*-indole (**3db**, 39.5 mg, 81% yield) as off-white solid. A similar reaction procedure was followed for the synthesis of all other 3-indolyl furanoids.

2-Phenyl-3-(2,4,5-triphenylfuran-3-yl)-1*H*-indole (3db). Off-white solid; Yield: 81% 39.5 mg. m.p. 154-156 °C; ^1H NMR (400 MHz, DMSO-d_6): δ 11.49 (s, 1H), 7.49-7.43(m, 6H), 7.35 (d, J = 8.0 Hz, 1H), 7.28-7.21 (m, 5H), 7.19-7.09 (m, 5H), 7.07-6.99 (m, 4H), 6.87 (t, J = 8.0 Hz, 1H), 6.80 (d, J = 8.0 Hz, 2H); ^{13}C-NMR (100 MHz, DMSO-d_6) δ 148.5, 147.7, 136.7, 135.7, 133.3, 132.8, 130.9, 130.7, 129.7, 129.3, 129.1, 129.1, 129.1, 128.6, 128.1, 127.9, 127.7, 127.0, 126.9, 125.5, 124.6, 122.5, 120.1, 119.3, 118.7, 112.0, 103.8; HRMS (EI) m/z: Calcd. for $C_{36}H_{25}NO$ $[M]^+$ 487.1936; found 487.1933. HPLC analysis (Chiralcel OD-H, λ = 254 nm, 95:5 hexane: ethanol, flow rate: 0.5 mL min^{-1}, mode: Isocratic, column oven temp.: 25 °C): [t_R (+)] = 17.1 min, $[\alpha]_D^{25}$ +54.0 (c 0.5, Ethanol) [t_R (-)] = 34.3 min, $[\alpha]_D^{25}$ -53.0 (c 0.5, Ethanol).

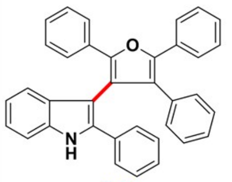

3db

3-(2,5-Diphenylfuran-3-yl)-1*H*-indole (3aa). Dark yellow solid; Yield: 78% 26.2 mg. m.p. 108-110 °C; ^1H NMR (400 MHz, $CDCl_3$): δ 8.26 (brs, 1H), 7.81 (d, J = 8.0 Hz, 2H), 7.7 (d, J = 8.0 Hz, 2H), 7.49 (d, J = 8.0 Hz, 1H), 7.46-7.42 (m, 3H), 7.32-7.27 (m, 3H), 7.25-7.17 (m, 3H), 7.11 (t, J = 8.0 Hz, 1H), 6.89 (s, 1H); ^{13}C NMR (100 MHz, $CDCl_3$) δ 152.3, 148.3, 136.2, 131.6, 130.8, 128.8, 128.3, 127.5, 127.1, 126.6, 125.6, 123.9 , 122.9, 122.5,

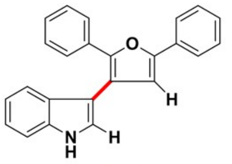

3aa

120.4, 120.1, 117.1, 111.3, 110.8, 109.9; HRMS (ESI) m/z: Calcd. for $C_{24}H_{18}NO$ [M + H]$^+$ 336.1388; found 336.1383.

3-(2,5-Diphenylfuran-3-yl)-2-methyl-1*H*-indole (3ba). Orange-white solid; Yield: 80% 28 mg. m.p. 92-96 ^oC; ^1H NMR (400 MHz, $CDCl_3$): δ 8.00 (brs, 1H), 7.83 (d, *J* = 8.0 Hz, 2H), 7.64 (d, *J*= 8.0 Hz, 2H), 7.45 (t, *J* = 8.0 Hz, 3H), 7.38-7.28 (m, 3H), 7.24-7.17 (m, 3H), 7.10 (t, *J* = 8.0 Hz, 1H), 6.87 (s, 1H), 2.25 (s, 3H); ^{13}C NMR (100 MHz, $CDCl_3$): δ 152.2, 148.6, 135.6, 132.5, 131.8, 130.8, 128.8, 128.4, 128.3, 127.4, 126.9, 125.0, 123.8, 121.6 , 119.9, 119.4, 116.8, 111.0, 110.3, 106.5, 12.6; HRMS (ESI) m/z: Calcd. for $C_{25}H_{20}NO$ [M+H]$^+$ 350.1545; found 350.1537.

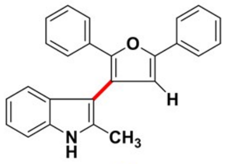

3ba

Ethyl 3-(2,5-diphenylfuran-3-yl)-1*H*-indole-2-carboxylate (3ca). Off-white solid; Yield: 69% 28.1 mg. m.p. 109-111 ^oC; ^1H NMR (300 MHz, $CDCl_3$): δ 9.12 (brs, 1H), 7.80 (d, *J* = 9 Hz, 2H), 7.55- 7.53 (m, 2H), 7.45 (q, *J* = 12.0, 6.0 Hz, 4H), 7.38-7.29 (m, 2H), 7.20-7.06 (m, 4H), 6.85 (s, 1H), 4.17 (d, *J* = 6.0 Hz, 2H), 1.08 (t, *J* = 6.0 Hz, 3H); ^{13}C NMR (75 MHz, $CDCl_3$): δ 162.4, 151.8, 149.2, 136.2, 131.4, 130.7, 128.7, 128.3, 127.5, 127.4, 127.0, 125.8, 124.9, 123.9, 123.7, 122.1, 120.9, 115.7, 115.4, 111.9, 111.5, 61.1, 13.9; HRMS (ESI) m/z: Calcd. for $C_{27}H_{22}NO_3$ [M + H]$^+$ 408.1600; found 408.1600.

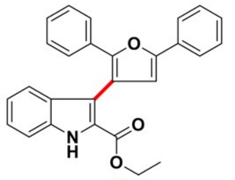

3ca

3-(2,5-Diphenylfuran-3-yl)-2-phenyl-1*H*-indole (3da). White solid; Yield: 81% 33.3 mg. m.p. 115-118 ^oC; ^1H NMR (400 MHz, $CDCl_3$): δ 8.4 (brs, 1H), 7.76 (d, *J* = 8.0 Hz, 2H), 7.61-7.55 (m, 4H), 7.47 (d, *J* = 8.0 Hz, 1H), 7.41 (t, *J* = 8.0 Hz, 2H), 7.34-7.28 (m, 4H), 7.24-7.00 (m, 6H), 6.68 (s, 1H); ^{13}C NMR (100 MHz, $CDCl_3$): δ 152.3, 149.1, 136.0, 134.6, 132.3, 131.2, 130.6, 128.7, 128.6, 128.2, 127.6, 127.3, 127.0 , 126.8, 124.7, 123.7, 122.8, 120.3, 116.7, 111.2, 110.8, 106.7; HRMS (EI) m/z: Calcd. for $C_{30}H_{21}NO$ [M]$^+$ 411.1623; found 411.1621.

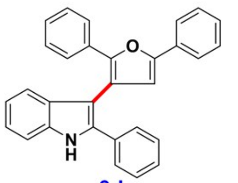

3da

3-(2,4,5-Triphenylfuran-3-yl)-1*H*-indole (3ab). Pale yellow solid; Yield: 75% 31 mg. m.p. 123-125 ^oC; ^1H NMR (600 MHz, DMSO-d_6): δ 7.50 (dd, *J* = 12.0, 6.0 Hz, 4H), 7.36 (d, *J* = 6.0 Hz, 1H), 7.31 (brs, 2H), 7.24-7.16 (m, 10H), 7.09 (d, *J* = 6 Hz, 1H), 7.04 (brs, 1H), 6.94 (brs, 1H); ^{13}C NMR (150 MHz, DMSO-d_6): δ 148.1, 147.2, 136.4, 133.5, 131.0, 130.7, 130.3 , 129.0, 128.9, 128.8, 127.9, 127.8, 127.7, 127.0, 126.9, 125.6, 125.5, 125.1, 121.6, 119.4, 118.6, 112.1, 105.9; HRMS (ESI) m/z: Calcd. for $C_{30}H_{22}NO$ [M+H]$^+$ 412.1701; found 412.1703.

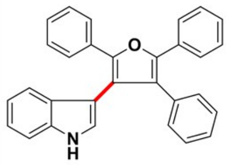

3ab

2-Methyl-3-(2,4,5-triphenylfuran-3-yl)-1*H*-indole (3bb). Dark brown solid; Yield: 76% 32.5 mg. m.p. 155-157 ^oC; ^1H NMR (600 MHz, (CD_3OD): δ 7.59 (dd, *J* = 12.0, 6.0 Hz, 4H), 7.34 -7.26 (m, , 4H), 7.23-7.13 (m, 9H), 7.05 (t, *J* = 6.0 Hz, 1H), 6.91 (t, *J* = 9.0 Hz, 1H), 2.00 (s, 3H); ^{13}C NMR (150 MHz, CD_3OD): δ 148.7 , 147.2, 136.0, 133.7, 133.5, 131.2, 131.0, 129.5, 128.5, 128.0,

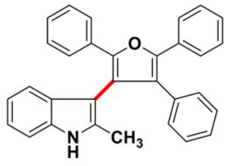

3bb

127.9, 127.8, 127.0, 126.7, 126.6, 126.5, 125.4, 124.3, 120.2, 118.6, 118.1, 117.6, 110.0, 103.5, 10.6 ; HRMS (ESI) m/z: Calcd. for $C_{31}H_{23}NO$ $[M]^+$ 425.1780; found 425.1772. HPLC analysis (Chiralcel OD-H, λ = 254 nm, 98:2 hexane: Isopropanol, flow rate: 0.5 mL min^{-1}, mode: Isocratic, column oven temp.: 15 °C): [t_R (+)] = 39.38 min, [t_R (-)] = 45.01 min.

2-(Naphthalen-2-yl)-3-(2,4,5-triphenylfuran-3-yl)-1*H*-indole (3eb). Pale yellow solid; Yield: 76% 41 mg. m.p. 182-184 °C; ^1H NMR (600 MHz, DMSO-d_6): δ 11.7 (s, 1H), 8.01 (s, 1H), 7.81 (t, *J* = 6.0 Hz, 2H), 7.74 (d, *J* = 6.0 Hz, 1H), 7.69 (d, *J* = 6.0 Hz, 1H), 7.55 (d, *J* = 6.0 Hz, 2H), 7.50 (d, J = 6.0 Hz, 2H), 7.47-7.43 (m, 3H), 7.31 (t, *J* = 6.0 Hz, 2H), 7.27-7.19 (m, 4H), 7.14 (q, *J* = 6.0 Hz, 2H), 7.06 (t, *J* = 6.0 Hz, 1H), 6.98 (t, *J* = 6.0 Hz, 2H), 6.94 (t, *J* = 6.0 Hz, 1H), 6.87 (d, *J* = 6.0 Hz, 2H), ^{13}C NMR (150 MHz, DMSO-d_6): δ 148.6, 147.7, 136.8, 135.5, 133.2, 133.2, 132.3, 130.8, 130.6, 130.3, 129.6, 129.3, 129.0, 128.5, 128.4, 128.2, 128.0, 127.9, 127.8, 127.6, 127.0, 126.6, 125.5, 125.4, 124.8, 124.6, 122.6, 120.1, 119.3, 118.6, 111.9, 104.3; HRMS (EI) m/z: Calcd. for $C_{40}H_{27}NO$ $[M]^+$ 537.2093; found 537.2095. HPLC analysis (Chiralcel OD-H, λ = 254 nm, 95:5 hexane: ethanol, flow rate: 1.0 mL min^{-1}, mode: Isocratic, column oven temp.: 20 °C): [t_R (+)] = 10.2 min, $[\alpha]_D^{25}$ +50.0 (c 0.5, Ethanol) [t_R (-)] = 13.6 min.

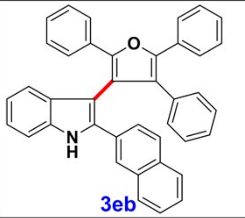
3eb

Ethyl 2,5-diphenyl-4-(2-phenyl-1*H*-indol-3-yl)furan-3-carboxylate (3dc). Off-white solid; Yield: 68% (33 mg) m.p. 131-133 °C; ^1H NMR (300 MHz, $CDCl_3$): δ 8.39 (s, 1H), 8.00 (d, *J* = 6.0 Hz, 2H), 7.54–7.35 (m, 10H), 7.29 (d, *J* = 6.0 Hz, 1H), 7.23–7.18 (m, 2H), 7.17–7.13 (m, 3H), 7.06 (t, *J* = 6 Hz, 1H), 3.84 (q, *J* = 12 Hz, 2H) 0.66 (t, *J* = 6 Hz, 3H) ; ^{13}C NMR (75 MHz, $CDCl_3$): δ 164.2, 154.6, 149.4 , 135.9, 135.0, 132.4, 130.2, 129.8, 129.5, 129.0, 128.7, 128.3, 128.24, 127.6, 127.5, 127.4, 126.7, 125.0, 122.5, 120.2, 120.0, 117.8, 116.0, 110.6, 105.3, 60.2, 13.2; HRMS (EI) m/z: Calcd. for $C_{33}H_{25}NO_3$ $[M]^+$ 483.1834; found 483.1839. HPLC analysis (Chiralcel OD-H, λ = 254 nm, 95:5:0.1 hexane: isopropanol: TFA, flow rate: 0.8 mL min^{-1}, mode: Isocratic, column oven temp.: 20 °C): [t_R (+)] = 19.3 min, $[\alpha]_D^{25}$ +47.0 (c 0.5, Ethanol) [t_R (-)] = 23.1 min.

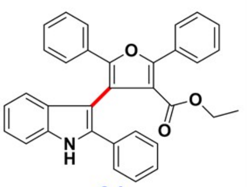
3dc

3-(4,5-Diphenyl-2-(pyridin-2-yl)furan-3-yl)-2-phenyl-1*H*-indole (3dd). Dark yellow solid; Yield: 78% 38 mg. m.p. 176-178 °C; ^1H NMR (600 MHz, CD_3OD): δ 8.58 (d, *J* = 6.0 Hz, 1H), 8.07 (d, *J* = 6.0 Hz, 1H), 7.69 (d, *J* = 6.0 Hz, 2H), 7.40 (d, *J* = 7.2 Hz, 3H), 7.36 (d, *J* = 6.0 Hz, 2H), 7.35-7.21 (m, 7H), 7.10 (t, *J* = 6.0 Hz, 2H), 7.01 (t, *J* = 6.0 Hz, 4H), 6.79 (d, *J* = 6.0 Hz, 2H); ^{13}C NMR (150 MHz, CD_3OD): δ 149.8, 148.7, 148.5, 136.9, 136.7, 133.9, 132.8, 132.6, 132.5, 130.4, 129.4, 128.6, 128.0, 127.9, 127.6, 127.5, 127.0, 126.7, 126.6, 126.5, 125.8, 122.4, 121.9, 121.8, 120.7, 119.5, 118.7, 111.0; HRMS (EI) m/z: Calcd. for $C_{35}H_{24}N_2O$ $[M]^+$ 488.1889; found 488.1891. HPLC analysis (Chiralcel OD-H, λ = 254 nm, 0.3% diethylamine in hexane: ethanol = 90:10, flow rate: 1.0 mL min^{-1}, mode: Isocratic, column oven temp.: 25 °C): [t_R (+)] = 7.7 min, $[\alpha]_D^{25}$ +51.0 (c 0.5, Ethanol) [t_R (-)] = 11.9 min.

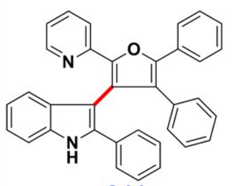
3dd

3-(2-(4-Bromophenyl)-4,5-diphenylfuran-3-yl)-2-phenyl-1*H*-indole (3de). Off-white solid; Yield: 82% (46.5 mg) m.p. 159-161 °C; ^1H NMR (600 MHz, CD_3OD): δ 7.50 (dd, *J* = 12.0 Hz, 6.0 Hz, 4H), 7.40 (d, *J* = 12 Hz, 1H), 7.34 (d, *J* = 6 Hz, 2H), 7.30-7.28 (m, 2H), 7.21-7.17

(m, 6H), 7.15-7.10 (m, 2H), 7.01 (t, J = 12 Hz, 1H), 6.95-6.90 (m, 3H), 6.71 (d, J = 6 Hz, 2H); ^{13}C NMR (150 MHz, CD_3OD): δ 148.0, 147.6, 136.6, 135.8, 133.0, 132.7, 131.1, 130.6, 130.1, 129.3, 128.7, 128.0, 127.5, 127.1, 126.9, 126.7, 126.6, 126.4, 125.9, 125.3, 121.8, 120.3, 119.4, 119.1, 118.9, 110.9, 103.4; HRMS (ESI) m/z: Calcd. for $C_{36}H_{24}BrNONa$ [M + Na]$^+$ 588.0939; found 588.0938. HPLC analysis (Chiralcel OD-H, λ = 254 nm, 95:5 hexane: ethanol, flow rate: 0.5 mL min^{-1}, mode: Isocratic, column oven temp.: 25 °C): racemic, [t_R (+)] = 18.0 min $[\alpha]_D^{25}$ +56.0 (c 0.5, Ethanol), [t_R (-)] = 30.0 min.

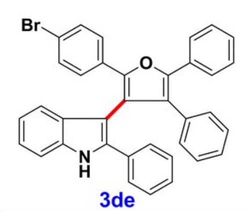

3de

3-(2-(4-(*tert*-butyl)phenyl)-4,5-diphenylfuran-3-yl)-2-phenyl-1*H*-indole (3df). Pale yellow solid; Yield: 80% (43.5 mg) m.p. 141-143 °C; ^{1}H NMR (600 MHz, CD_3OD): δ 7.57 (d, J = 6.0 Hz, 2H), 7.50 (d, J = 6.0 Hz, 2H), 7.39 (t, J = 6.0 Hz, 3H), 7.24-7.10 (m, 10H), 7.02 (t, J = 6.0 Hz, 1H), 6.93 (q, J = 12 Hz, 3H), 6.72 (d, J = 12.0 Hz, 2H), 1.24 (s, 9H); ^{13}C NMR (150 MHz, CD_3OD): δ 149.8, 149.0, 147.2, 136.6, 135.69, 133.3, 132.9, 131.0, 129.3 , 129.2, 128.3, 127.9, 127.4, 126.8, 126.7, 126.5, 126.3, 125.1, 124.9, 124.1, 121.6, 119.2, 119.0, 117.6 , 110.7, 104.0, 33.9, 30.2; HRMS (ESI) m/z: Calcd. for $C_{40}H_{33}NONa$ [M + Na]$^+$ 566.2460; found 566.2457. HPLC analysis (Chiralcel OD-H, λ = 254 nm, 95:5 hexane: ethanol, flow rate: 0.5 mL min^{-1}, mode: Isocratic, column oven temp.: 25 °C): racemic, [t_R (+)] = 13.9 min, $[\alpha]_D^{25}$ +48.0 (c 0.5, Ethanol) [t_R (-)] = 32.3 min.

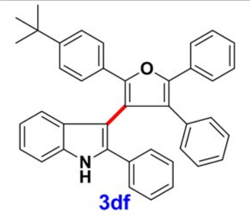

3df

3-(2-(2,4-Dimethoxyphenyl)-4,5-diphenylfuran-3-yl)-2-phenyl-1*H*-indole (3dg). Off-white solid; Yield: 76% (42 mg) m.p. 142-144 °C; ^{1}H NMR (600 MHz, CD_3OD): δ 7.45 (d, J = 6.0 Hz, 2H), 7.41 (d, J = 6.0 Hz, 2H), 7.28 (d, J = 6.0 Hz, 1H), 7.23-7.11 (m, 8H), 6.99 (t, J = 6.0 Hz, 2H), 6.93 (t, J = 6.0 Hz, 2H), 6.81 (t, J = 6.0 Hz, 3H), 6.35- 6.32 (m, 2H), 3.68 (s, 3H), 3.33 (s, 3H); ^{13}C NMR (150 MHz, CD_3OD): δ 161.1, 158.4, 148.3, 147.4, 136.3, 135.1, 133.9, 133.3, 131.4, 130.6, 129.5, 129.4, 127.8, 127.7, 127.4 , 126.9, 126.4, 126.3, 126.0, 125.2, 125.1, 121.1, 119.0, 118.8, 118.6, 113.6, 110.3, 104.9, 104.4, 98.3, 54.3, 54.2; HRMS (EI) m/z: Calcd. for $C_{38}H_{29}NO_3$ [M]$^+$ 547.2147; found 547.2139. HPLC analysis (Chiralcel OD-H, λ = 254 nm, 95:5 hexane: ethanol, flow rate: 0.5 mL min^{-1}, mode: Isocratic, column oven temp.: 25 °C): racemic, [t_R (+)] = 11.9 min, $[\alpha]_D^{25}$ +42.0 (c 0.5, Ethanol) [t_R (-)] = 17.0 min .

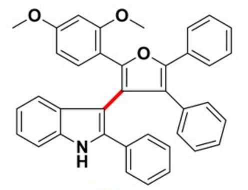

3dg

3-(2-(3-Nitrophenyl)-4,5-diphenylfuran-3-yl)-2-phenyl-1*H*-indole (3dh). Dark yellow solid; Yield: 82% (43.7 mg) m.p. 167-169 °C; ^{1}H NMR (600 MHz, DMSO-d_6): δ 11.67 (s, 1H), 8.2 (s, 1H), 7.97 (d, J = 6.0 Hz, 1H), 7.91 (d, J = 6.0 Hz, 1H), 7.51 (q, J = 12.0 Hz, 5H), 7.42 (d, J = 12.0 Hz, 1H), 7.34 (t, J = 6.0 Hz, 2H), 7.30 (t, J = 6.0 Hz, 3H), 7.20 (q, J = 6.0 Hz, 2H), 7.14-7.07 (m, 4H), 6.91 (t, J = 6.0 Hz, 3H); ^{13}C NMR (150 MHz, DMSO-d_6): 148.8, 148.4, 146.0, 136.7, 136.0, 132.7, 132.5, 132.0, 130.7, 130.2, 130.0, 129.6 , 129.1, 129.0 , 128.7 , 128.0, 127.8, 127.0, 126.9, 125.8 , 122.6, 122.0, 121.0, 120.2, 119.1, 118.6, 112.0, 102.6 ; HRMS (ESI) m/z: Calcd. for $C_{36}H_{24}N_2O_3Na$ [M + Na]$^+$ 555.1685; found 555.1698. HPLC analysis (Chiralcel OD-H, λ = 254 nm, 90:10 hexane: ethanol, flow rate: 0.5 mL min^{-1}, mode: Isocratic, column oven temp.: 25 °C): racemic, [t_R (+)] = 17.8 min, $[\alpha]_D^{25}$ +52.0 (c 0.5, Ethanol) [t_R (-)] = 29.0 min.

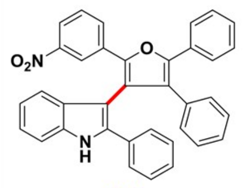

3dh

3-(2-(2-Nitrophenyl)-4,5-diphenylfuran-3-yl)-2-phenyl-1*H*-indole (3di). Dark yellow solid; Yield: 85% (45.3 mg) m.p. 167-169 °C; ^{1}H NMR (600 MHz, $CDCl_3$): δ 8.16 (brs, 1H), 7.68 (d, J = 12.0 Hz, 1H), 7.49-7.46 (m, 2H), 7.40-7.36 (m, 2H), 7.34-7.29 (m, 3H), 7.25-7.15 (m, 9H), 7.07- 6.96 (m, 4H), 6.90- 6.86 (m, 2H); ^{13}C NMR (150 MHz, $CDCl_3$): δ 150.0, 148.2, 145.0, 136.0, 135.9, 132.8, 132.3, 131.8, 130.6, 129.8, 129.8, 129.4, 128.7, 128.5, 128.1, 128.0, 127.7, 127.6, 127.0, 126.9, 125.8, 125.7, 124.9, 124.1, 122.6, 120.8, 120.5, 120.0, 110.8, 103.9; HRMS (ESI) m/z: Calcd. for $C_{36}H_{25}N_2O_3$ $[M + H]^+$ 533.1865; found 533.1866. HPLC analysis (Chiralcel OD-H, λ = 254 nm, 90:10 hexane: ethanol, flow rate: 0.5 mL min^{-1}, mode: Isocratic, column oven temp.: 25 °C): racemic, [t_R (+)] = 19.1 min, $[\alpha]_D^{25}$ +46.0 (c 0.5, Ethanol) [t_R (-)] = 27.5 min.

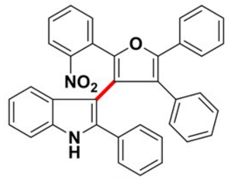

3di

Section C: ^{1}H NMR, ^{13}C NMR Spectra, and HPLC profiles

^{1}H NMR, ^{13}C NMR Spectra of synthesized enediones

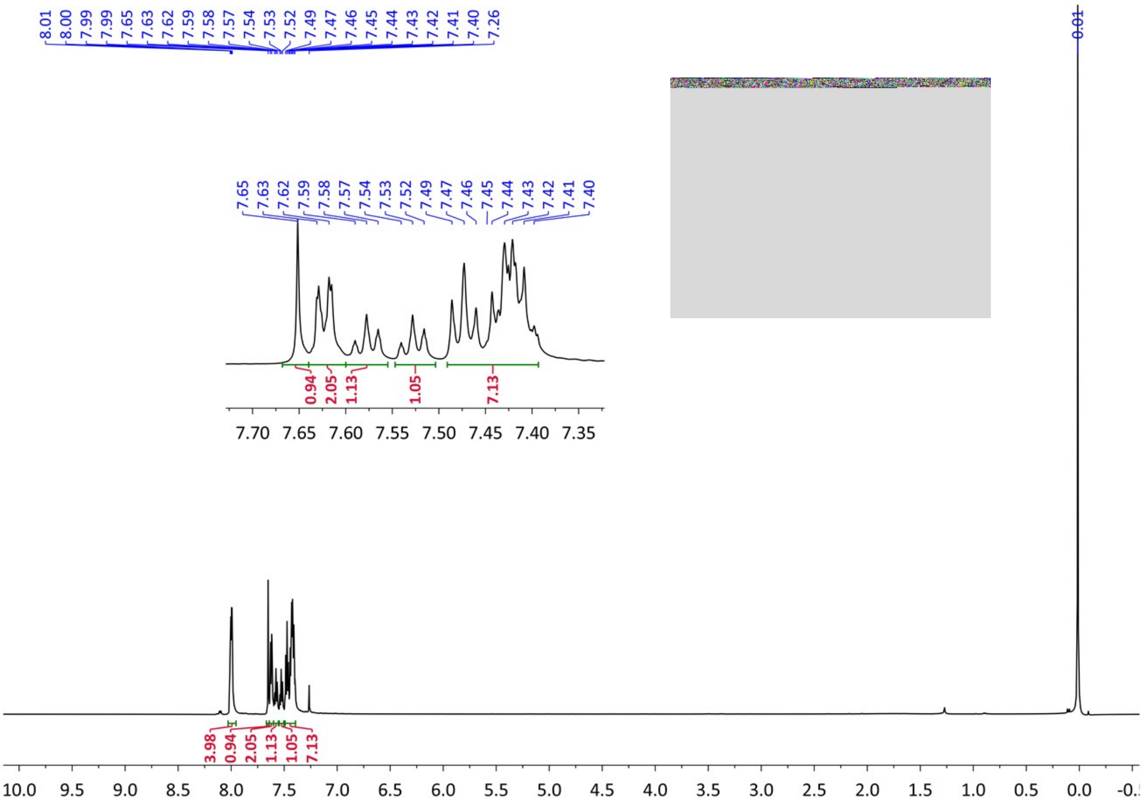

Figure S1: ^{1}H NMR spectrum of **2b** in $CDCl_3$ at 600 MHz

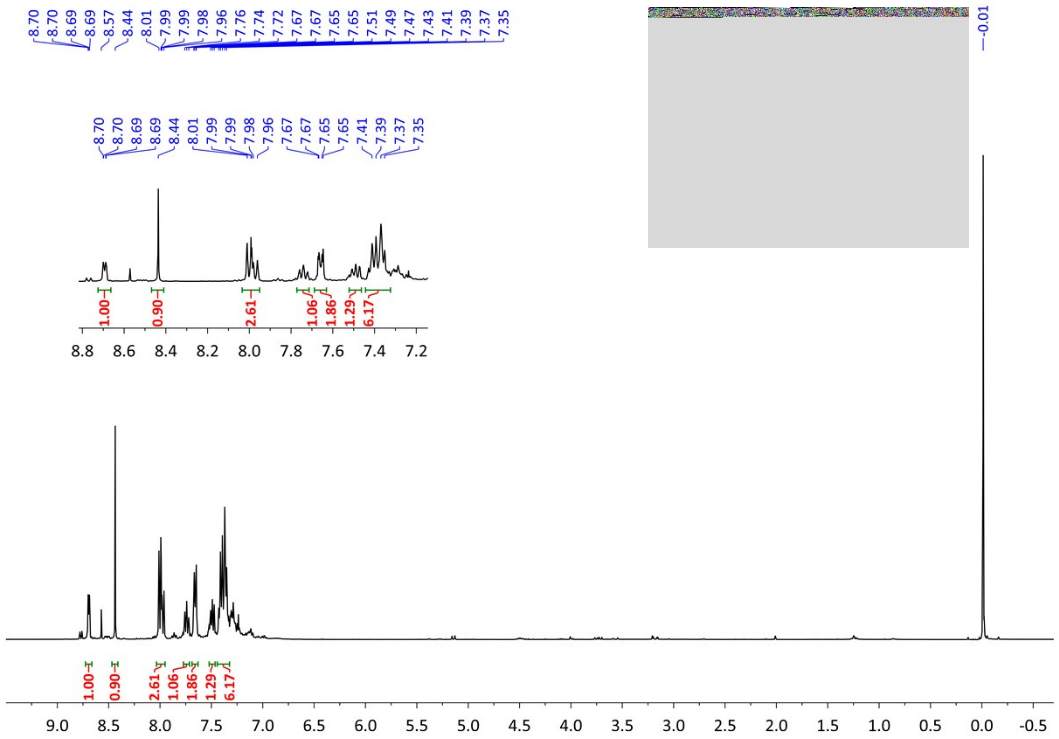

Figure S2: ^{1}H NMR spectrum of **2d** in $CDCl_3$ at 400 MHz

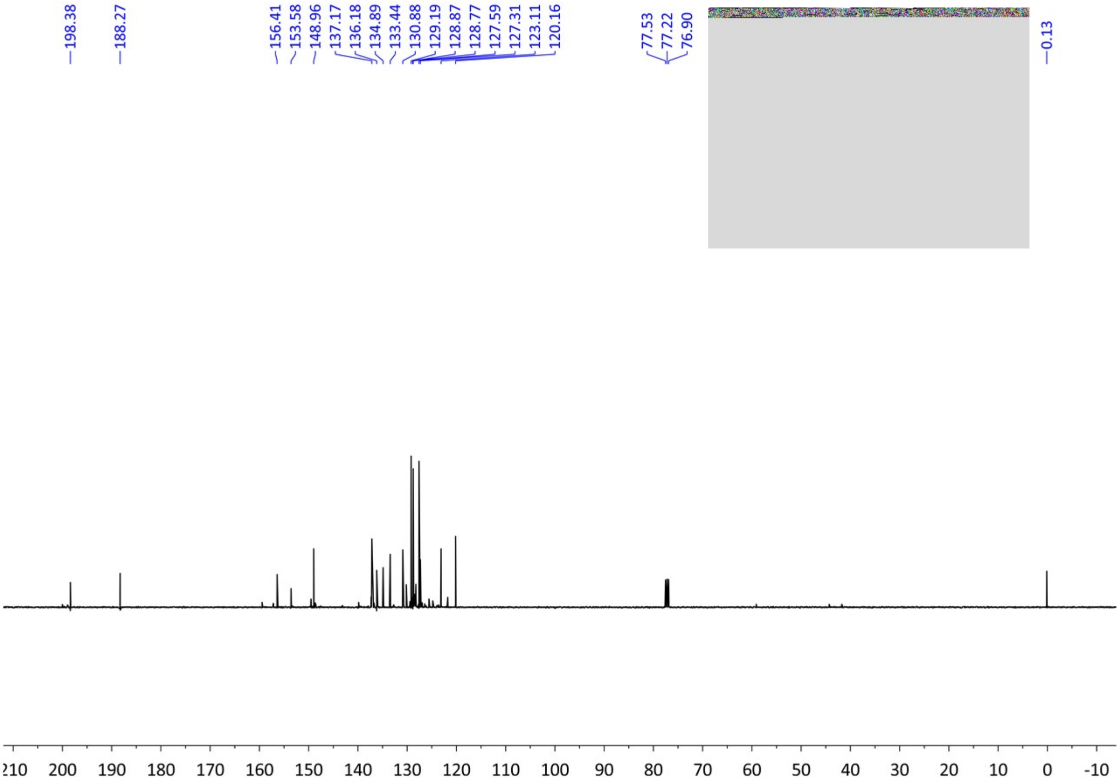

Figure S3: ^{13}C NMR spectrum of **2d** in $CDCl_3$ at 100 MHz

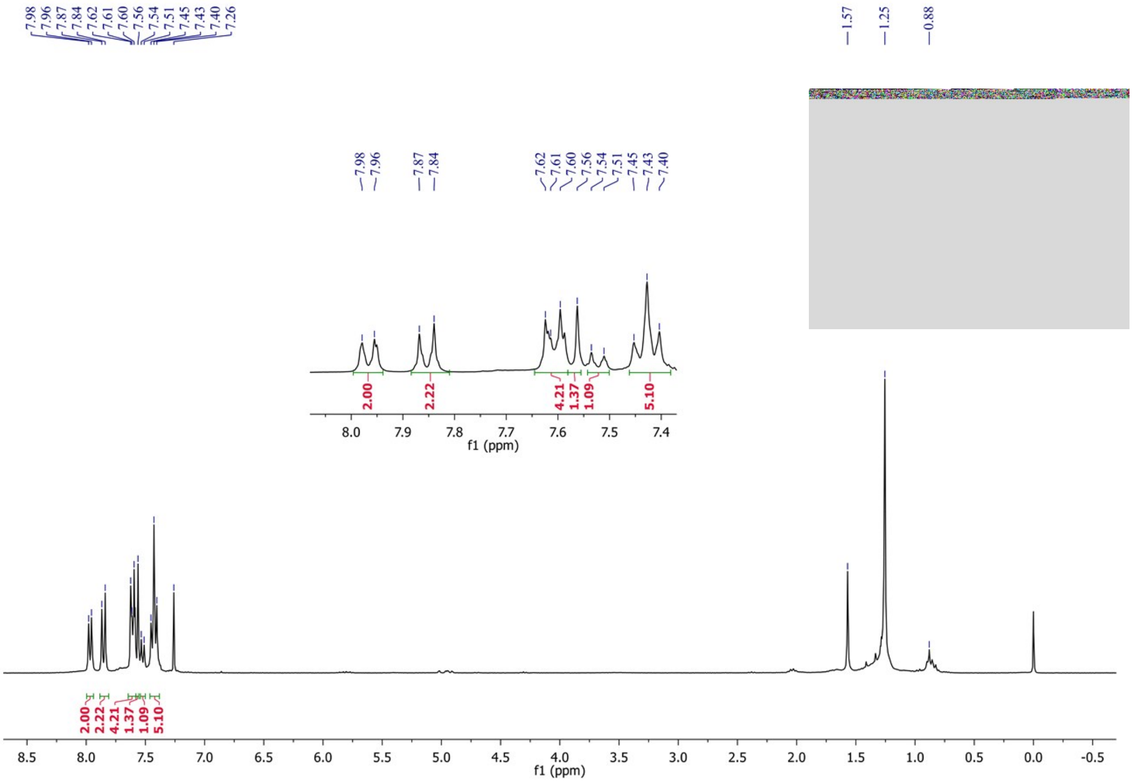

Figure S4: ^{1}H NMR spectrum of **2e** in $CDCl_3$ at 300 MHz

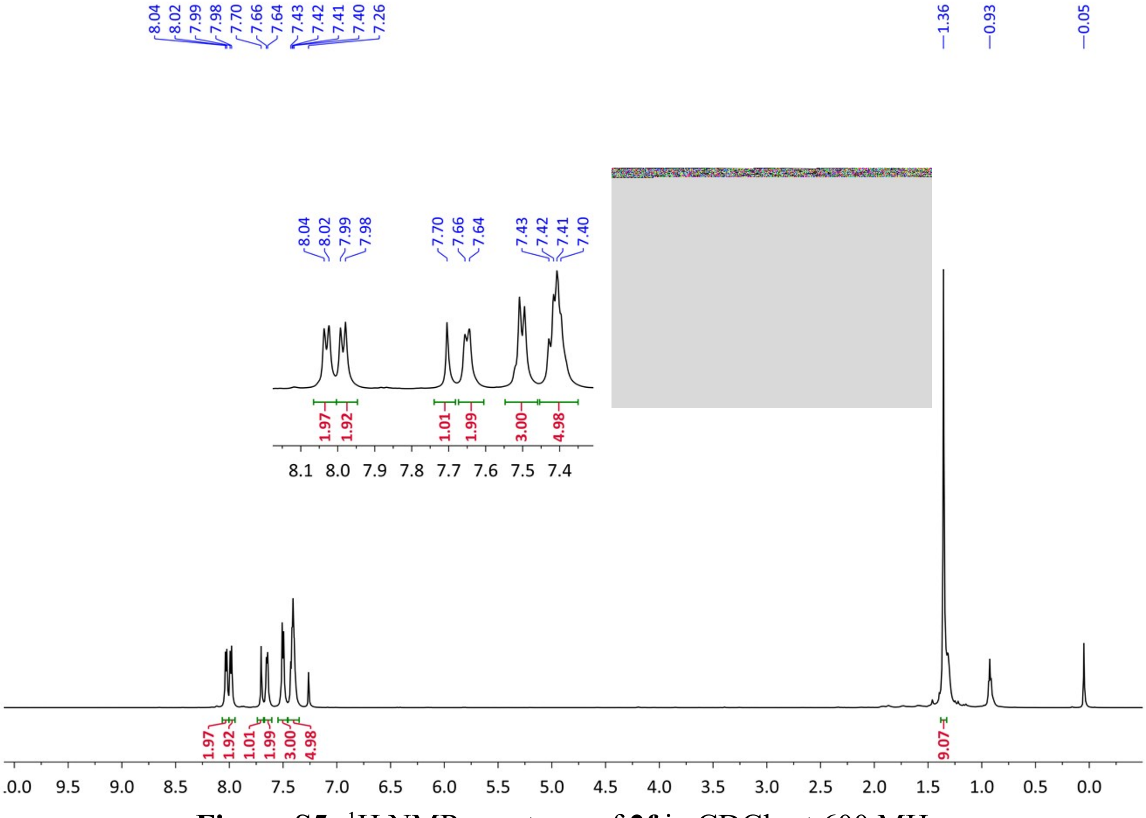

Figure S5: ^{1}H NMR spectrum of **2f** in $CDCl_3$ at 600 MHz

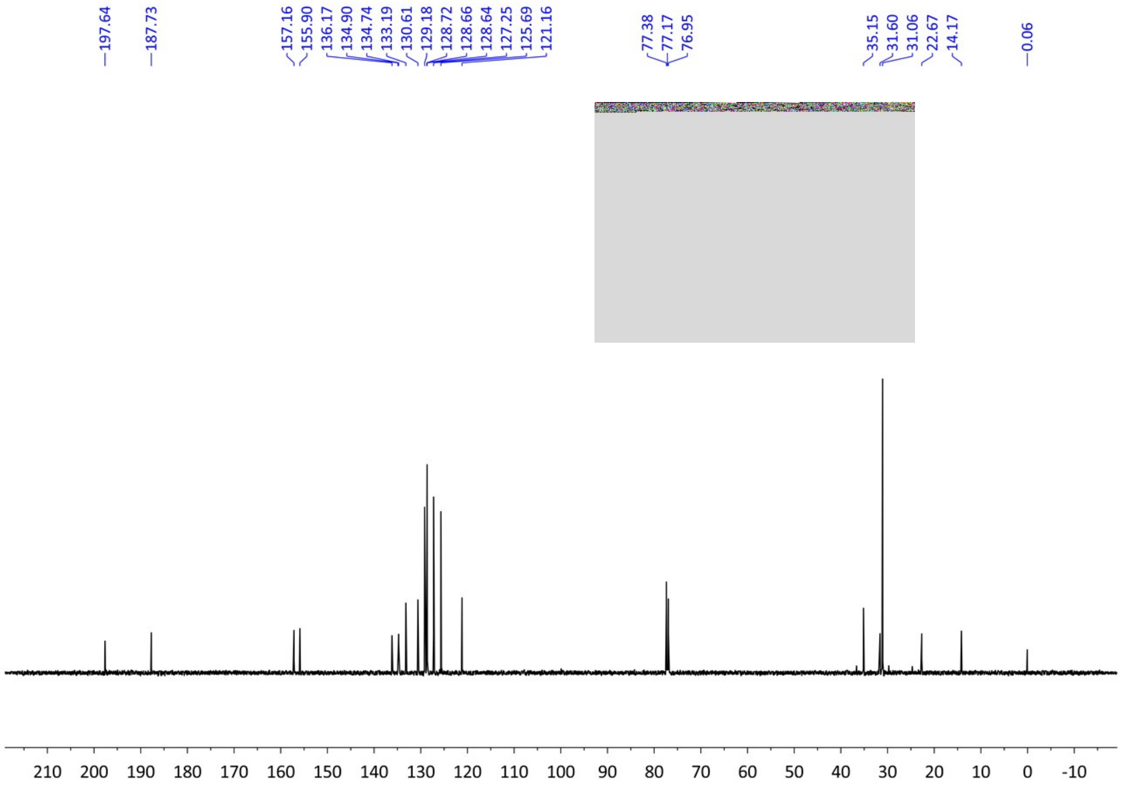

Figure S6: ^{13}C NMR spectrum of **2f** in $CDCl_3$ at 150 MHz

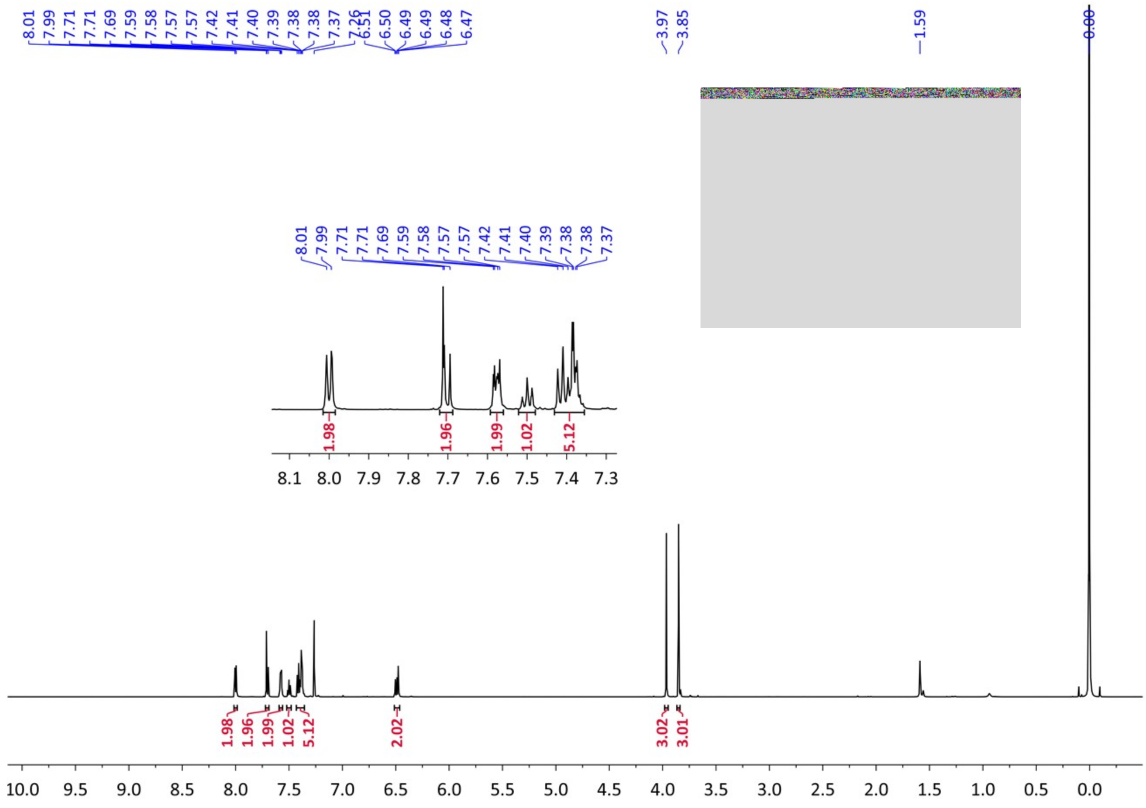

Figure S7: ^{1}H NMR spectrum of **2g** in $CDCl_3$ at 600 MHz

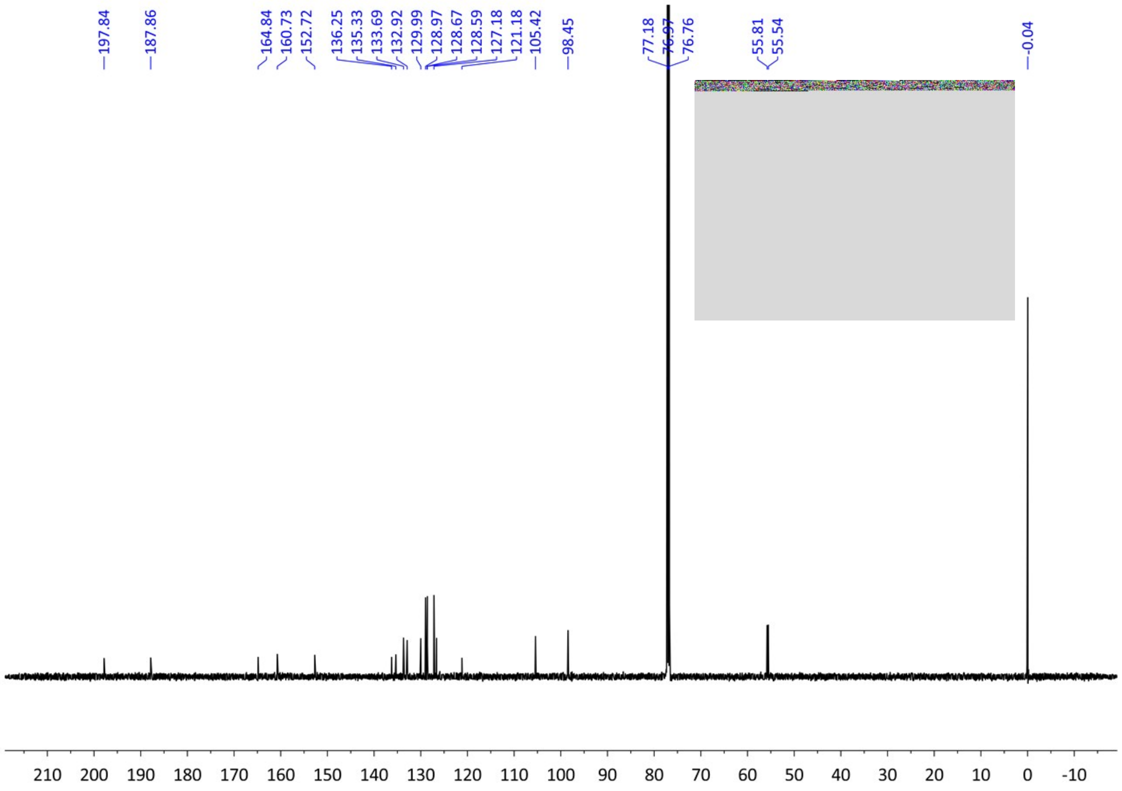

Figure S8: ^{13}C NMR spectrum of **2g** in $CDCl_3$ at 150 MHz

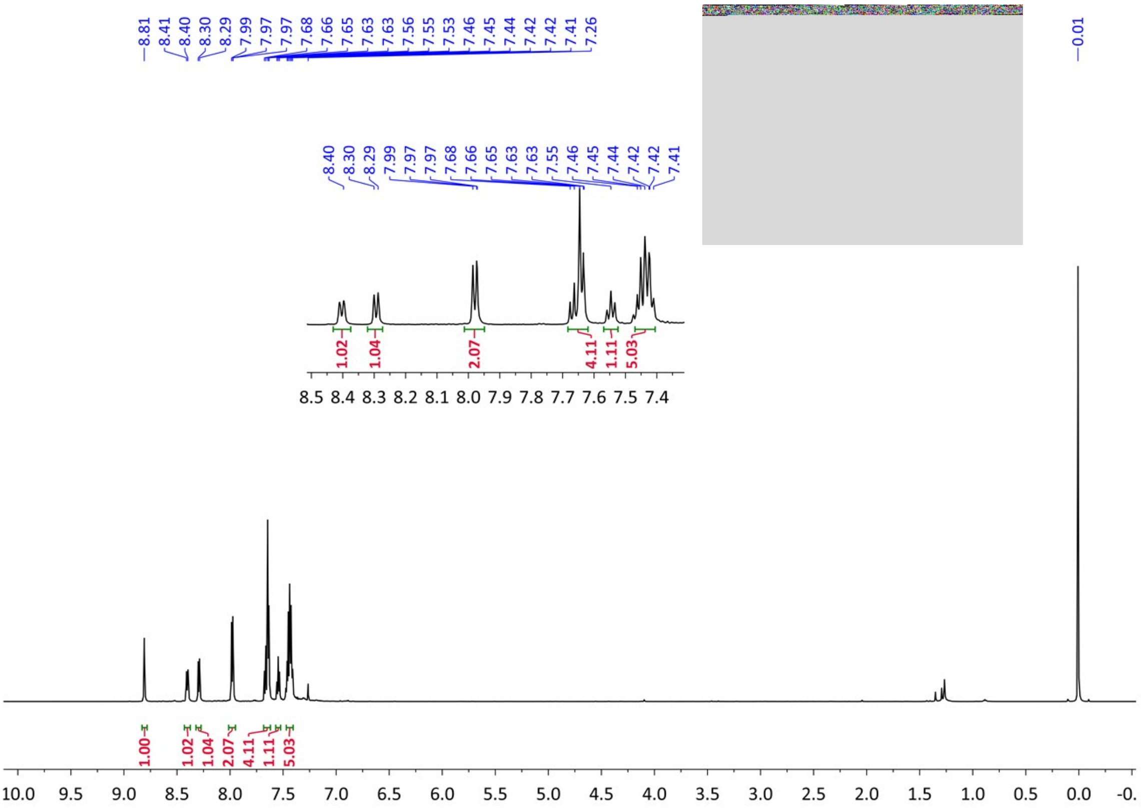

Figure S9: ^{1}H NMR spectrum of **2h** in $CDCl_3$ at 600 MHz

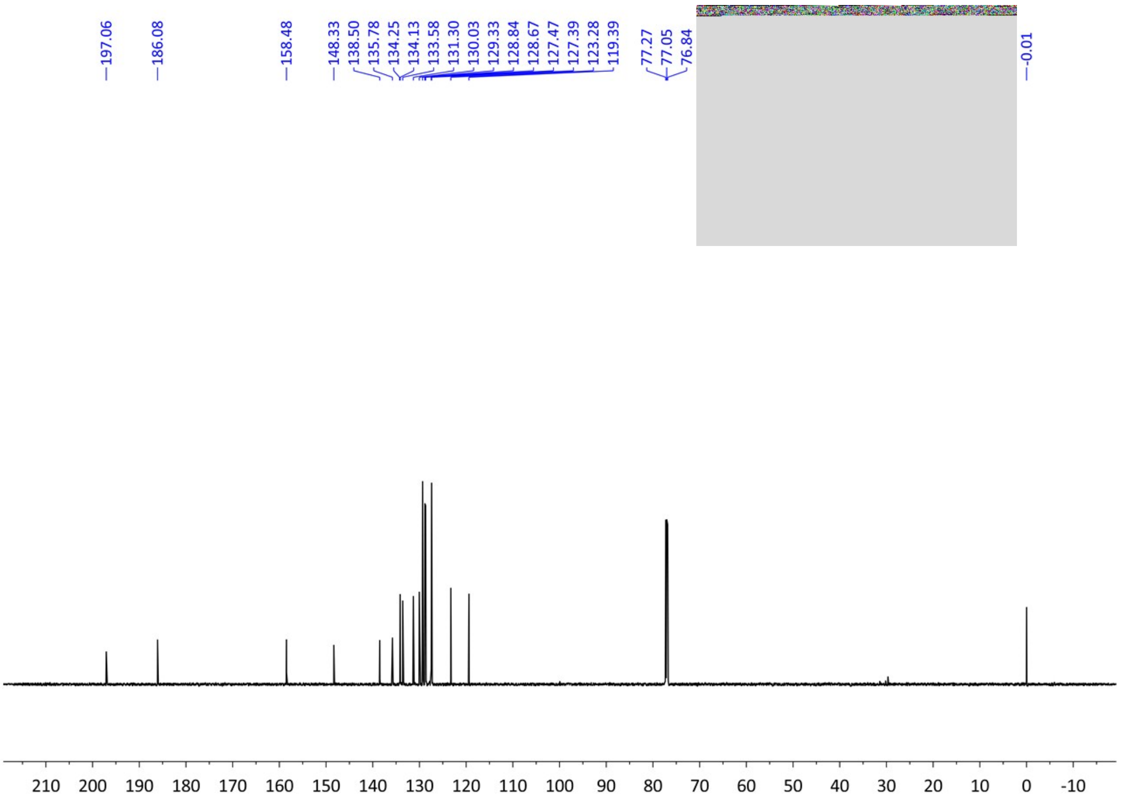

Figure S10: ^{13}C NMR spectrum of **2h** in $CDCl_3$ at 150 MHz

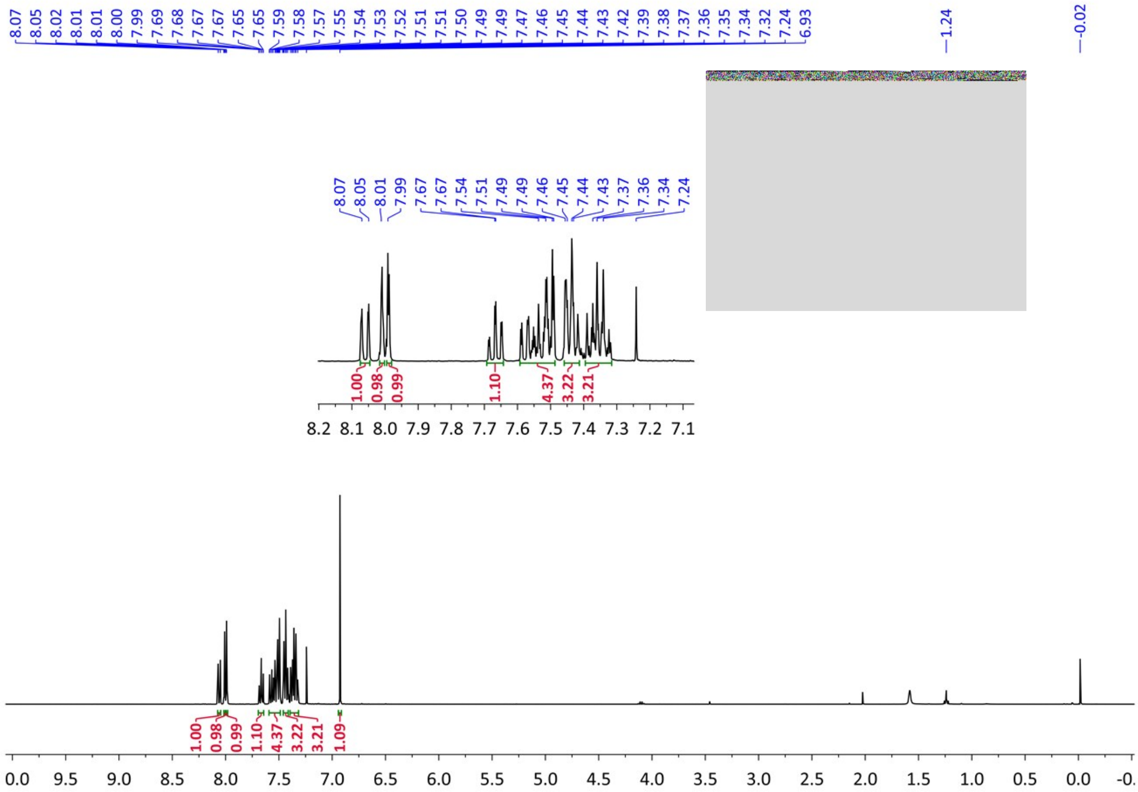

Figure S11: ^{1}H NMR spectrum of **2i** in $CDCl_3$ at 400 MHz

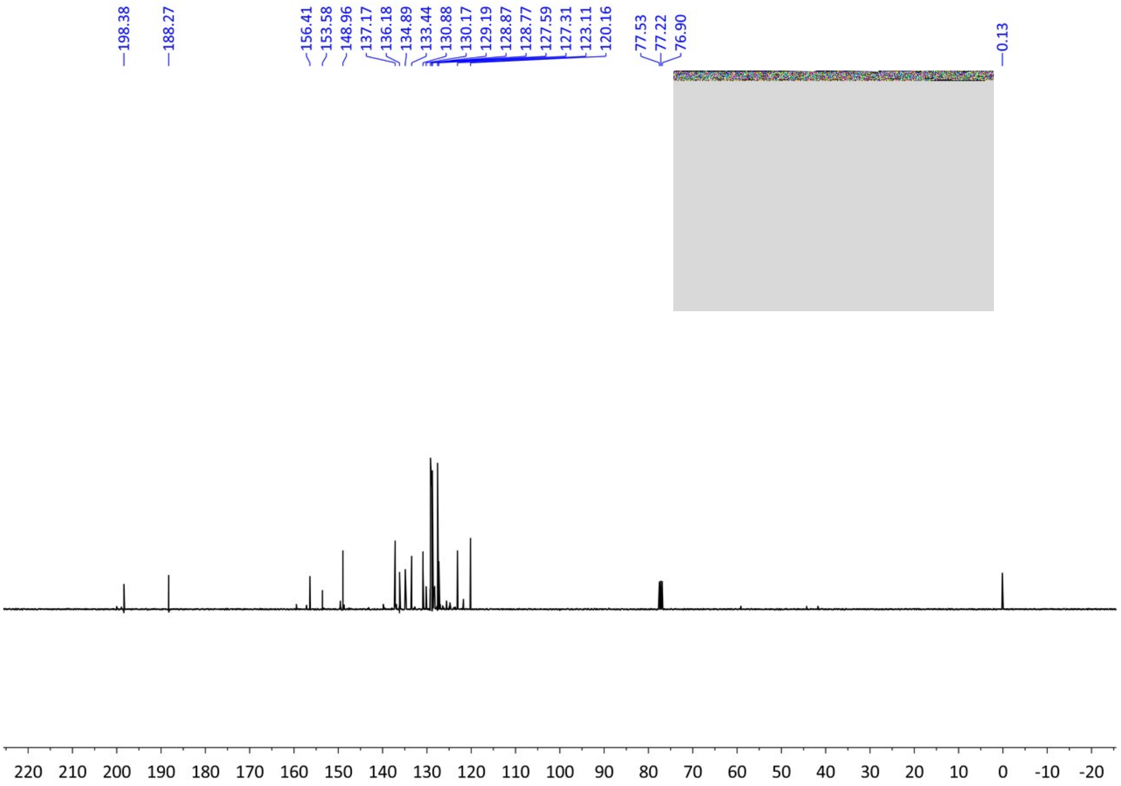

Figure S12: ^{13}C NMR spectrum of **2i** in $CDCl_3$ at 100 MHz

^{1}H NMR, ^{13}C NMR Spectra, and HPLC profiles of 3-indolyl furanoids

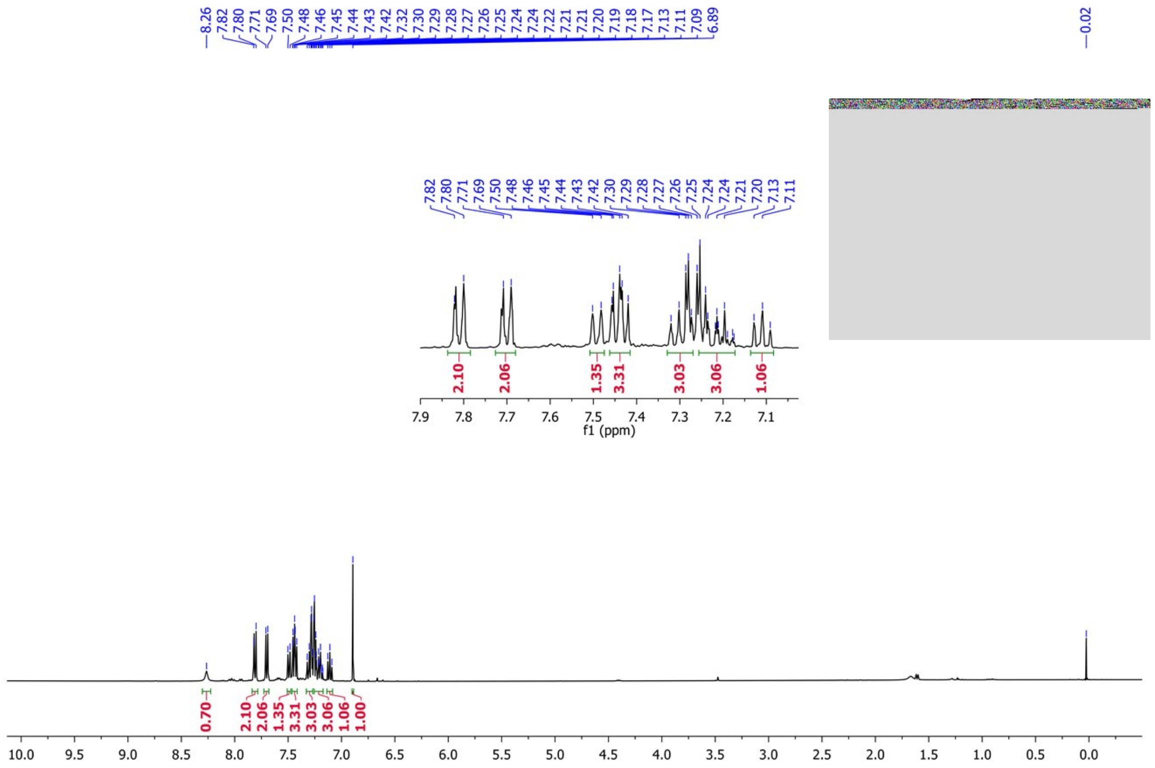

Figure S13: ^{1}H NMR spectrum of **3aa** in $CDCl_3$ at 400 MHz

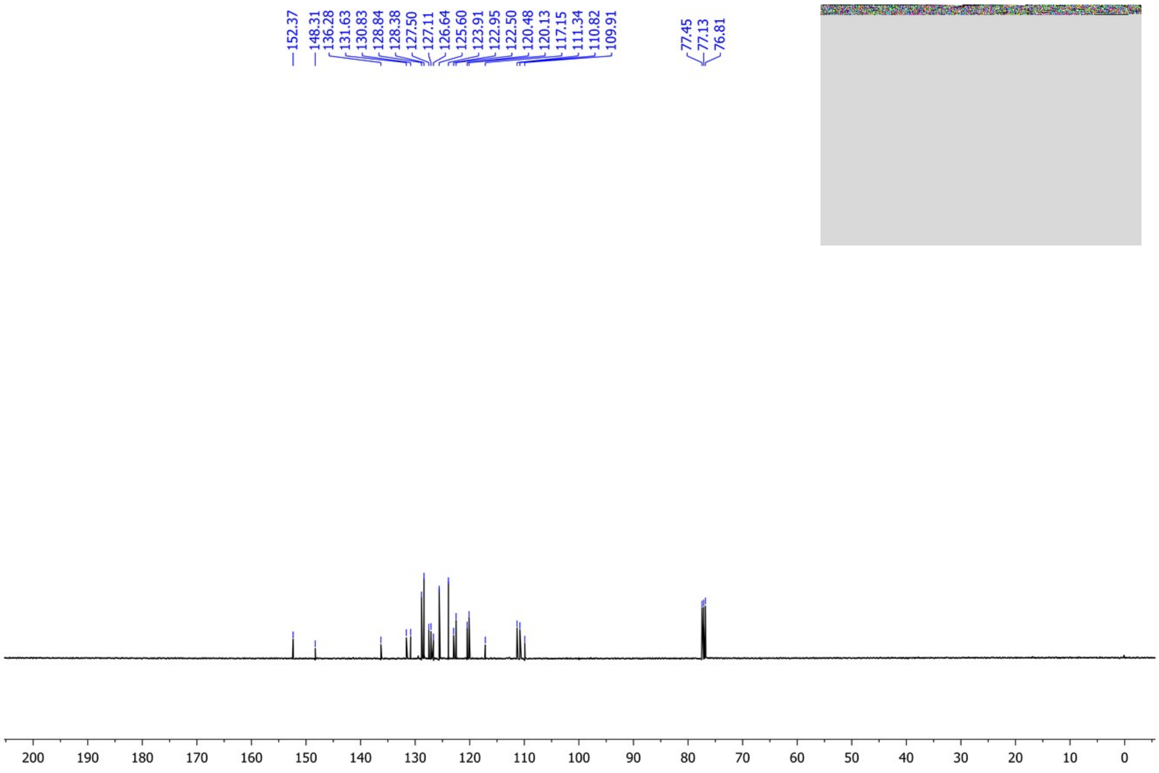

Figure 14: ^{13}C NMR spectrum of **3aa** in $CDCl_3$ at 100 MHz

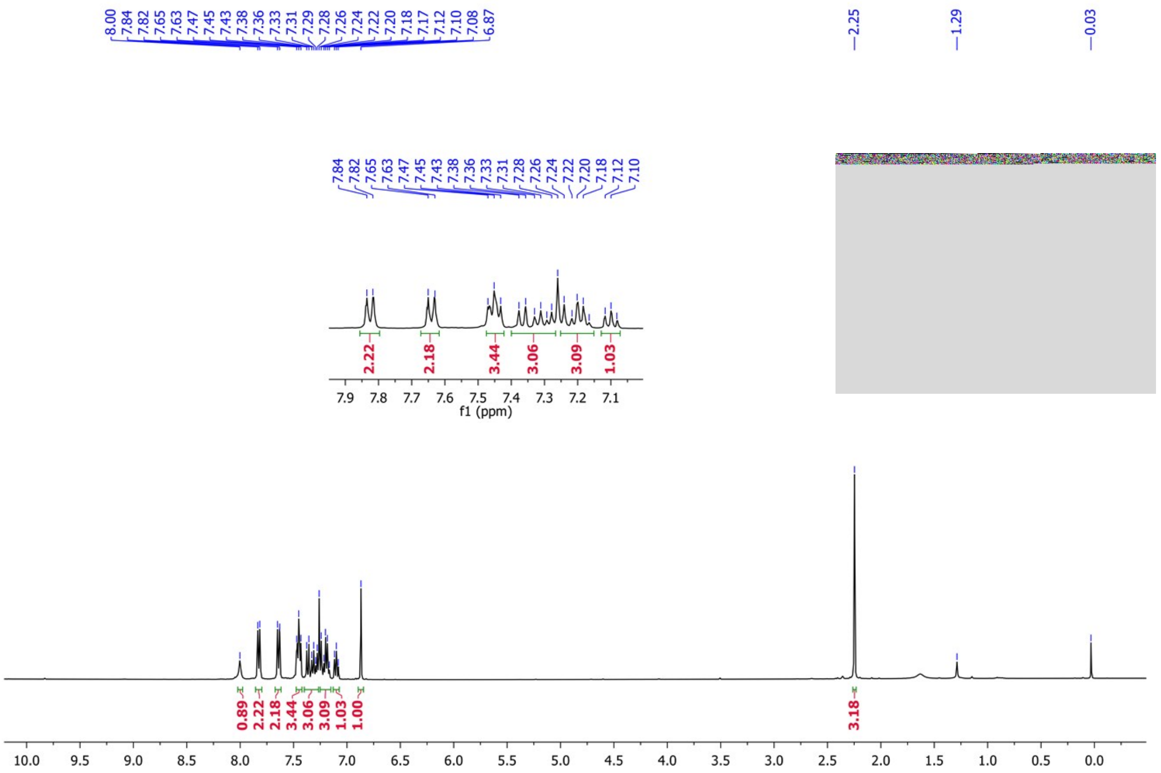

Figure S15: ^{1}H NMR spectrum of **3ba** in $CDCl_3$ at 400 MHz

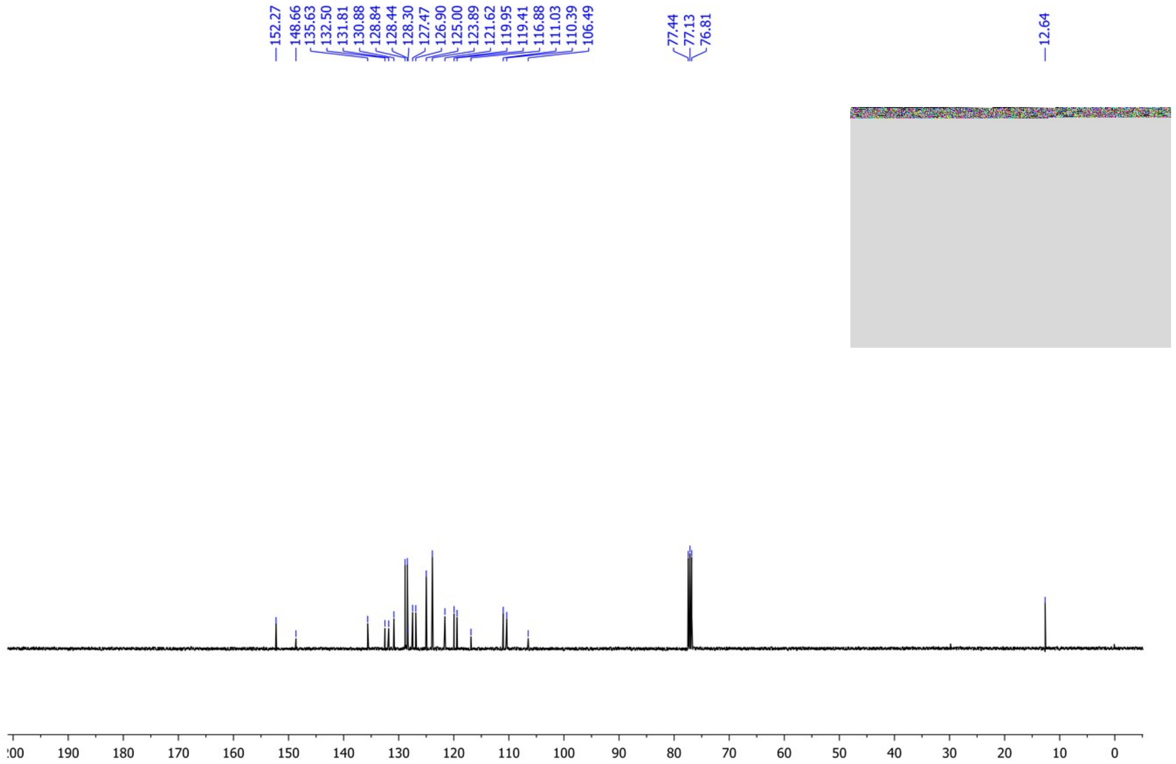

Figure S16: ^{13}C NMR spectrum of **3ba** in $CDCl_3$ at 100 MHz

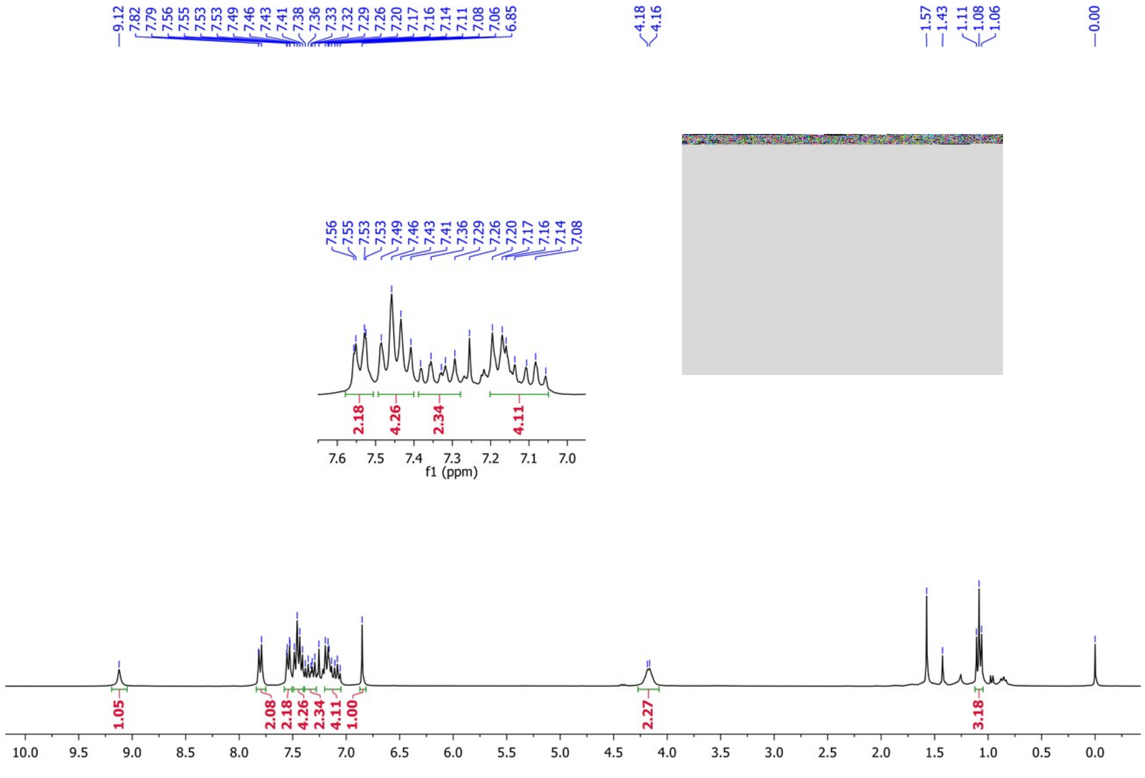

Figure S17: ^{1}H NMR spectrum of **3ca** in $CDCl_3$ at 300 MHz

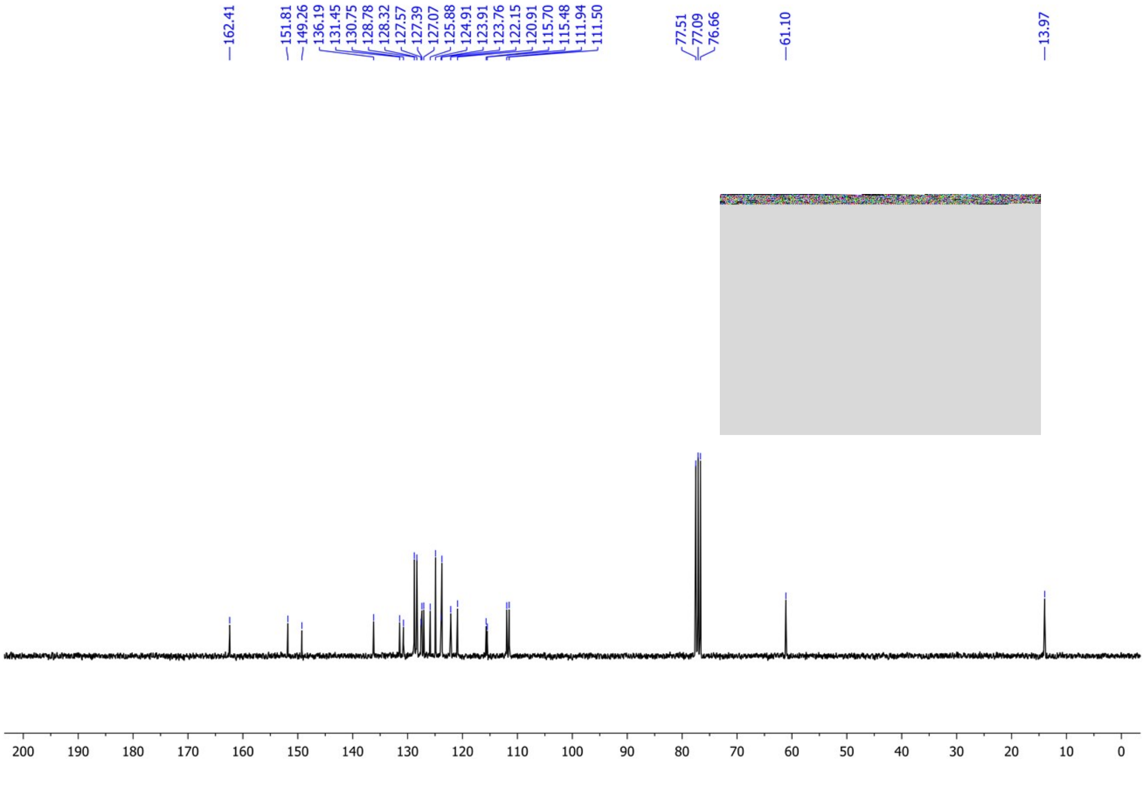

Figure S18: ^{13}C NMR spectrum of **3ca** in $CDCl_3$ at 75 MHz

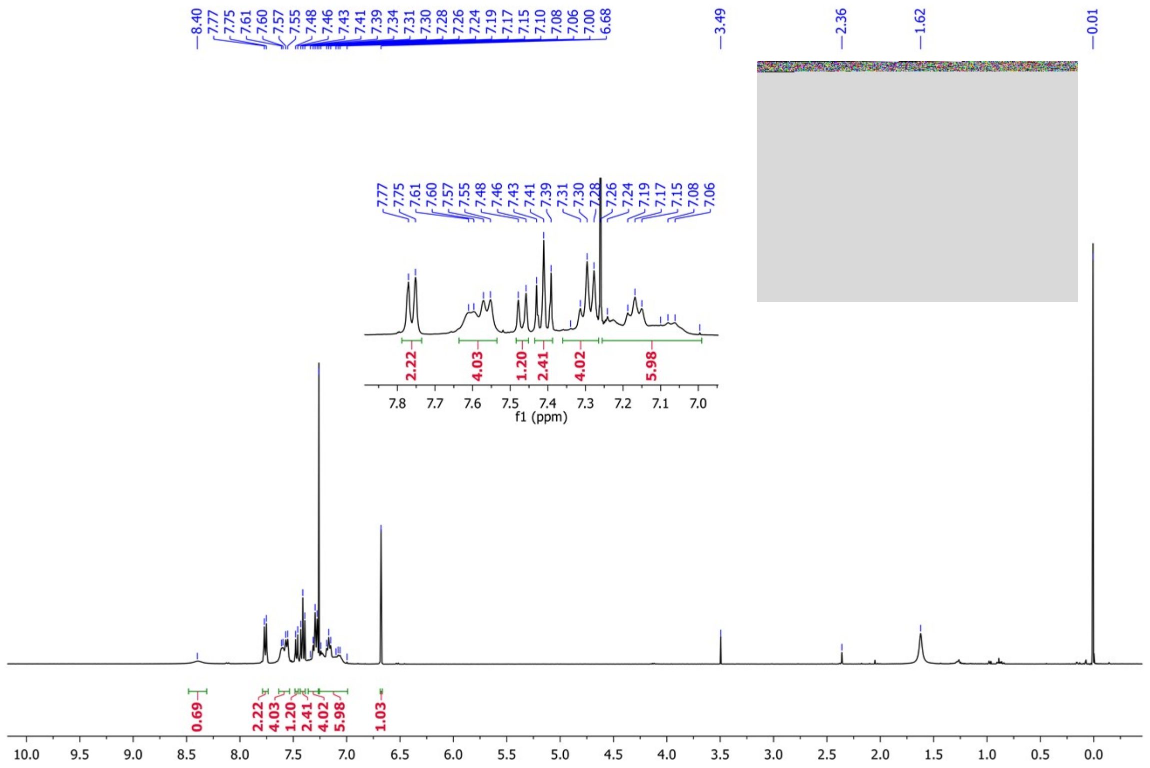

Figure S19: ^{1}H NMR spectrum of **3da** in $CDCl_3$ at 400 MHz

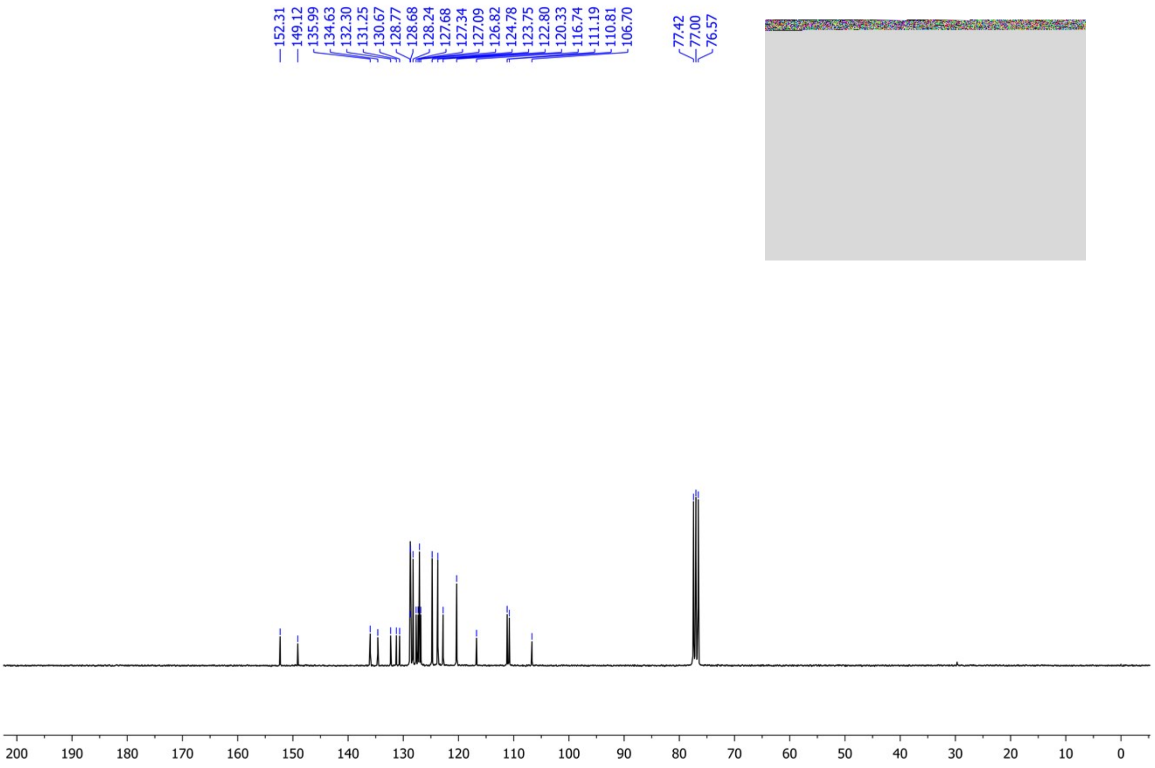

Figure S20: ^{13}C NMR spectrum of **3da** in $CDCl_3$ at 100 MHz

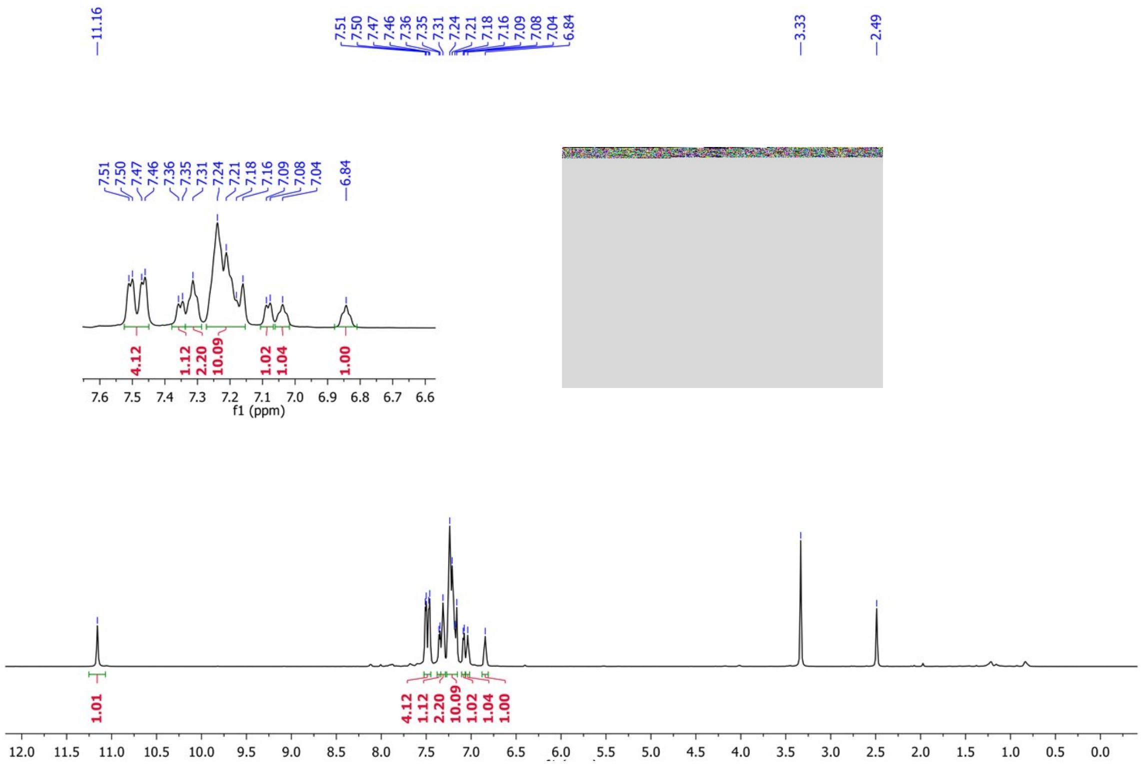

Figure S21: ^{1}H NMR spectrum of **3ab** in DMSO-d_6 at 600 MHz

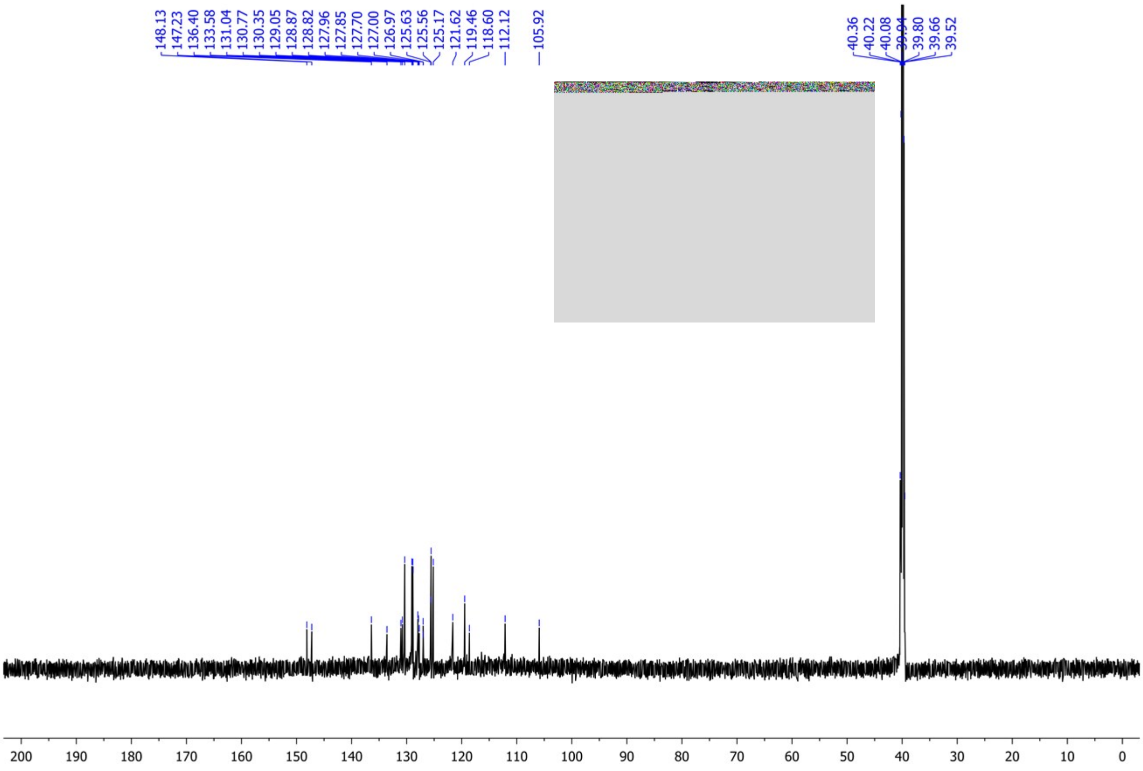

Figure S22: ^{13}C NMR spectrum of **3ab** in DMSO-d_6 at 150 MHz

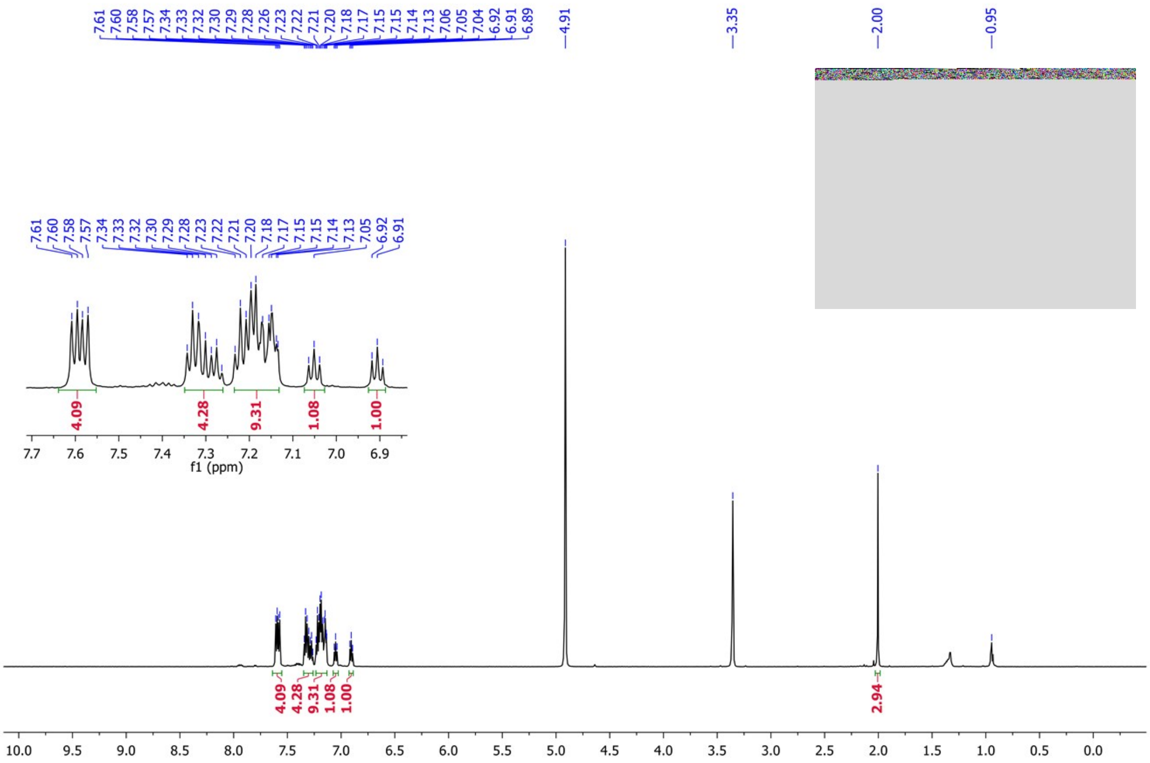

Figure S23: ^{1}H NMR spectrum of **3bb** in CD_3OD at 600 MHz

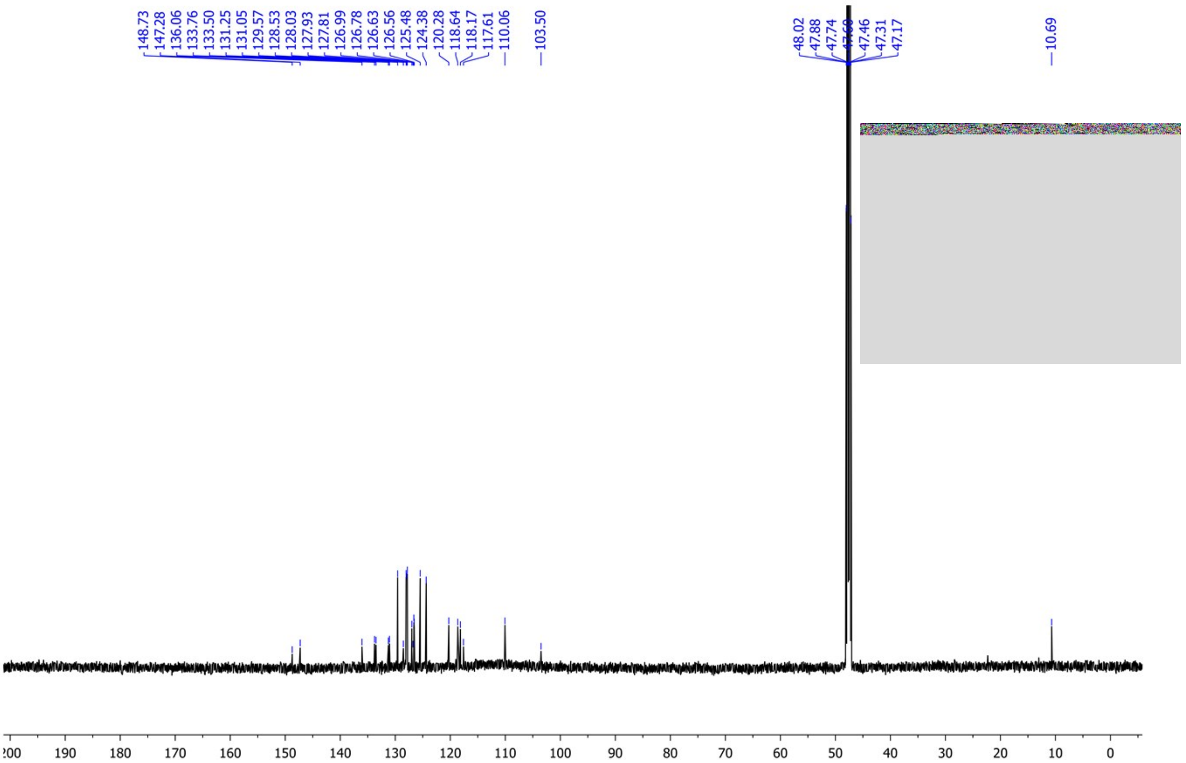

Figure S24: ^{13}C NMR spectrum of **3bb** in CD_3OD at 150 MHz

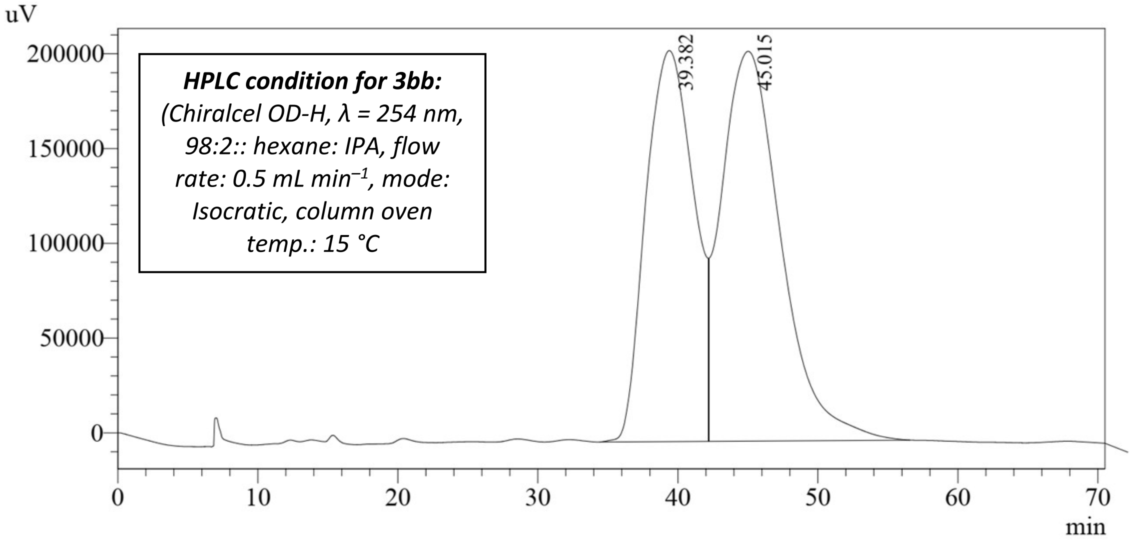

PDA Ch1 254nm 4nm

Peak#	Ret. Time	Area	Height	Area %	Height %
1	39.382	49244694	206290	44.671	50.076
2	45.015	60993752	205662	55.329	49.924
Total		110238446	411952	100.000	100.000

Figure S25: Chiral HPLC profile of **3bb**

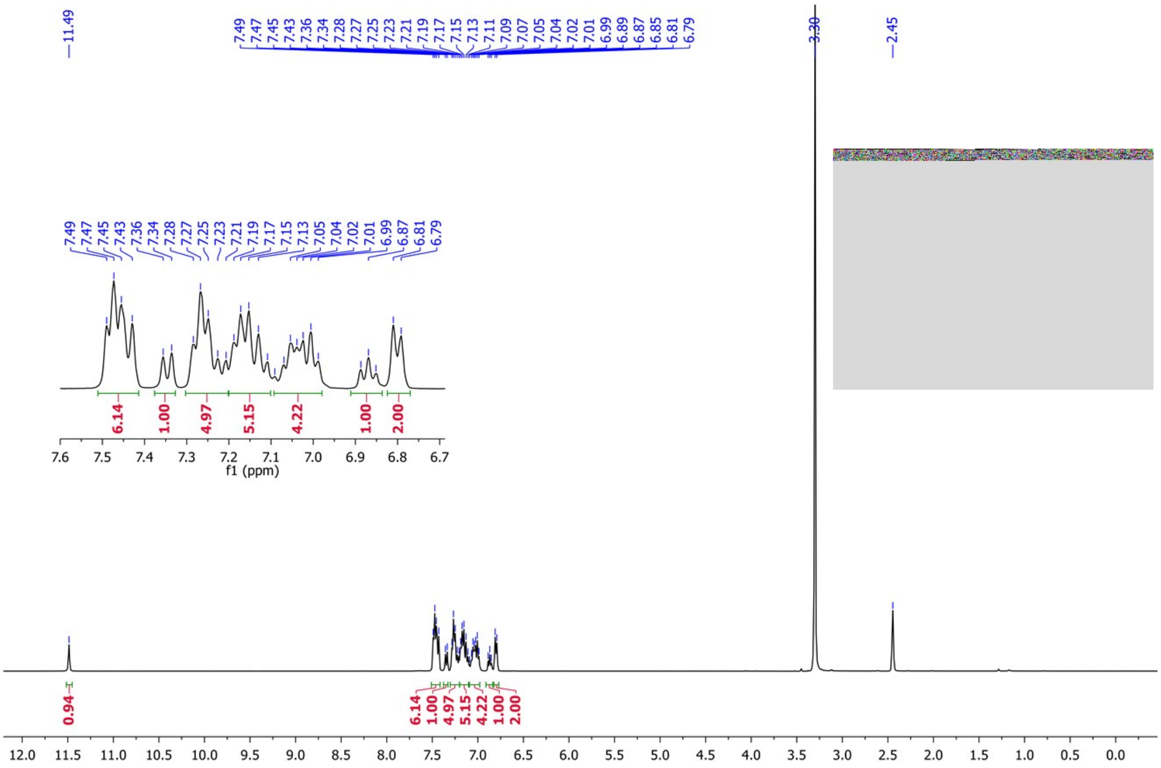

Figure S26: ^{1}H NMR spectrum of **3db** in DMSO-d$_6$ at 400 MHz

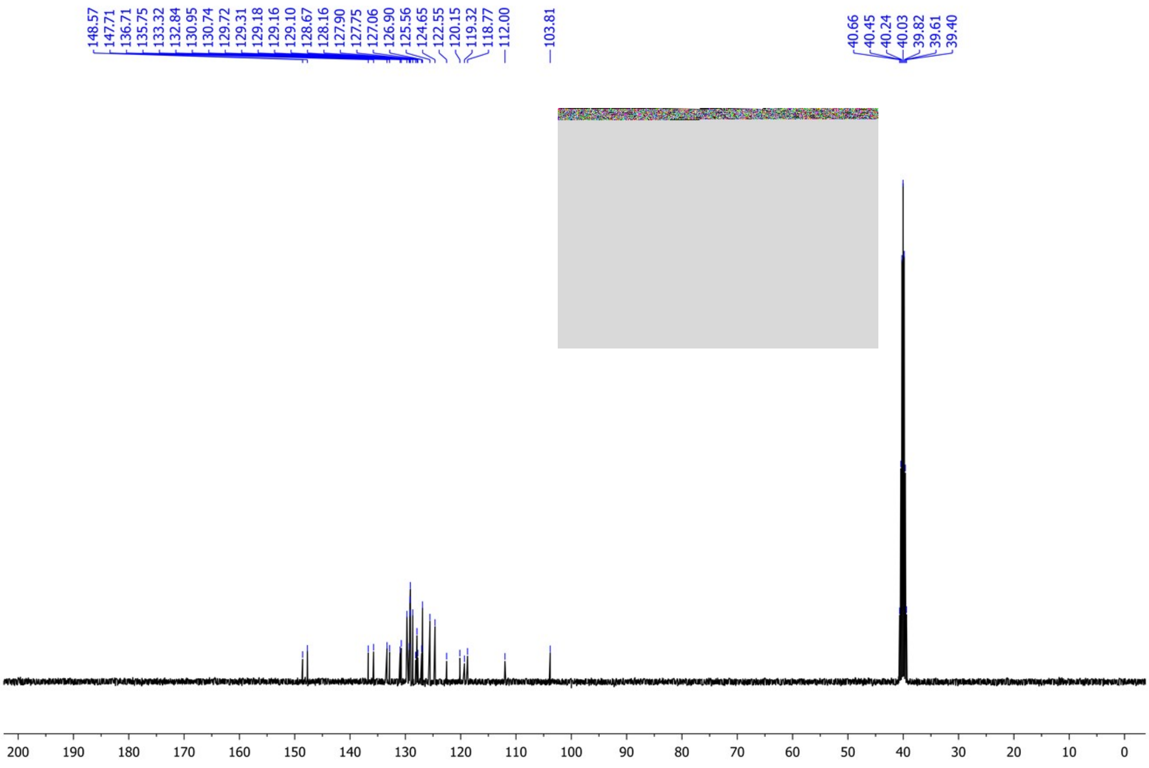

Figure S27: ^{13}C NMR spectrum of **3db** in DMSO-d_6 at 100 MHz

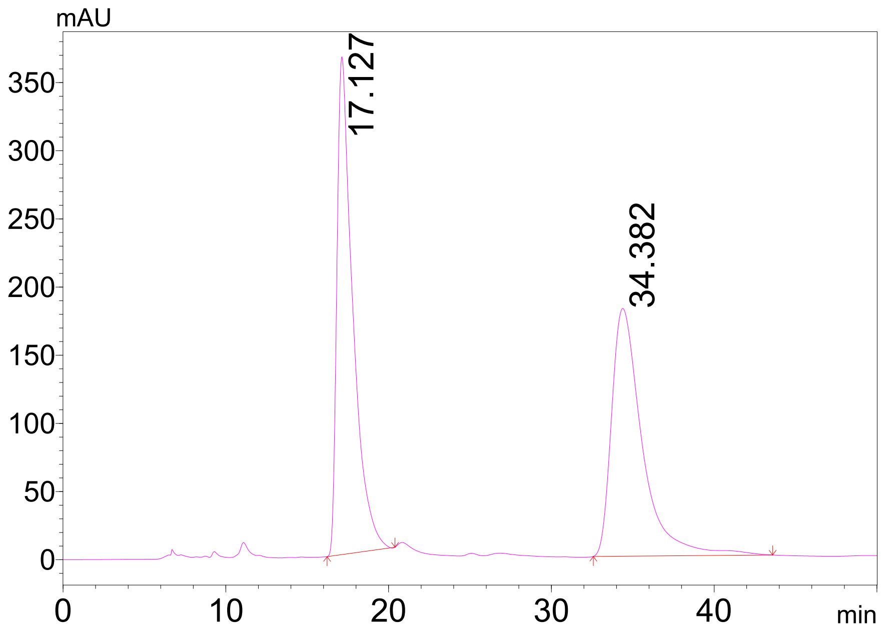

PDA Ch1 254nm 4nm

Peak#	Ret. Time	Area	Height	Area %	Height %
1	17.127	24797912	365029	50.973	66.764
2	34.382	23850946	181720	49.027	33.236
Total		48648858	546749	100.000	100.000

Figure S28: Chiral HPLC profile of **3db**

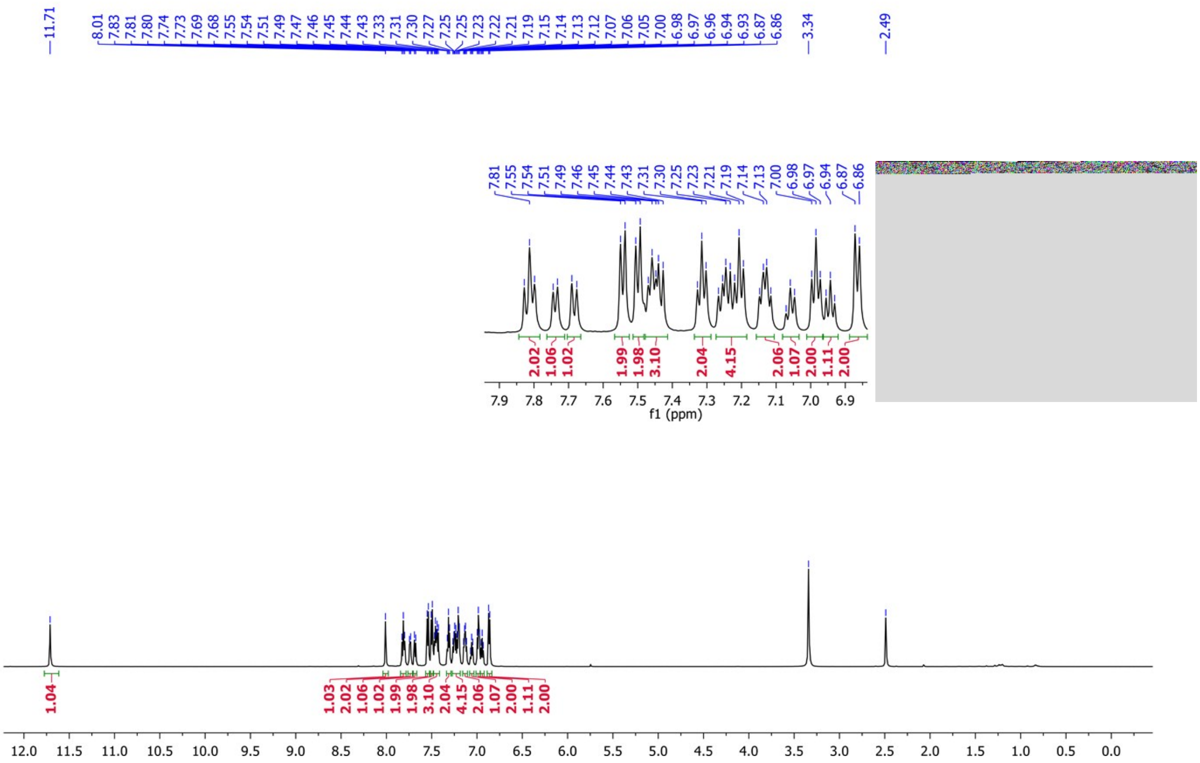

Figure S29: ^{1}H NMR spectrum of **3eb** in DMSO-d6 at 600 MHz

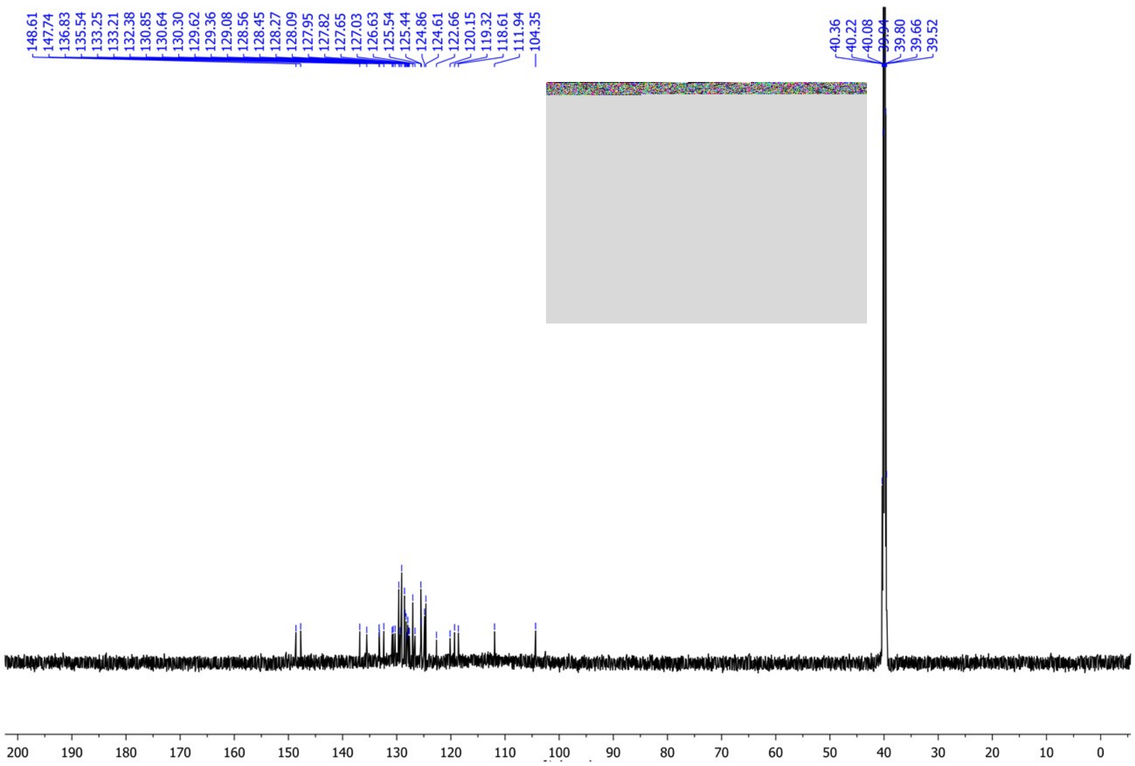

Figure S30: ^{13}C NMR spectrum of **3eb** in DMSO-d6 at 150 MHz

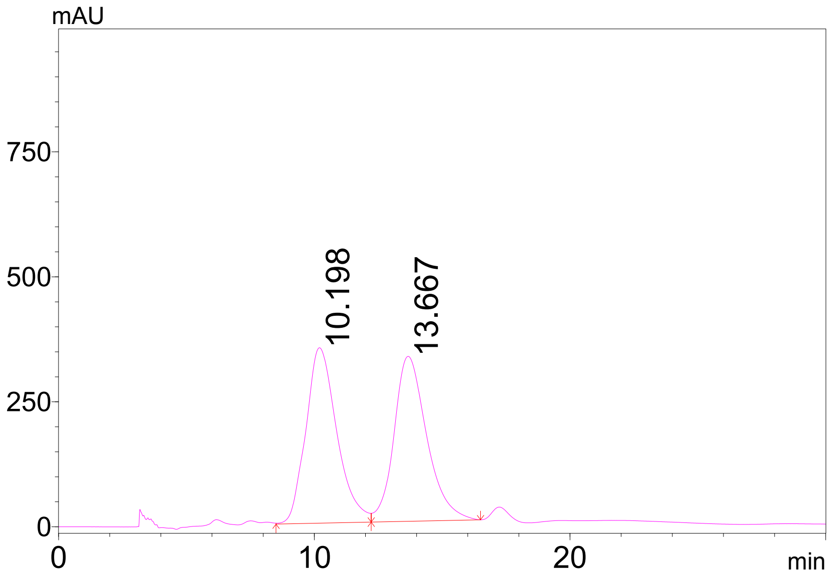

PDA Ch1 265nm 4nm

Peak#	Ret. Time	Area	Height	Area %	Height %
1	10.198	29332947	349536	50.039	51.478
2	13.667	29286653	329468	49.961	48.522
Total		58619601	679004	100.000	100.000

Figure S31: Chiral HPLC profile of **3eb**

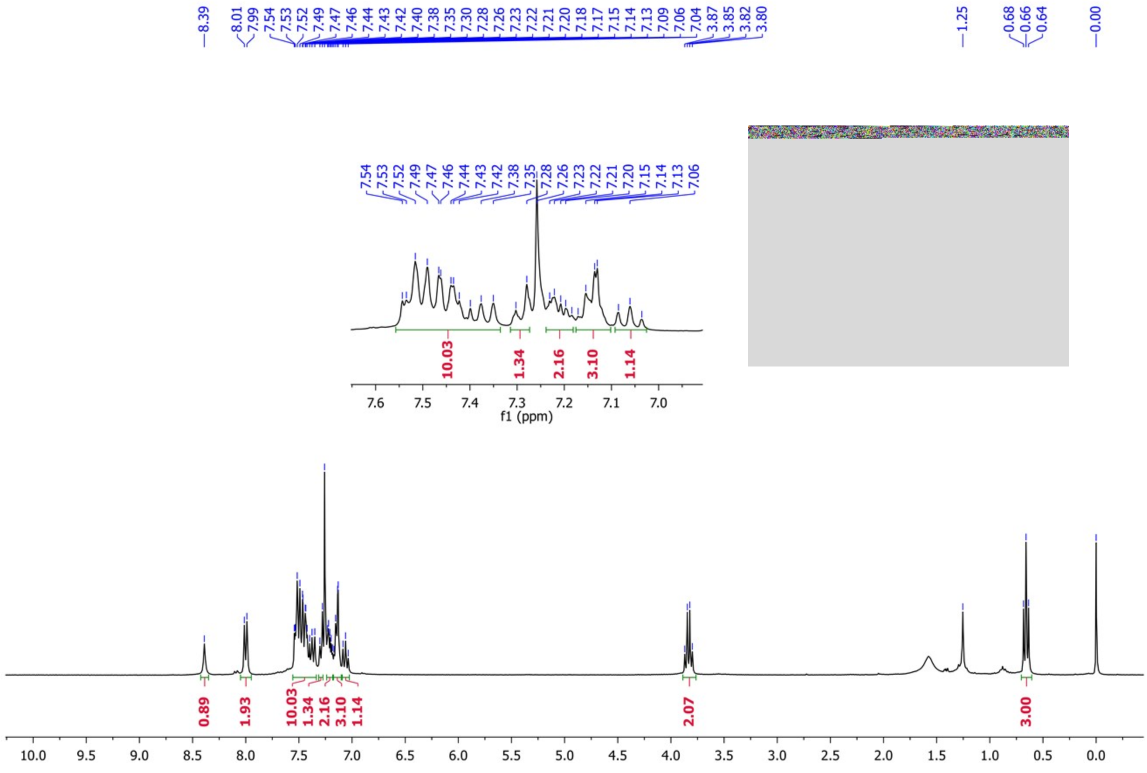

Figure S32: ^{1}H NMR spectrum of **3dc** in $CDCl_3$ at 300 MHz

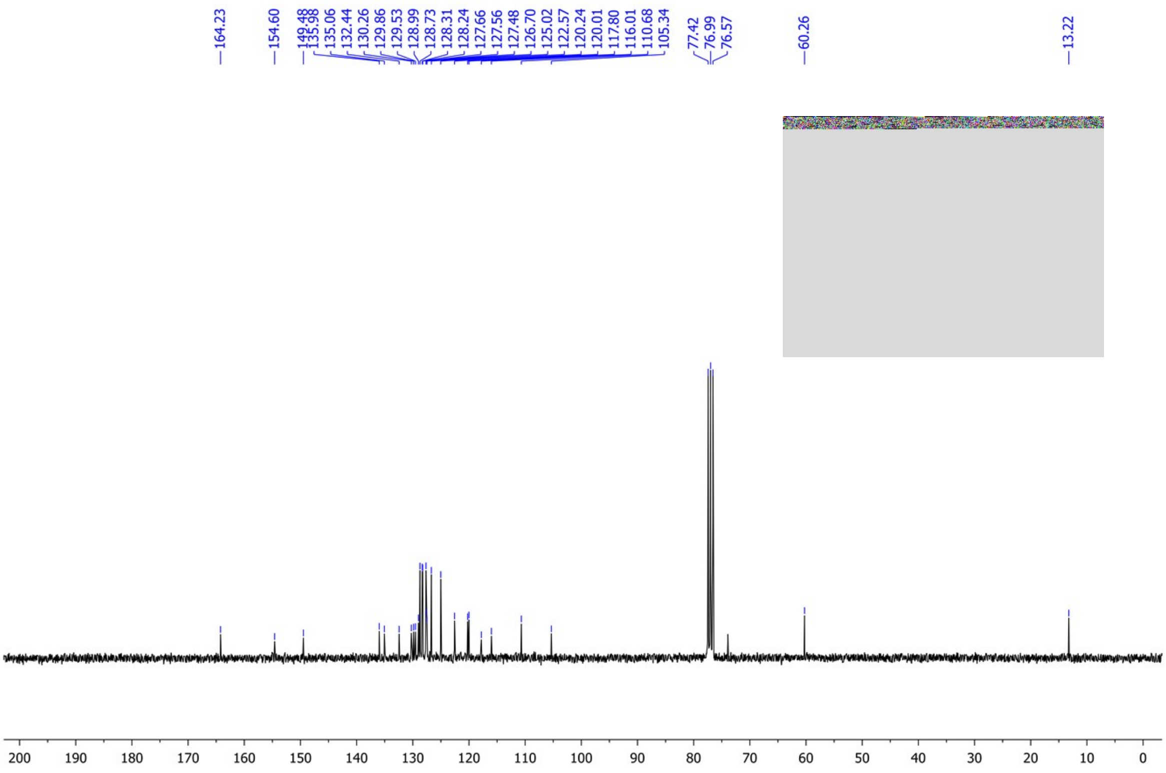

Figure S33: ^{13}C NMR spectrum of **3dc** in $CDCl_3$ at 75 MHz

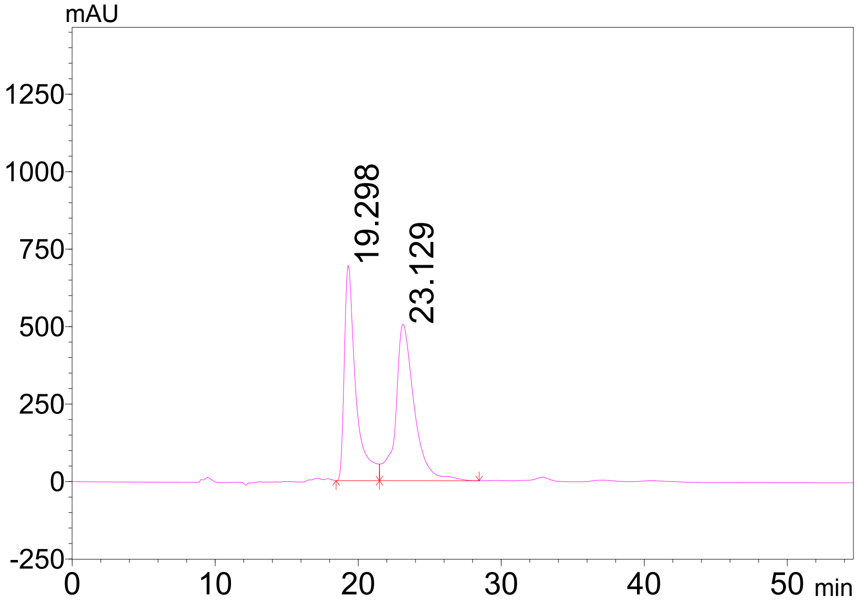

PDA Ch1 254nm 4nm

Peak#	Ret. Time	Area	Height	Area %	Height %
1	19.298	39052426	694630	46.002	57.877
2	23.129	45840619	505558	53.998	42.123
Total		84893045	1200187	100.000	100.000

Figure S34: Chiral HPLC profile of **3dc**

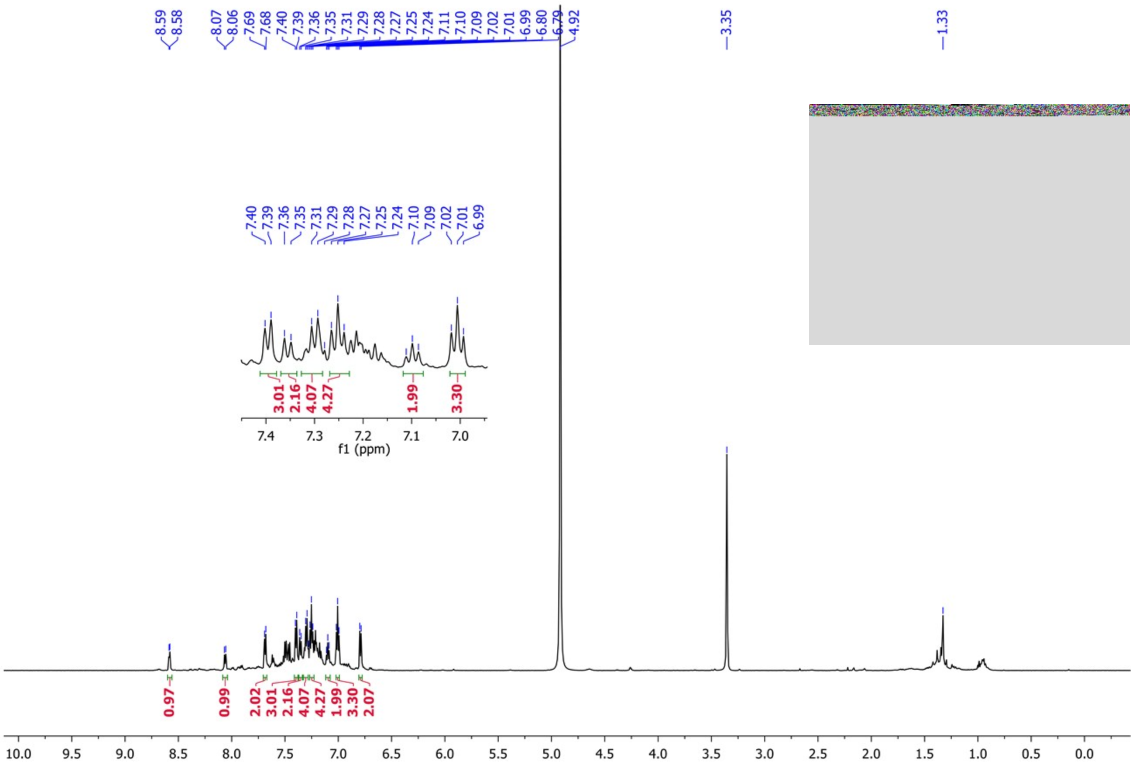

Figure S35: ^{1}H NMR spectrum of **3dd** in CD_3OD at 600 MHz

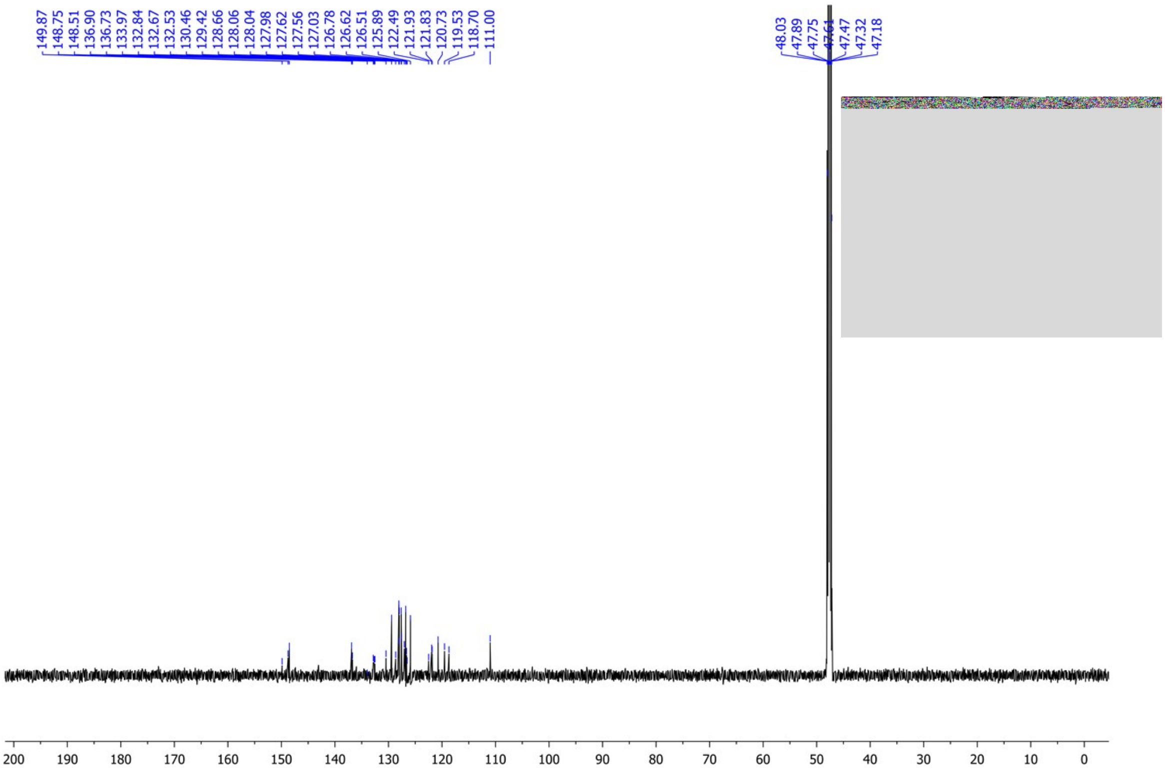

Figure S36: ^{13}C NMR spectrum of **3dd** in CD_3OD at 150 MHz

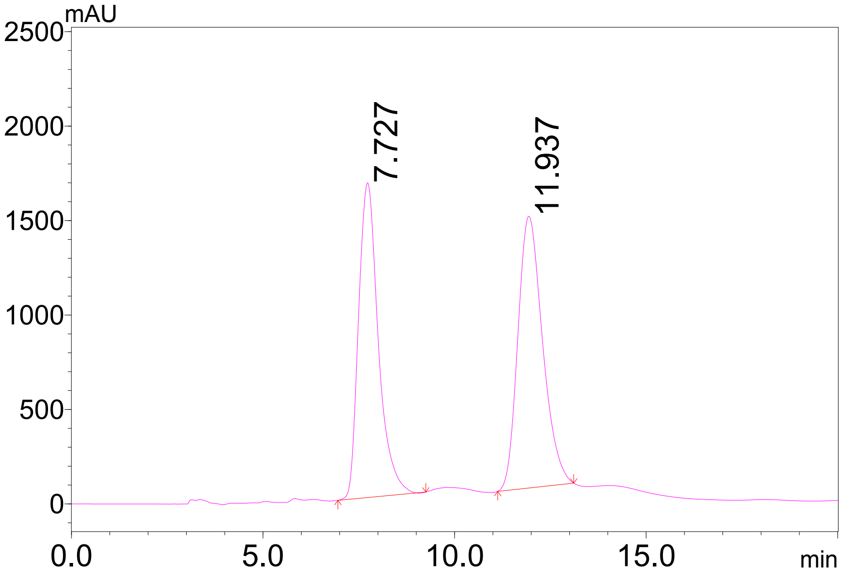

PDA Ch1 254nm 4nm

Peak#	Ret. Time	Area	Height	Area %	Height %
1	7.727	59752360	1666302	48.082	53.669
2	11.937	64520060	1438502	51.918	46.331
Total		124272420	3104804	100.000	100.000

Figure S37: Chiral HPLC profile of **3dd**

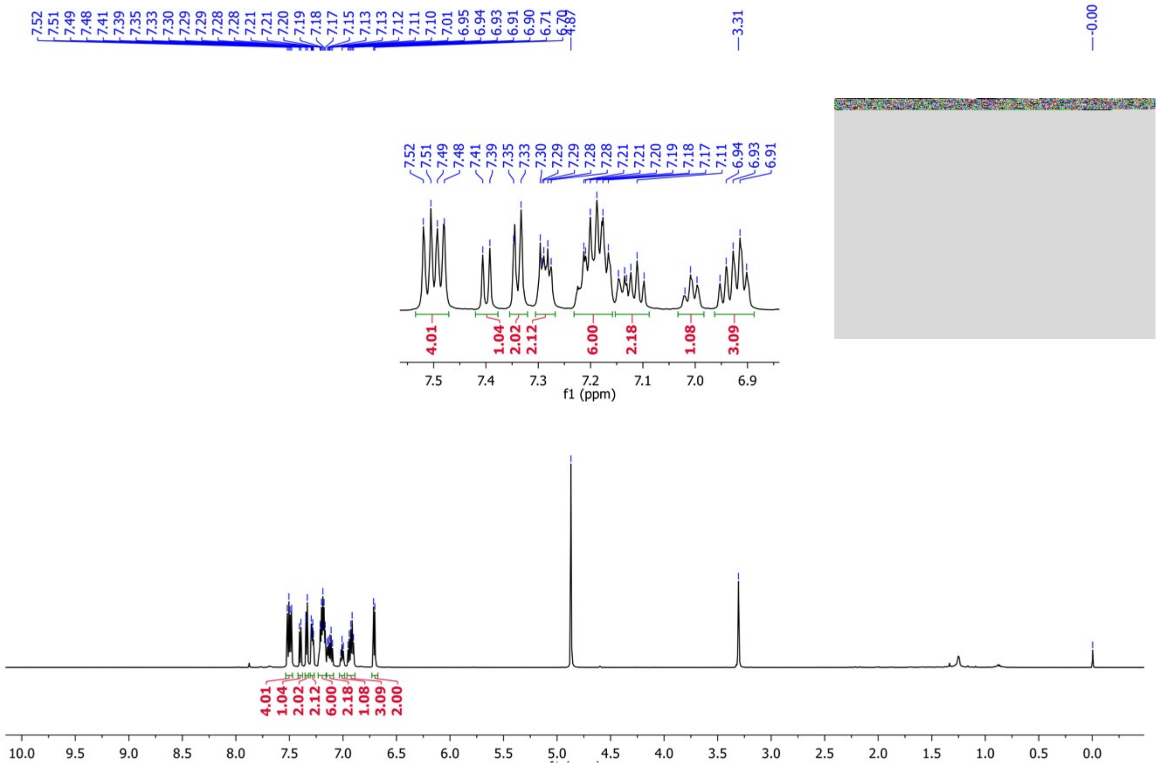

Figure S38: ^{1}H NMR spectrum of **3de** in CD_3OD at 600 MHz

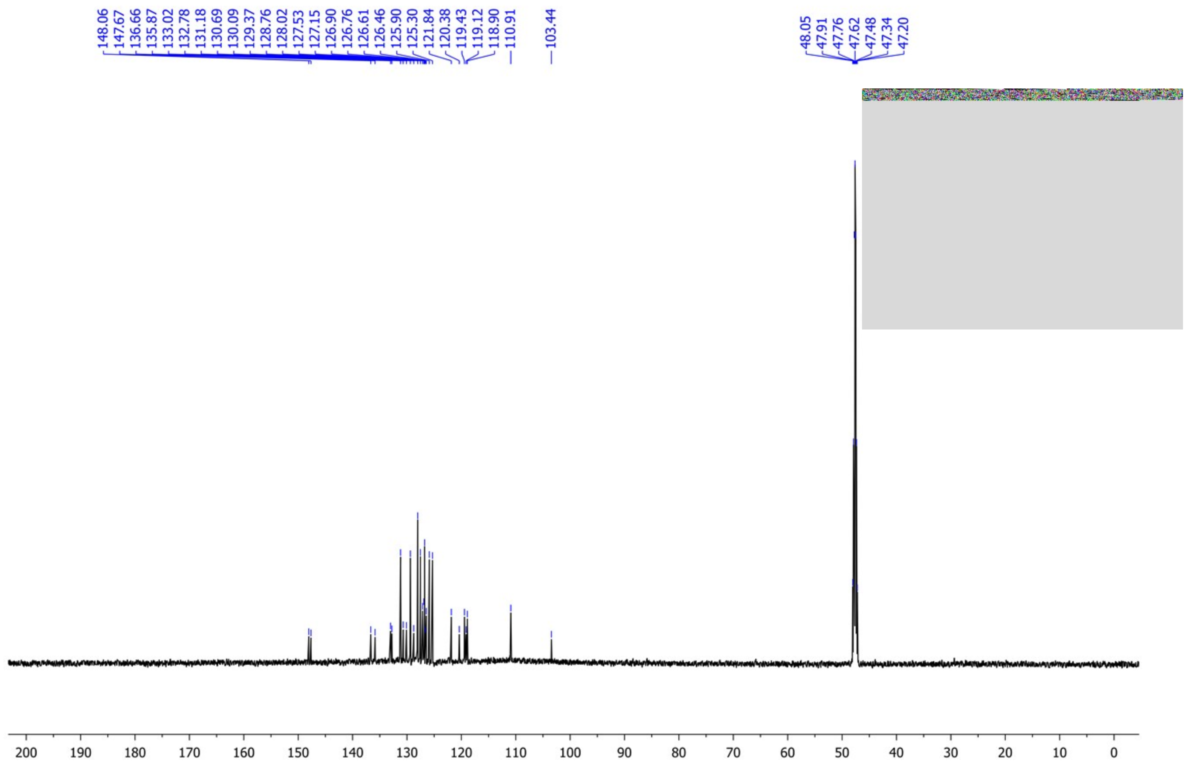

Figure S39: ^{13}C NMR spectrum of **3de** in CD_3OD at 150 MHz

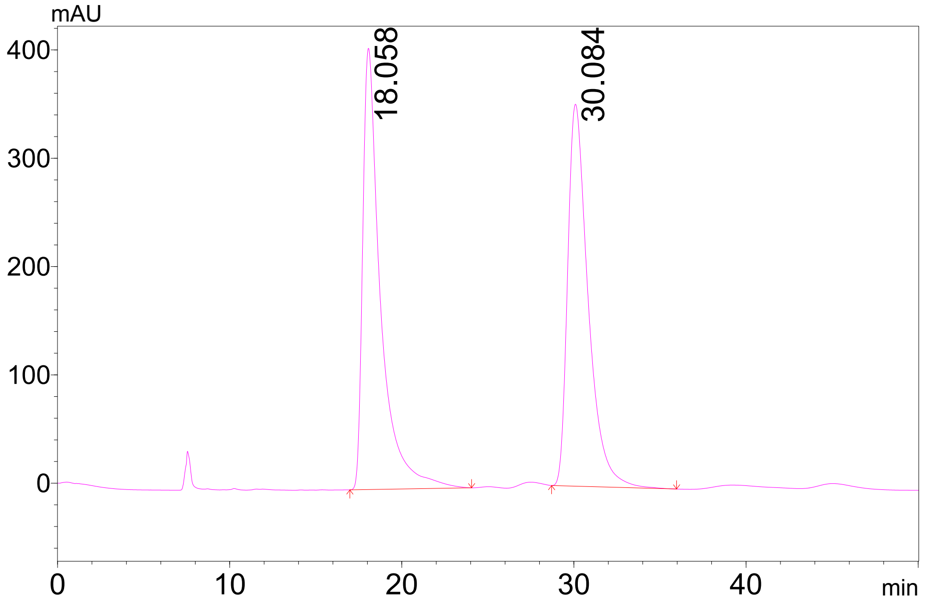

PDA Ch1 254nm 4nm

Peak#	Ret. Time	Area	Height	Area %	Height %
1	18.058	29455121	407340	50.240	53.594
2	30.084	29173750	352702	49.760	46.406
Total		58628871	760042	100.000	100.000

Figure S40: Chiral HPLC profile of **3de**

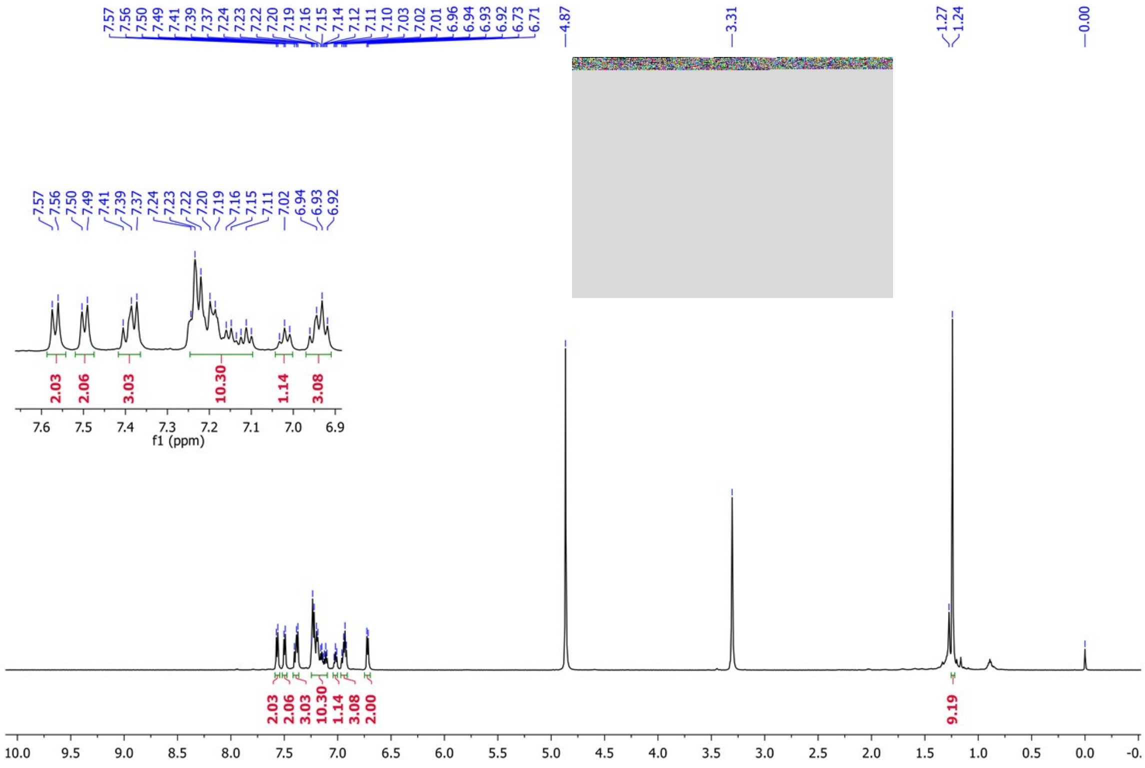

Figure S41: ^{1}H NMR spectrum of **3df** in CD_3OD at 600 MHz

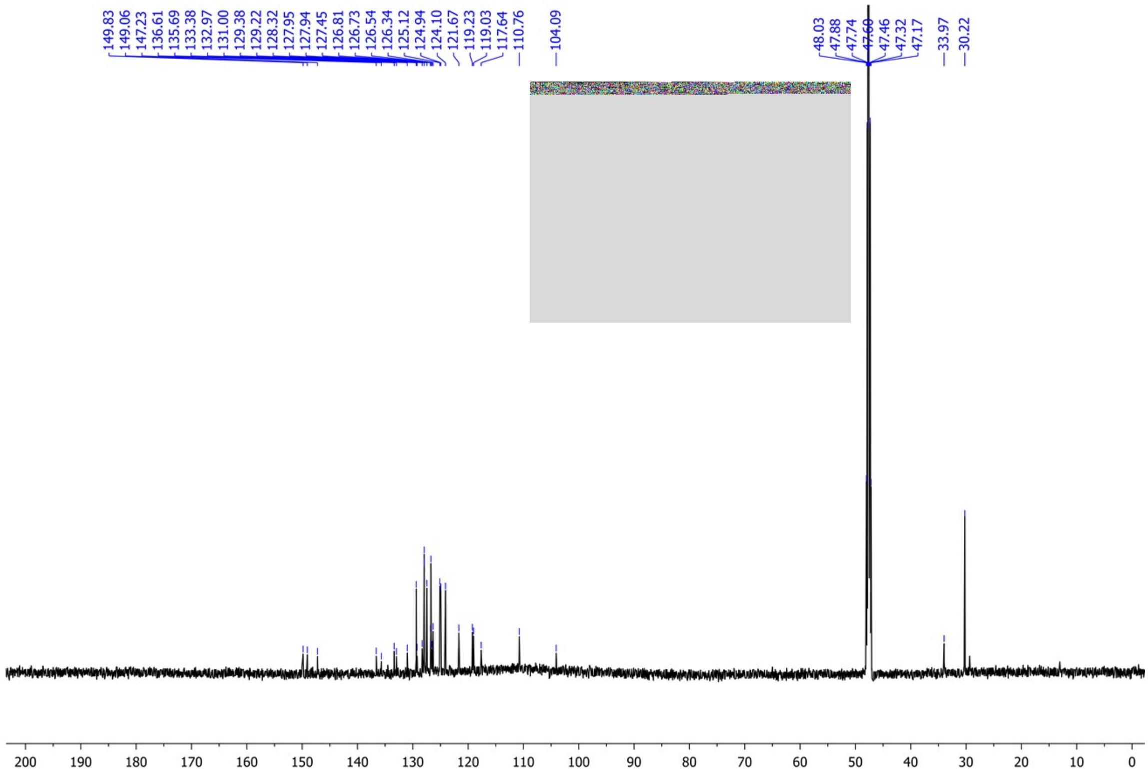

Figure S42: ^{13}C NMR spectrum of **3df** in CD_3OD at 150 MHz

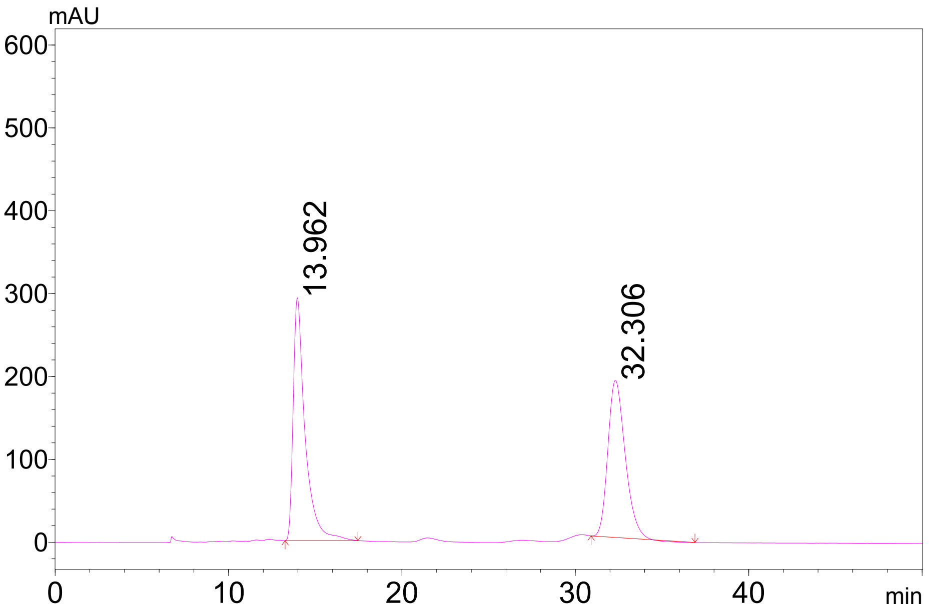

PDA Ch1 254nm 4nm

Peak#	Ret. Time	Area	Height	Area %	Height %
1	13.962	14009342	292878	51.634	60.703
2	32.306	13122826	189598	48.366	39.297
Total		27132168	482476	100.000	100.000

Figure S43: Chiral HPLC profile of **3df**

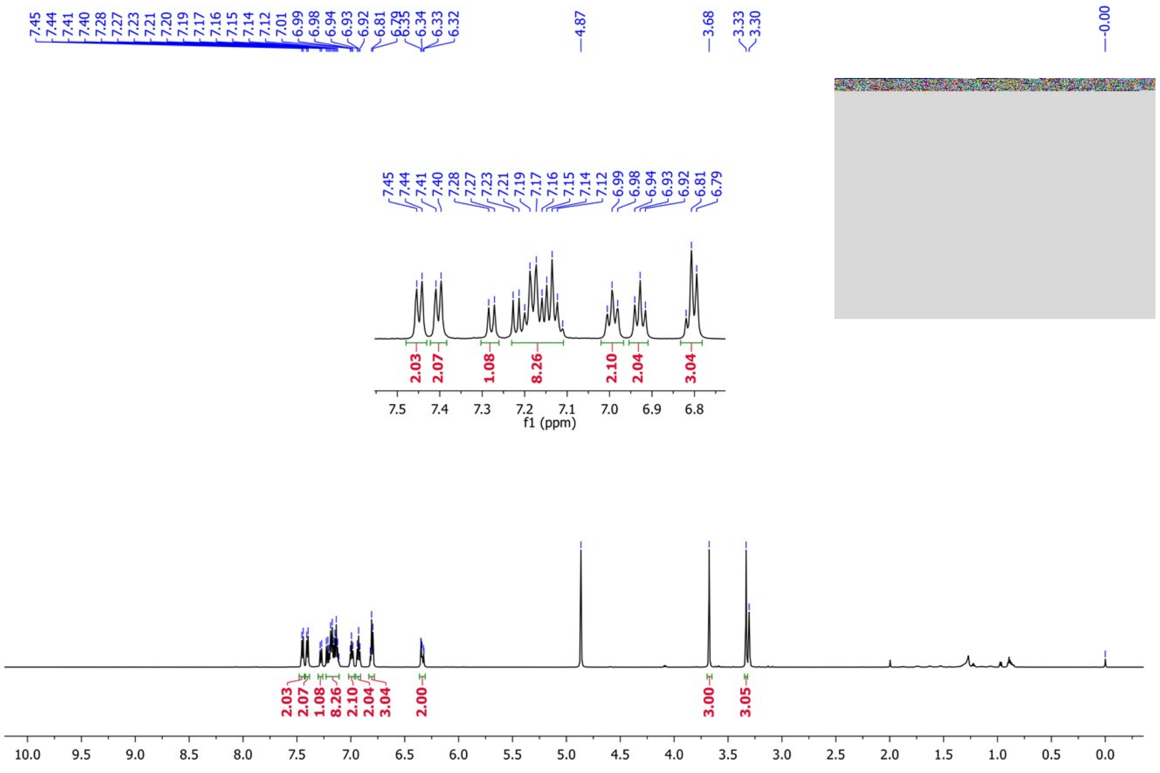

Figure S44: ^{1}H NMR spectrum of **3dg** in CD_3OD at 600 MHz

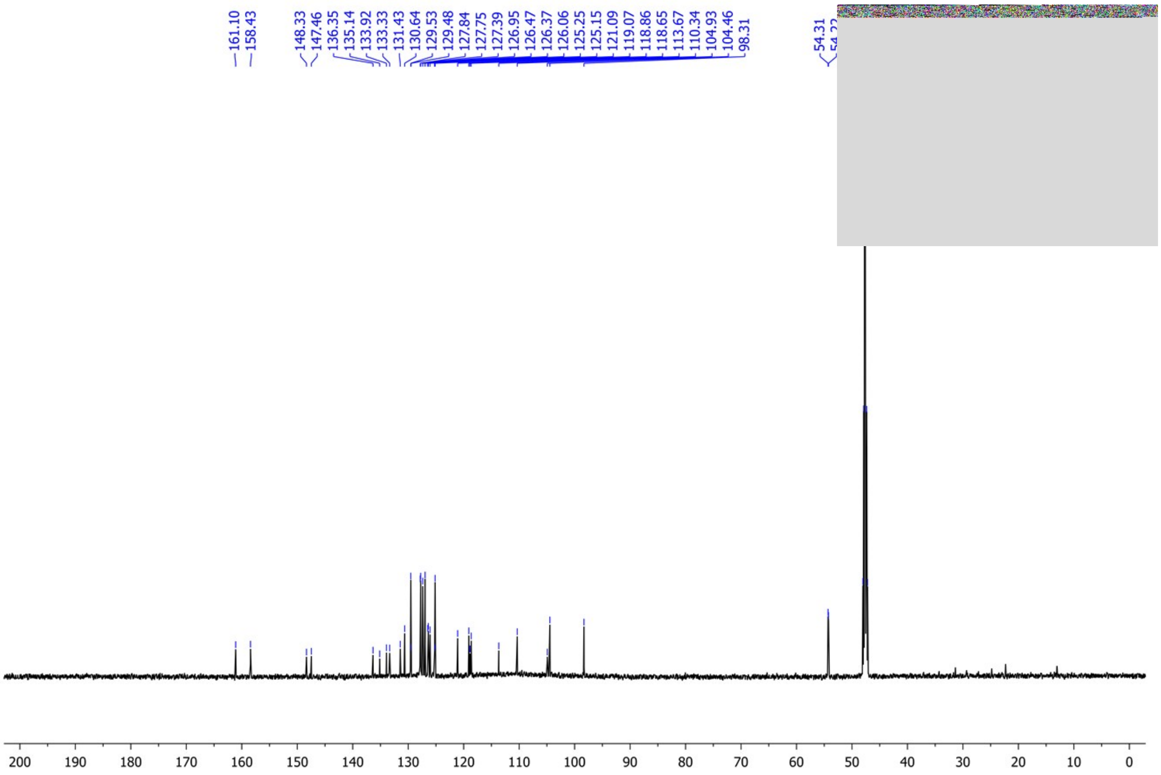

Figure S45: ^{13}C NMR spectrum of **3dg** in CD_3OD at 150 MHz

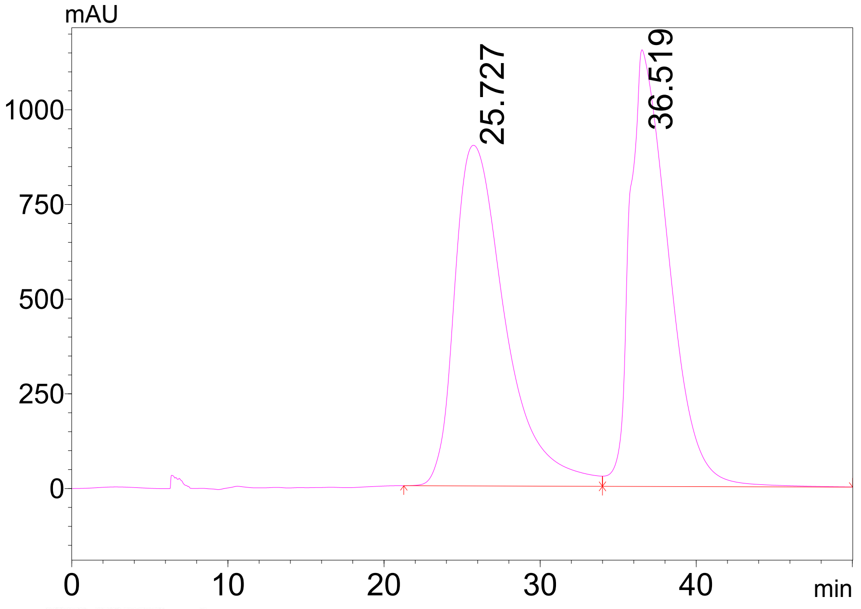

PDA Ch1 254nm 4nm

Peak#	Ret. Time	Area	Height	Area %	Height %
1	25.727	204685681	899427	50.547	43.825
2	36.519	200253396	1152889	49.453	56.175
Total		404939077	2052316	100.000	100.000

Figure S46: Chiral HPLC profile of **3dg**

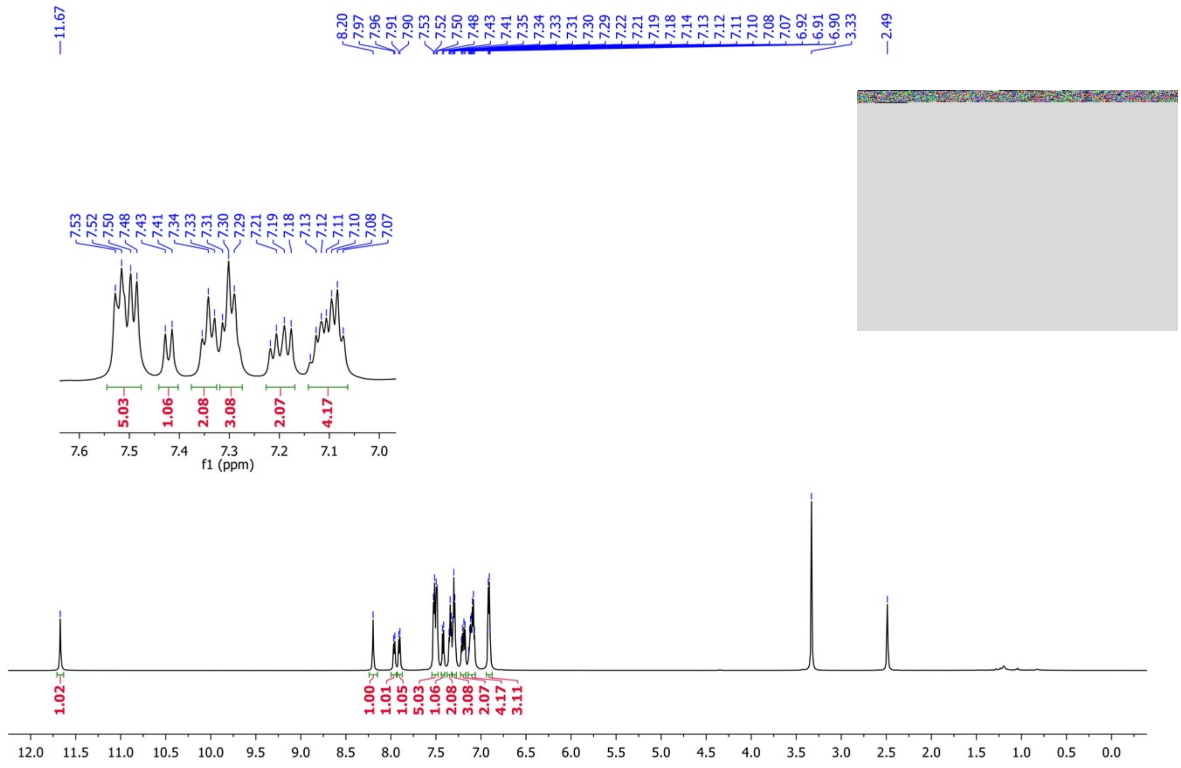

Figure S47: ^{1}H NMR spectrum of **3dh** in DMSO-d6 at 600 MHz

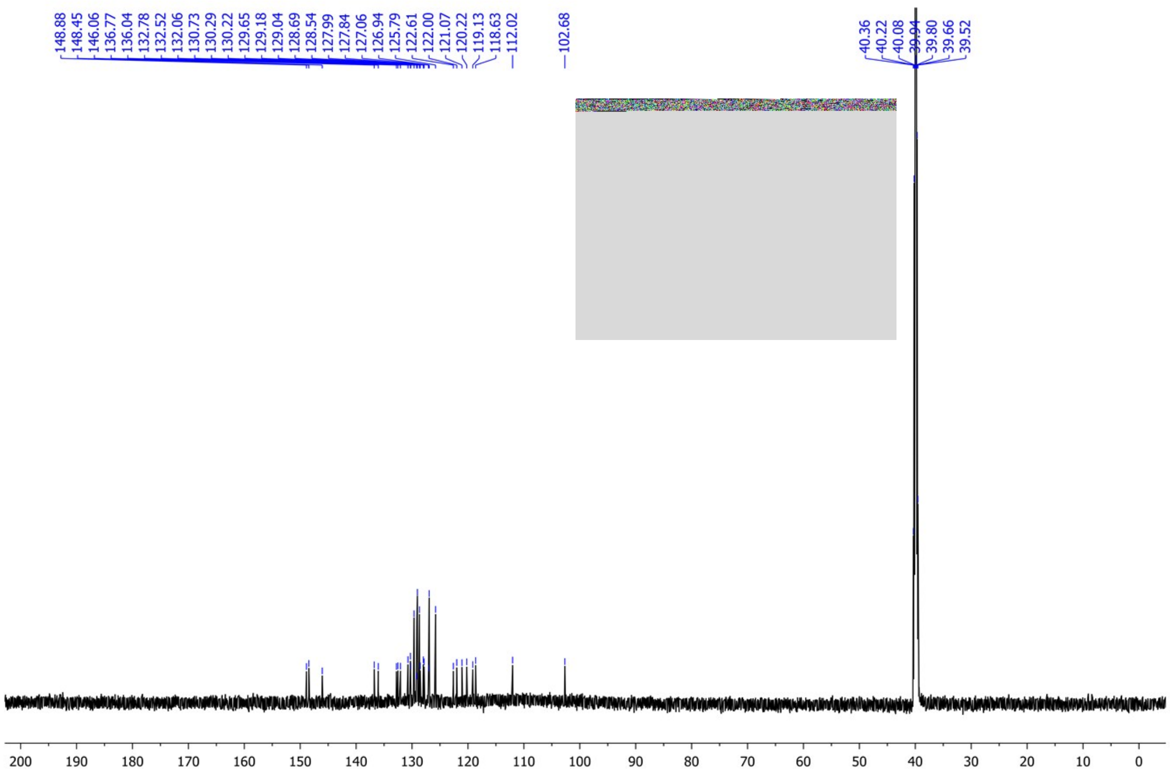

Figure S48: ^{13}C NMR spectrum of **3dh** in DMSO-d6 MHz

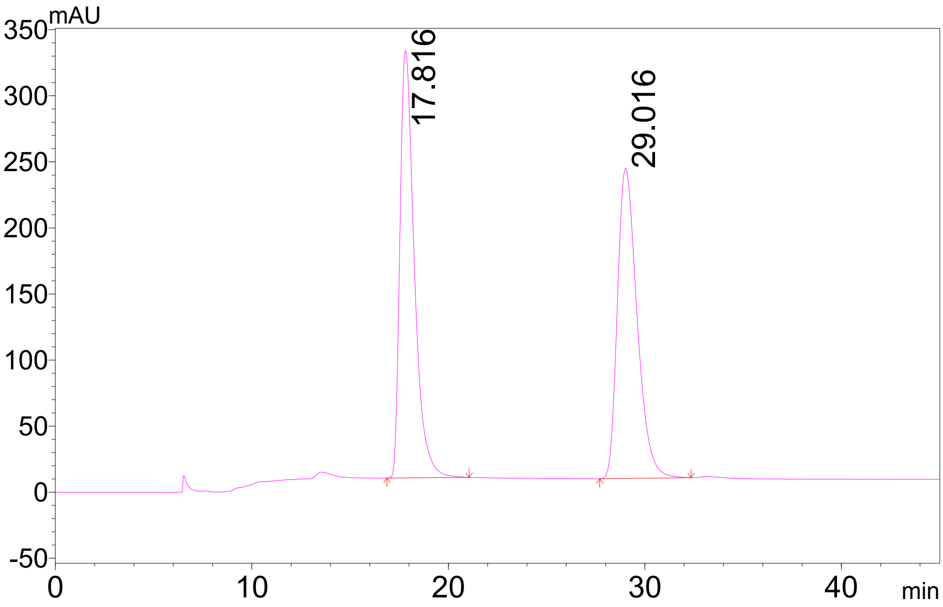

PDA Ch1 254nm 4nm

Peak#	Ret. Time	Area	Height	Area %	Height %
1	17.816	16449372	323471	50.240	57.957
2	29.016	16292338	234651	49.760	42.043
Total		32741710	558122	100.000	100.000

Figure S49: Chiral HPLC profile of **3dh**

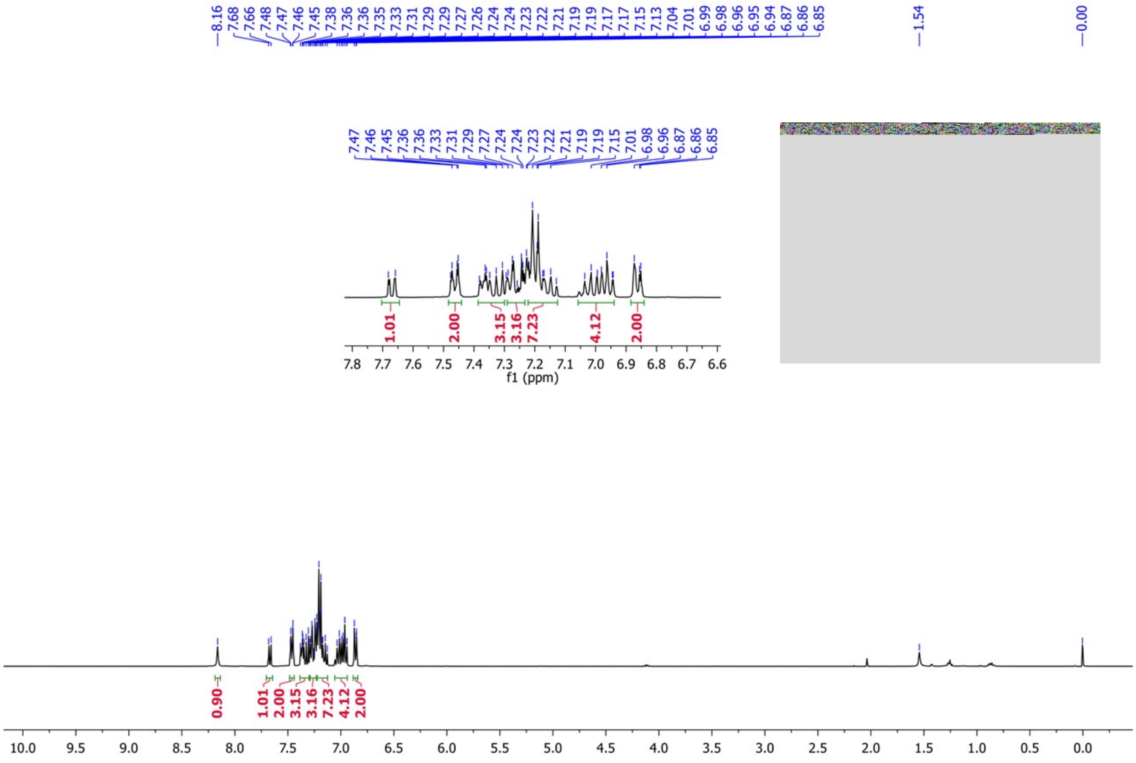

Figure S50: ^{1}H NMR spectrum of **3di** in $CDCl_3$ at 600 MHz

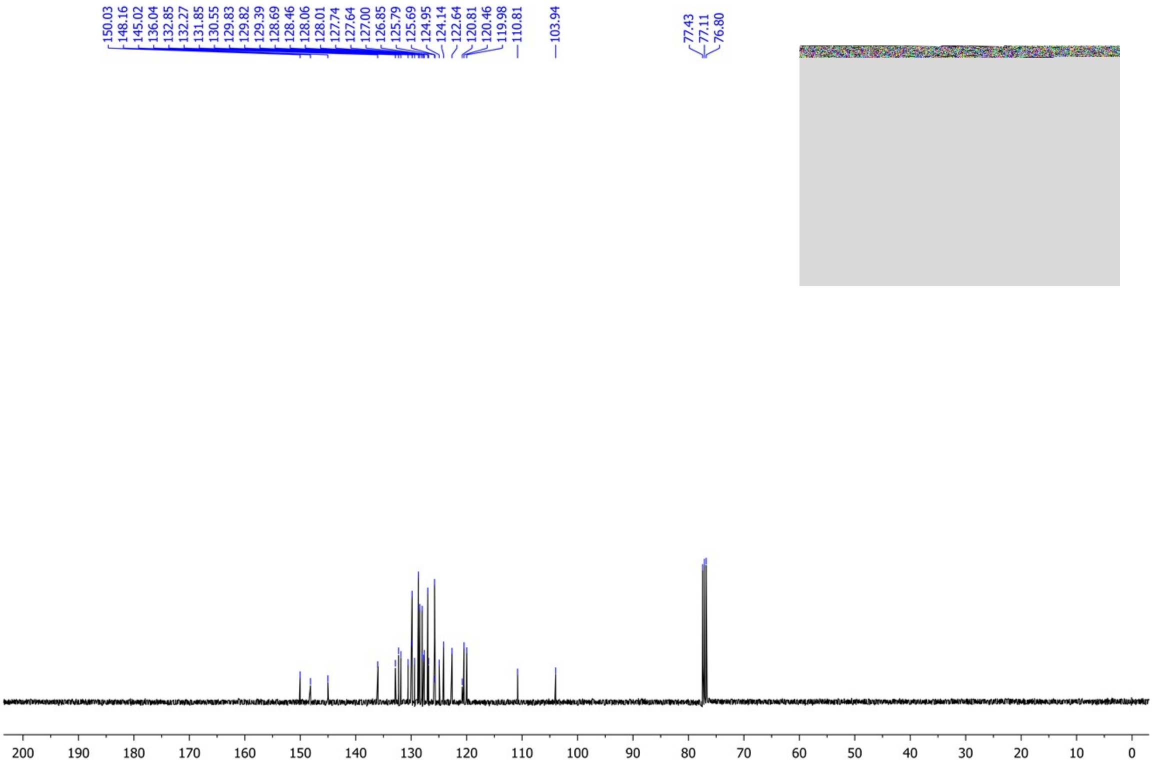

Figure S51: ^{13}C NMR spectrum of **3di** in $CDCl_3$ at 150 MHz

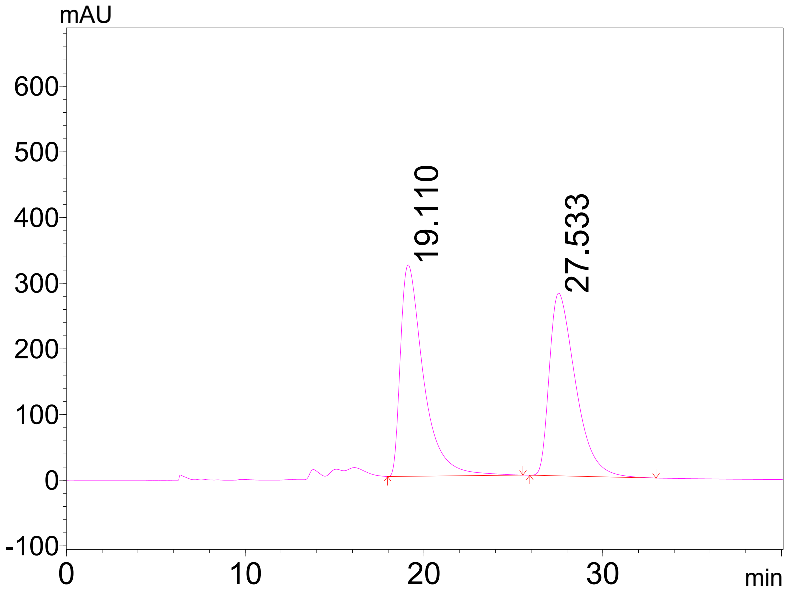

PDA Ch1 254nm 4nm

Peak#	Ret. Time	Area	Height	Area %	Height %
1	19.110	28901689	322000	50.505	53.660
2	27.533	28323582	278079	49.495	46.340
Total		57225271	600079	100.000	100.000

Figure S52: Chiral HPLC profile of **3di**

Section D: HPLC chromatograms of 3db, 3dg, 3dh and 3di

Representative procedure of HPLC analysis for 3db [4,6]: 1.0 mg of (*S*)-**3db** was isolated through chiral HPLC and evaporated in vacuo. The enantiomerically enriched analogue ((*S*)-**3db**) was then dissolved in 1.0 mL ethanol and placed in a sealed 2-dram vial to prepare 1.03 mM ethanolic solution. The mother solution was fractionated into ten glass vials containing 100 µL solution of (*S*)-**3db** fitted with air tight 2-dram vial and kept inside a preheated incubator at 353 K. The same protocol was followed while studied at 333 K and 300 K. For those, samples were kept inside the preheated incubator at a fixed temperature of 333 K and 300 K respectively. Each sample was taken out from the incubator at indicated time interval and stored at 273 K (to immediately stop the further racemization process). Individual samples were then straight away subjected to chiral HPLC analysis by using following condition:

Column: CHIRALCEL OD-H (4.6 mm x 250.0 mm x 5.0 µm)
Solvent: hexane: ethanol (95: 5)
Flow rate: 0.5 mL/min
Mode: Isocratic
Column oven: 298 K
Sample conc.: 1.0 mg/mL ethanol
Injected volume: 5 µL

[a] Racemization kinetics study of (*S*)-3db at 353 K

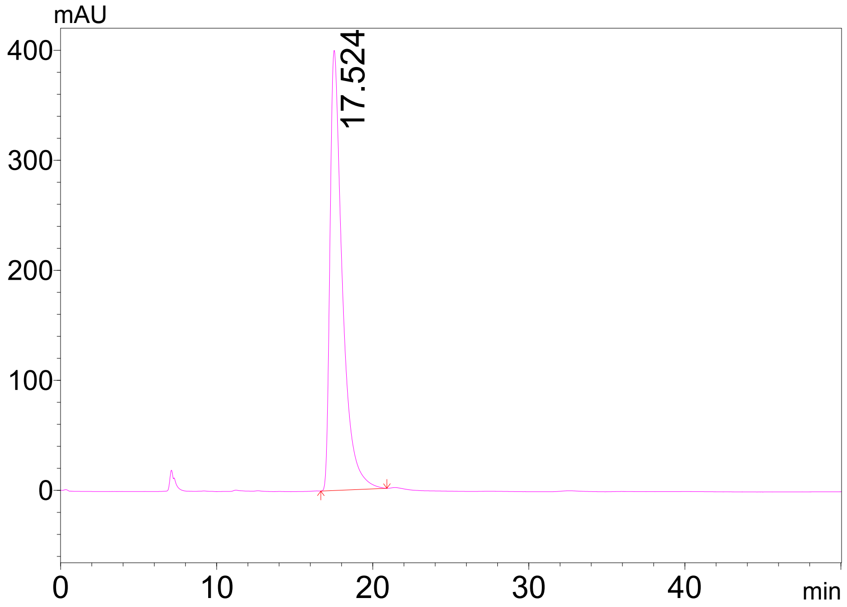

PDA Ch1 254nm 4nm

Peak#	Ret. Time	Area	Height	Area %	Height %
1	17.524	22044172	400021	100.000	100.000
Total		22044172	400021	100.000	100.000

Figure S53: Chiral HPLC profile of (*S*)-**3db** at 0 min(before placing for incubation at 353 K)

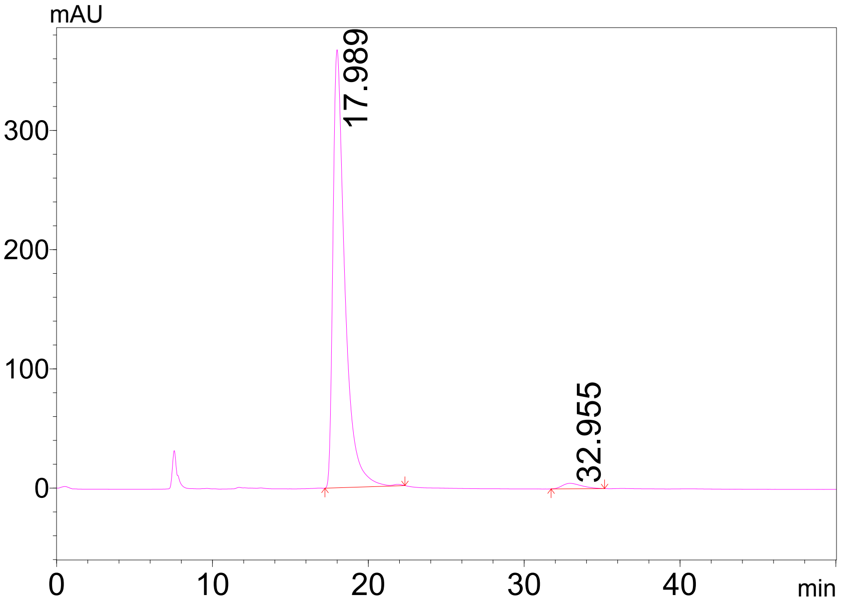

PDA Ch1 254nm 4nm

Peak#	Ret. Time	Area	Height	Area %	Height %
1	17.989	19528277	367382	97.984	98.765
2	32.955	401698	4593	2.016	1.235
Total		19929975	371975	100.000	100.000

Figure S54: Chiral HPLC profile of (*S*)-**3db** at 2 min (353 K)

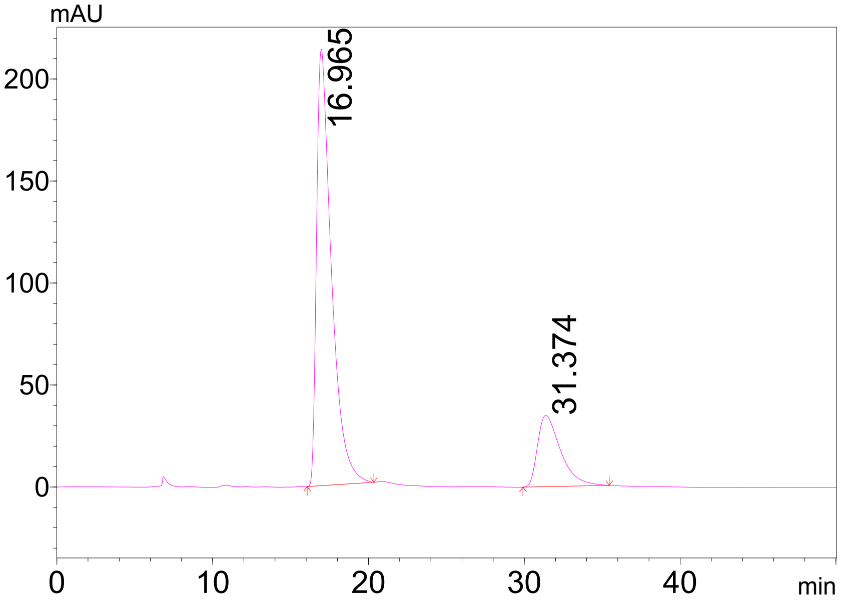

PDA Ch1 254nm 4nm

Peak#	Ret. Time	Area	Height	Area %	Height %
1	16.965	14162244	213987	79.731	85.960
2	31.374	3600248	34951	20.269	14.040
Total		17762492	248938	100.000	100.000

Figure S55: Chiral HPLC profile of (*S*)-**3db** at 5 min (353 K)

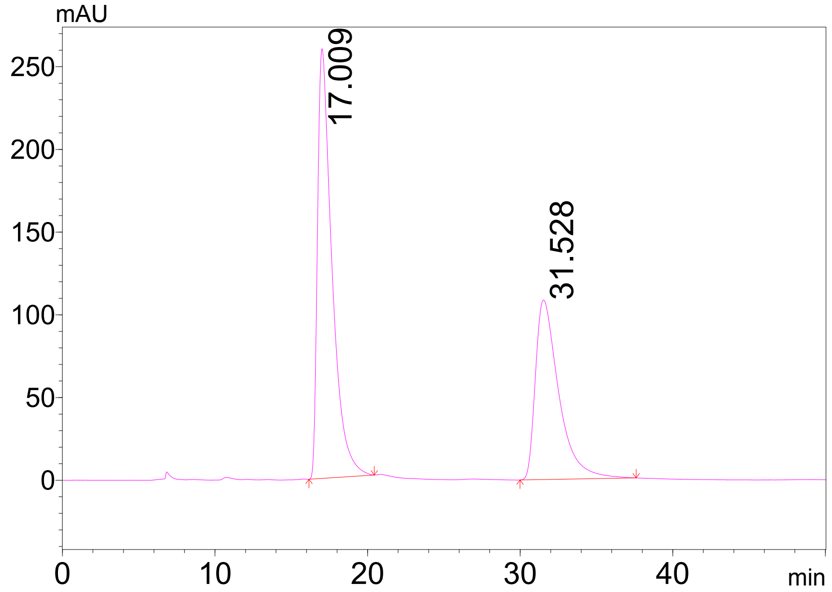

PDA Ch1 254nm 4nm

Peak#	Ret. Time	Area	Height	Area %	Height %
1	17.009	17145530	259764	59.719	70.526
2	31.528	11564742	108559	40.281	29.474
Total		28710272	368323	100.000	100.000

Figure S56: Chiral HPLC profile of (*S*)-**3db** at 10 min (353 K)

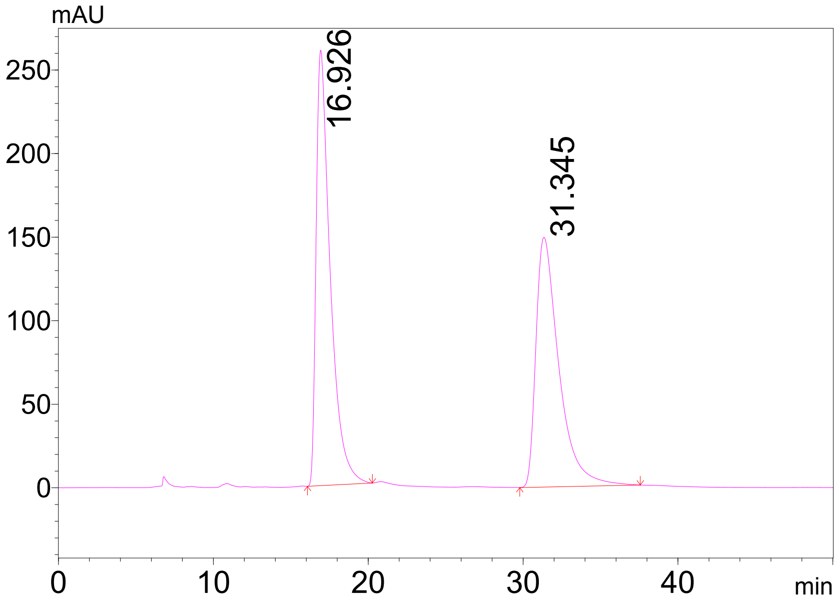

PDA Ch1 254nm 4nm

Peak#	Ret. Time	Area	Height	Area %	Height %
1	16.926	16493874	260548	51.673	63.549
2	31.345	15426135	149450	48.327	36.451
Total		31920009	409999	100.000	100.000

Figure S57: Chiral HPLC profile of (*S*)-**3db** at 15 min (353 K)

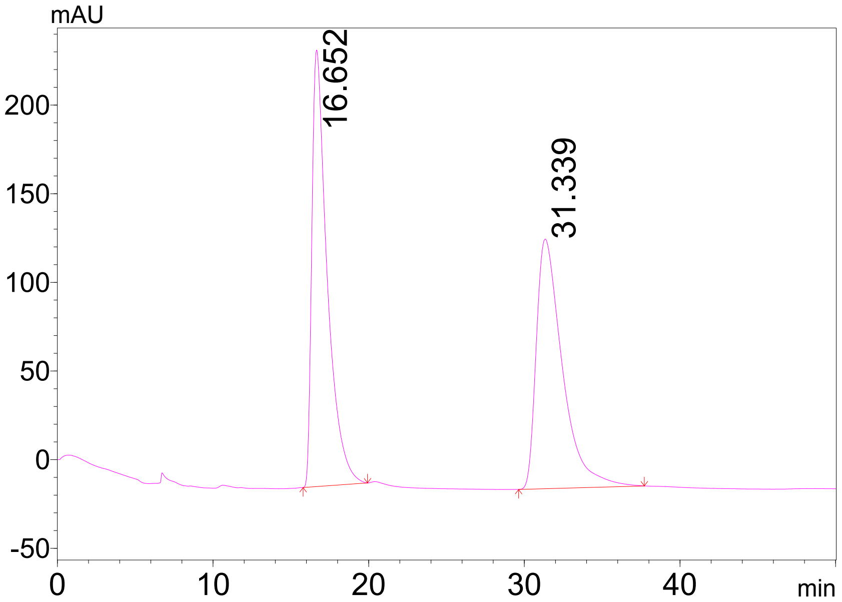

PDA Ch1 254nm 4nm

Peak#	Ret. Time	Area	Height	Area %	Height %
1	16.652	16891318	246188	50.687	63.621
2	31.339	16433725	140771	49.313	36.379
Total		33325043	386959	100.000	100.000

Figure S58: Chiral HPLC profile of (*S*)-**3db** at 20 min (353 K)

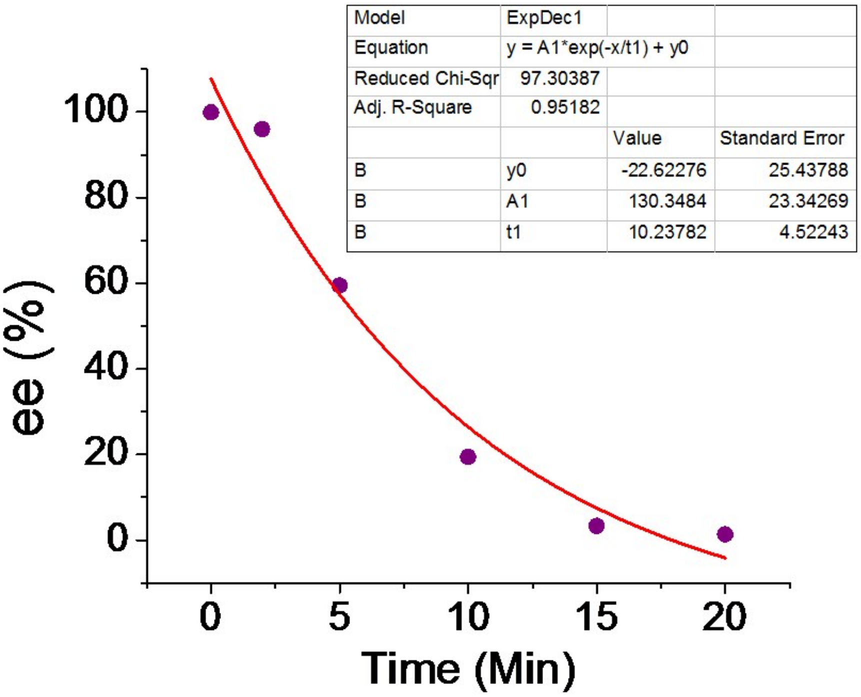

Figure S59: Plot of ee (%) of (*S*)-**3db** as a function of time (min) at 353 K

[b] Racemization kinetics study of (*S*)-3db at 333 K

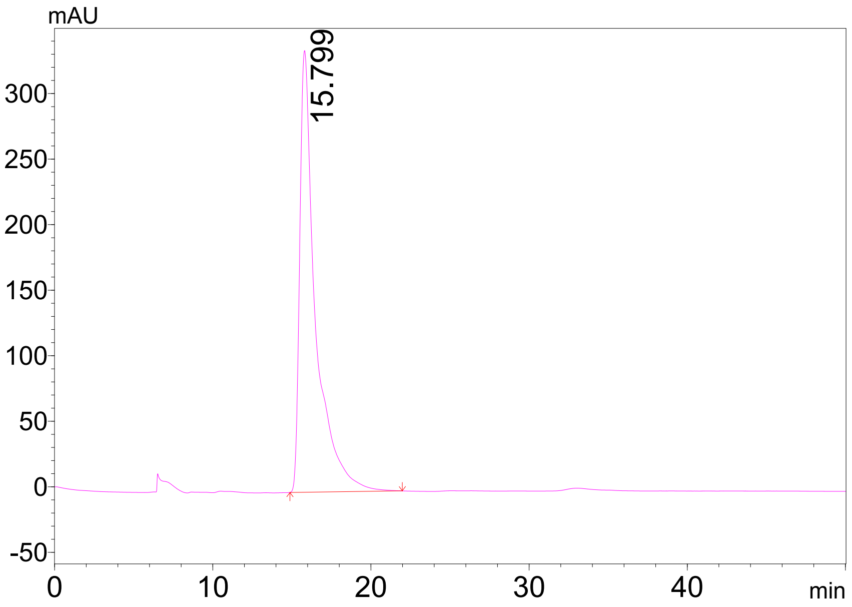

PDA Ch1 254nm 4nm

Peak#	Ret. Time	Area	Height	Area %	Height %
1	15.799	23373921	336907	100.000	100.000
Total		23373921	336907	100.000	100.000

Figure S60: Chiral HPLC profile of (*S*)-**3db** at 0 min (before placing incubation at 333 K)

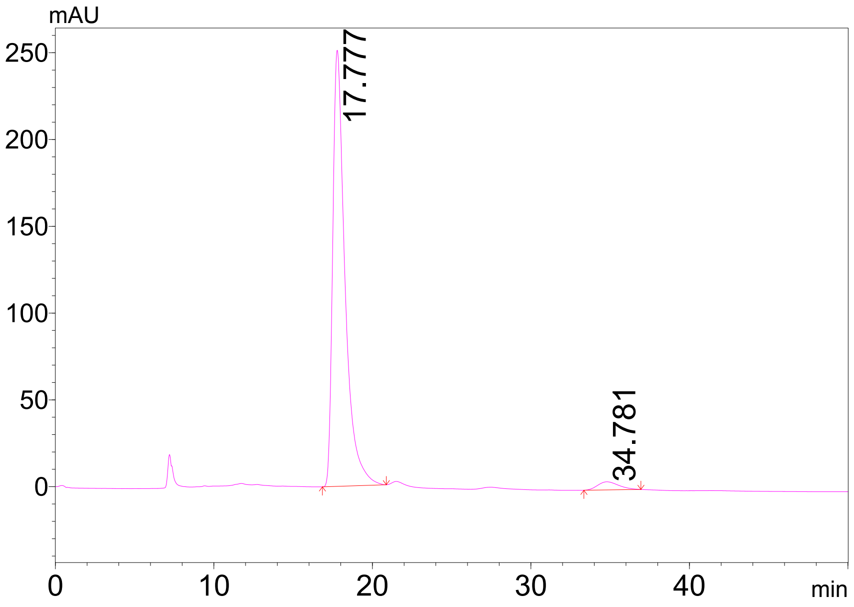

PDA Ch1 254nm 4nm

Peak#	Ret. Time	Area	Height	Area %	Height %
1	17.777	13560561	251318	96.897	98.171
2	34.781	434208	4683	3.103	1.829
Total		13994770	256001	100.000	100.000

Figure S61: Chiral HPLC profile of (*S*)-**3db** at 10 min (333 K)

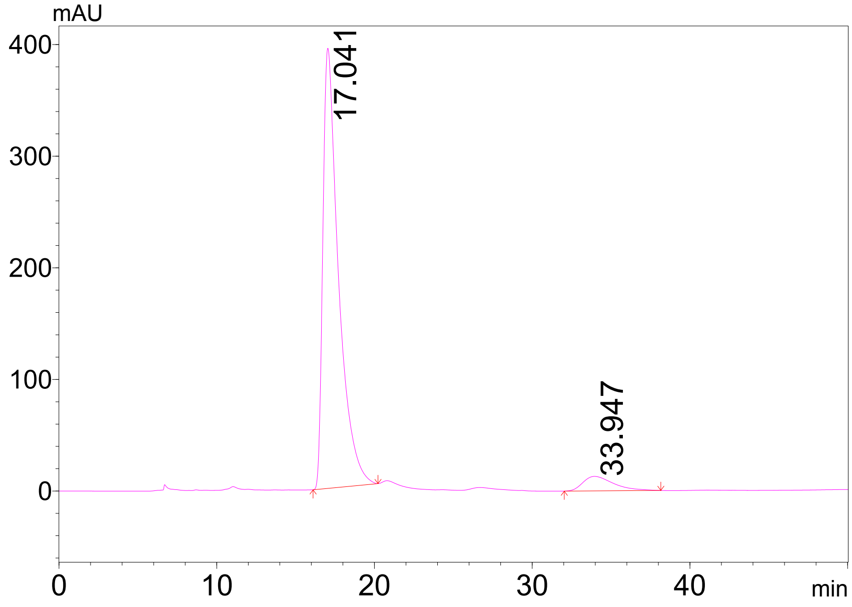

PDA Ch1 254nm 4nm

Peak#	Ret. Time	Area	Height	Area %	Height %
1	17.041	27205714	394366	93.959	96.813
2	33.947	1749107	12983	6.041	3.187
Total		28954821	407349	100.000	100.000

Figure S62: Chiral HPLC profile of (*S*)-**3db** at 20 min (333 K)

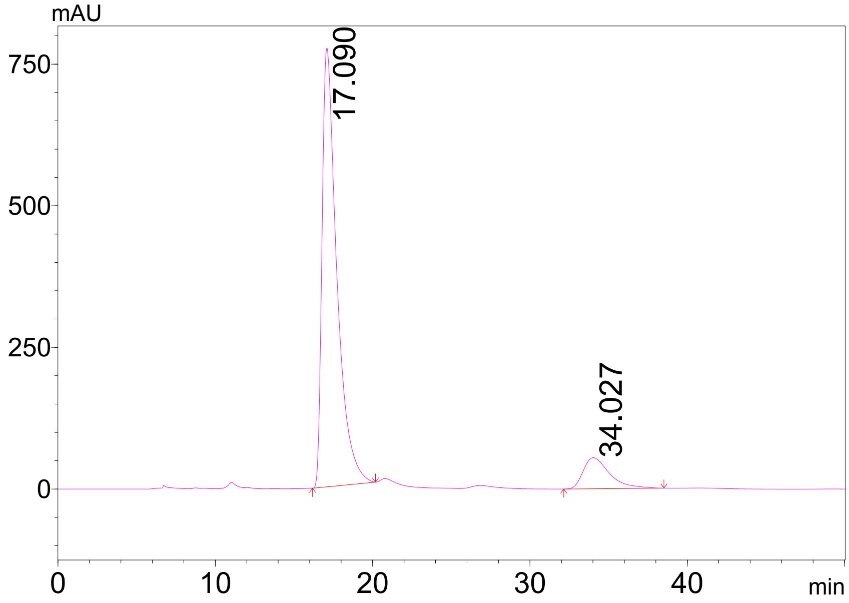

PDA Ch1 254nm 4nm

Peak#	Ret. Time	Area	Height	Area %	Height %
1	17.090	50622093	774536	88.755	93.371
2	34.027	6413374	54993	11.245	6.629
Total		57035467	829529	100.000	100.000

Figure S63: Chiral HPLC profile of (*S*)-**3db** at 40 min (333 K)

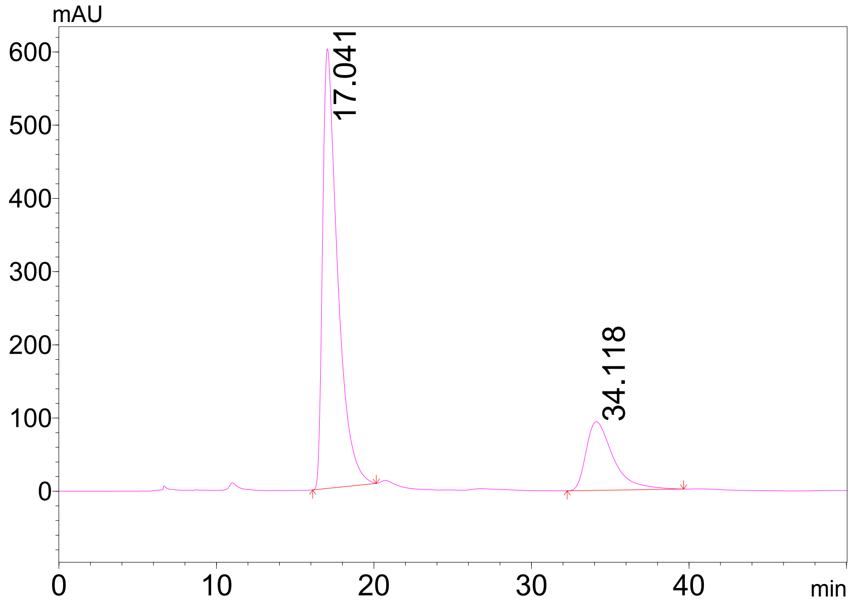

PDA Ch1 254nm 4nm

Peak#	Ret. Time	Area	Height	Area %	Height %
1	17.041	39855403	600390	78.014	86.491
2	34.118	11232031	93772	21.986	13.509
Total		51087434	694161	100.000	100.000

Figure S64: Chiral HPLC profile of (*S*)-**3db** at 60 min (333 K)

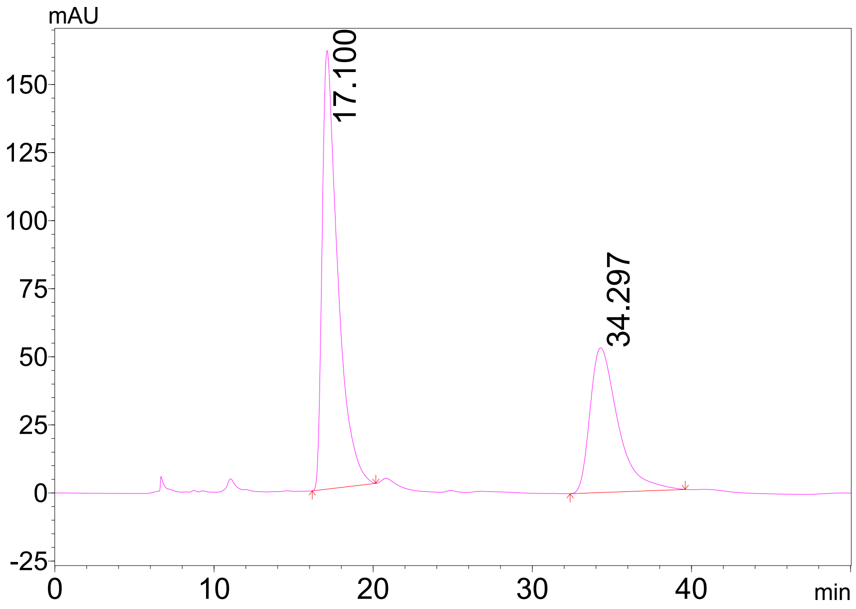

PDA Ch1 254nm 4nm

Peak#	Ret. Time	Area	Height	Area %	Height %
1	17.100	11058181	161064	62.962	75.188
2	34.297	6504958	53151	37.038	24.812
Total		17563140	214215	100.000	100.000

Figure S65: Chiral HPLC profile of (*S*)-**3db** at 180 min (333 K)

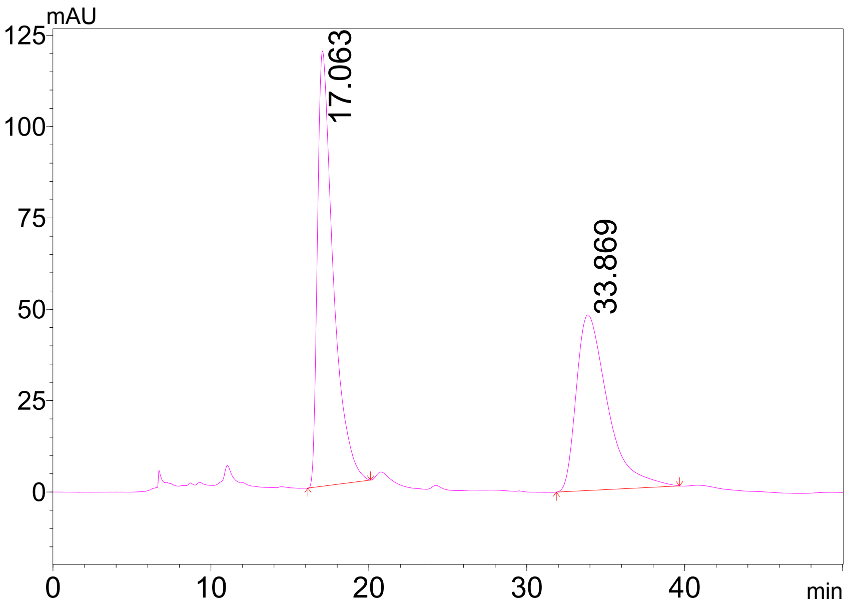

PDA Ch1 254nm 4nm

Peak#	Ret. Time	Area	Height	Area %	Height %
1	17.063	8332049	119125	55.590	71.249
2	33.869	6656290	48071	44.410	28.751
Total		14988339	167196	100.000	100.000

Figure S66: Chiral HPLC profile of (*S*)-**3db** at 300 min (333 K)

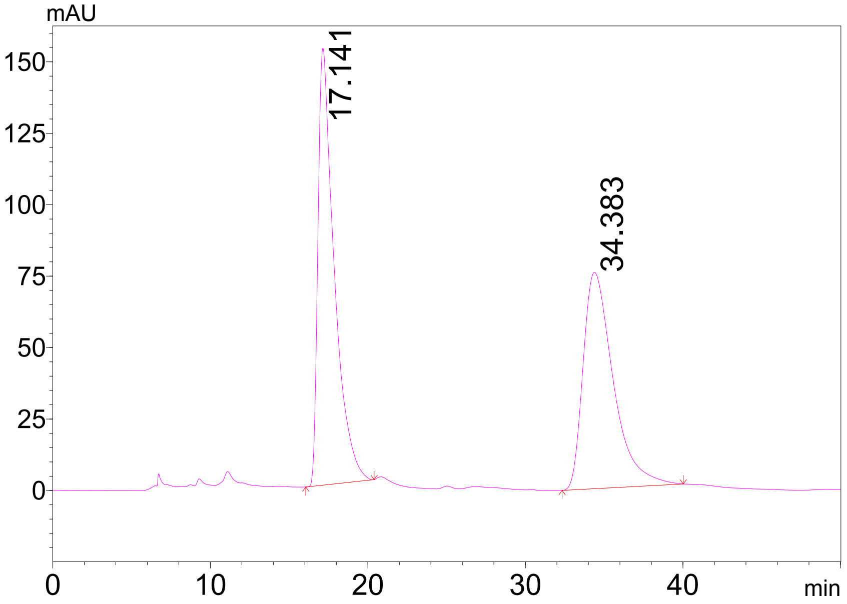

PDA Ch1 254nm 4nm

Peak#	Ret. Time	Area	Height	Area %	Height %
1	17.141	10992815	152910	52.142	66.875
2	34.383	10089624	75740	47.858	33.125
Total		21082439	228649	100.000	100.000

Figure S67: Chiral HPLC profile of (*S*)-**3db** at 600 min (333 K)

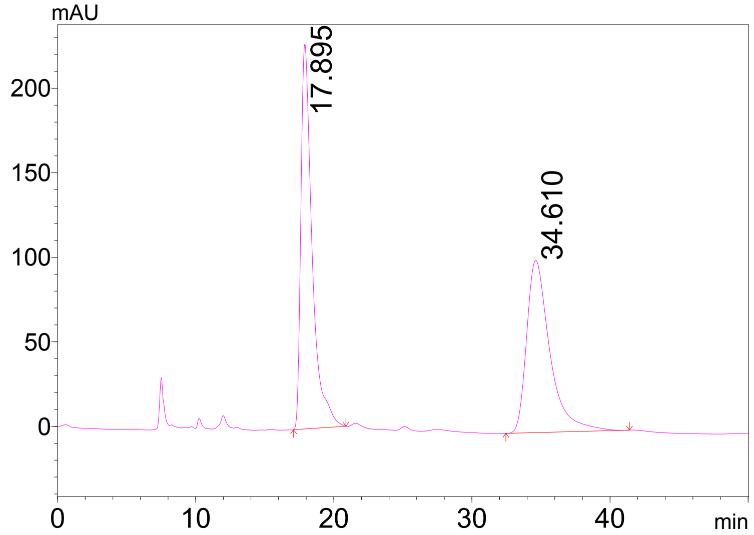

PDA Ch1 254nm 4nm

Peak#	Ret. Time	Area	Height	Area %	Height %
1	17.895	12904652	227658	51.954	69.074
2	34.610	11933955	101928	48.046	30.926
Total		24838607	329585	100.000	100.000

Figure S68: Chiral HPLC profile of (*S*)-**3db** at 780 min (333 K)

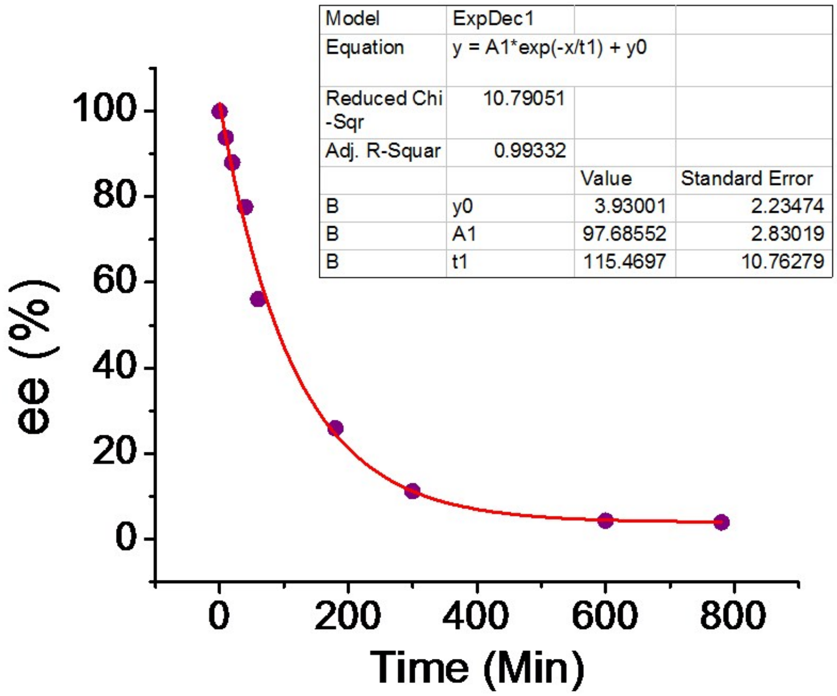

Figure S69: Plot of ee (%) of (*S*)-**3db** as a function of time (min) at 333 K

[c] Racemization kinetics study of (*S*)-3db at 300 K

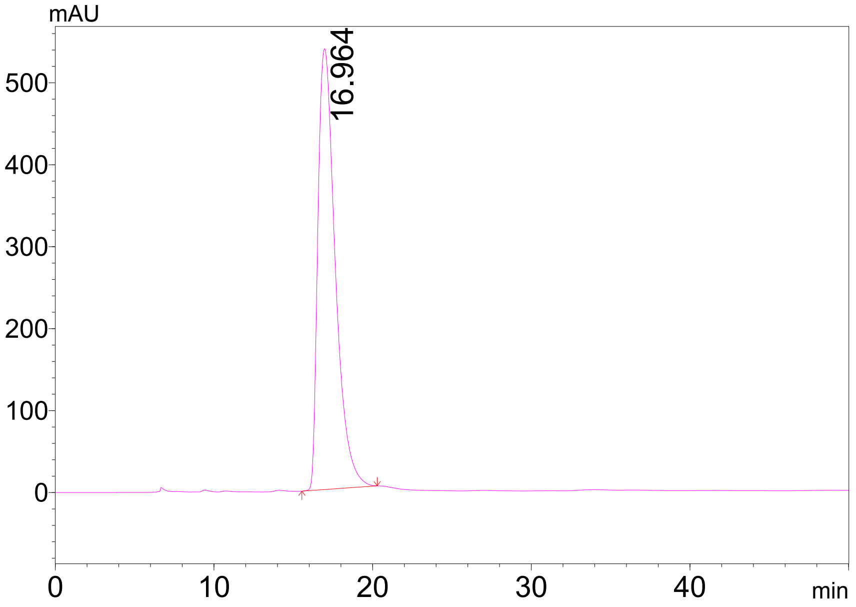

PDA Ch1 254nm 4nm

Peak#	Ret. Time	Area	Height	Area %	Height %
1	16.964	40957693	537914	100.000	100.000
Total		40957693	537914	100.000	100.000

Figure S70: Chiral HPLC profile of (*S*)-**3db** at 0 day (the moment of placing the sample at 300 K)

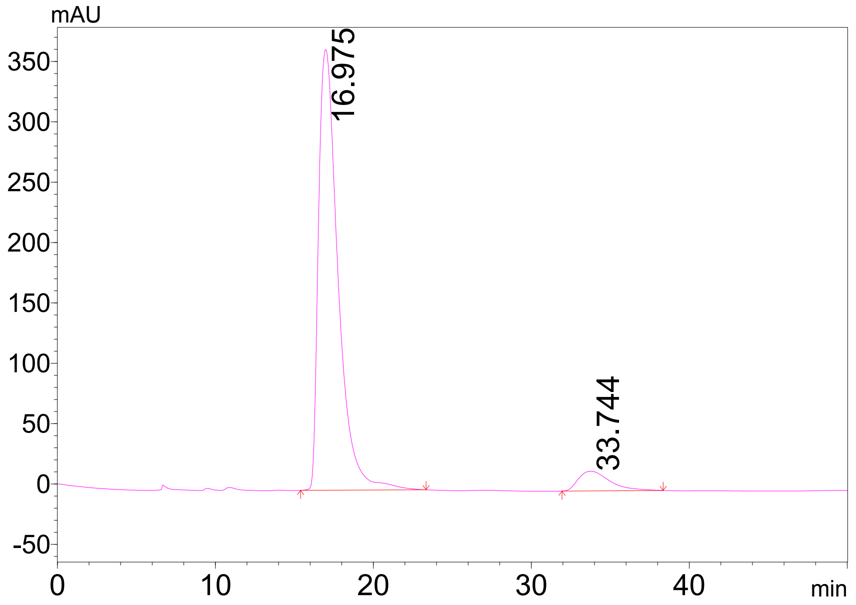

PDA Ch1 254nm 4nm

Peak#	Ret. Time	Area	Height	Area %	Height %
1	16.975	31432394	365050	93.281	95.712
2	33.744	2263958	16356	6.719	4.288
Total		33696351	381406	100.000	100.000

Figure S71: Chiral HPLC profile of (*S*)-**3db** at 4 days (300 K)

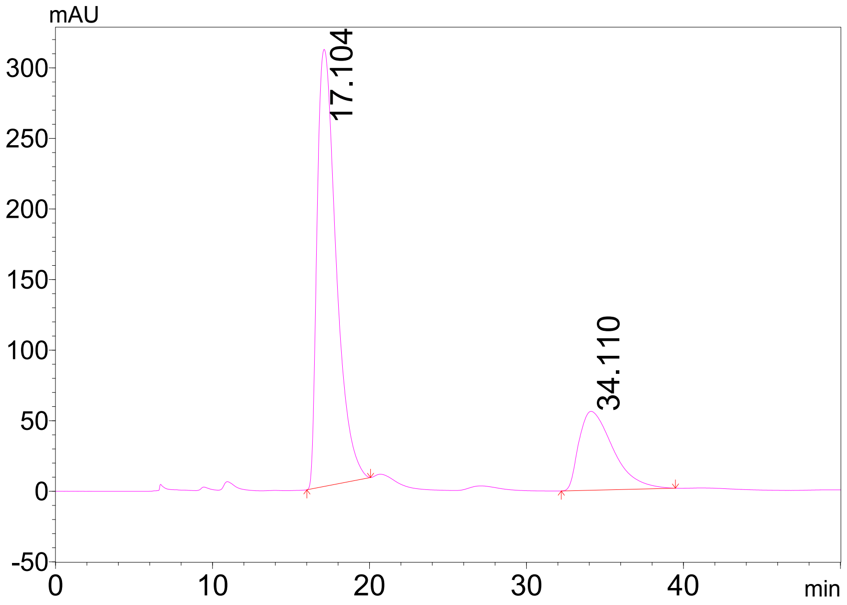

PDA Ch1 254nm 4nm

Peak#	Ret. Time	Area	Height	Area %	Height %
1	17.104	26157549	309714	75.563	84.714
2	34.110	8459120	55885	24.437	15.286
Total		34616669	365599	100.000	100.000

Figure S72: Chiral HPLC profile of (*S*)-**3db** at 8 days (300 K)

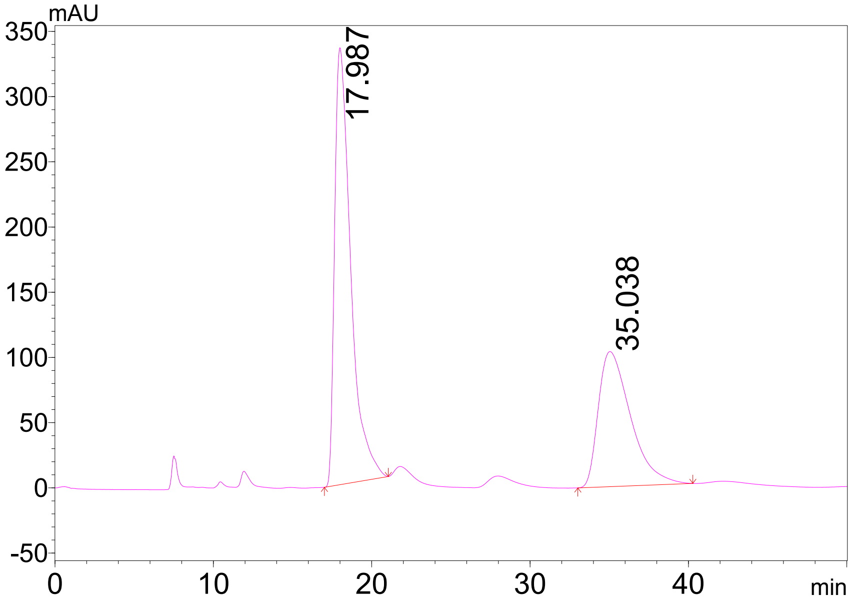

PDA Ch1 254nm 4nm

Peak#	Ret. Time	Area	Height	Area %	Height %
1	17.987	23734306	335059	61.782	76.381
2	35.038	14682074	103608	38.218	23.619
Total		38416380	438667	100.000	100.000

Figure S73: Chiral HPLC profile of (*S*)-**3db** at 10 days (300 K)

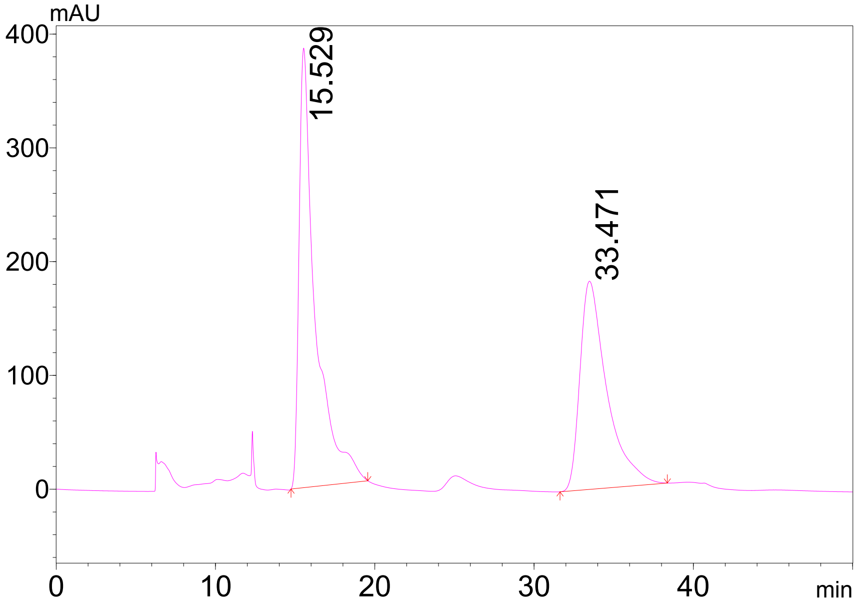

PDA Ch1 254nm 4nm

Peak#	Ret. Time	Area	Height	Area %	Height %
1	15.529	27492908	386311	56.074	67.857
2	33.471	21537114	182994	43.926	32.143
Total		49030022	569305	100.000	100.000

Figure S74: Chiral HPLC profile of (*S*)-**3db** at 15 days (300 K)

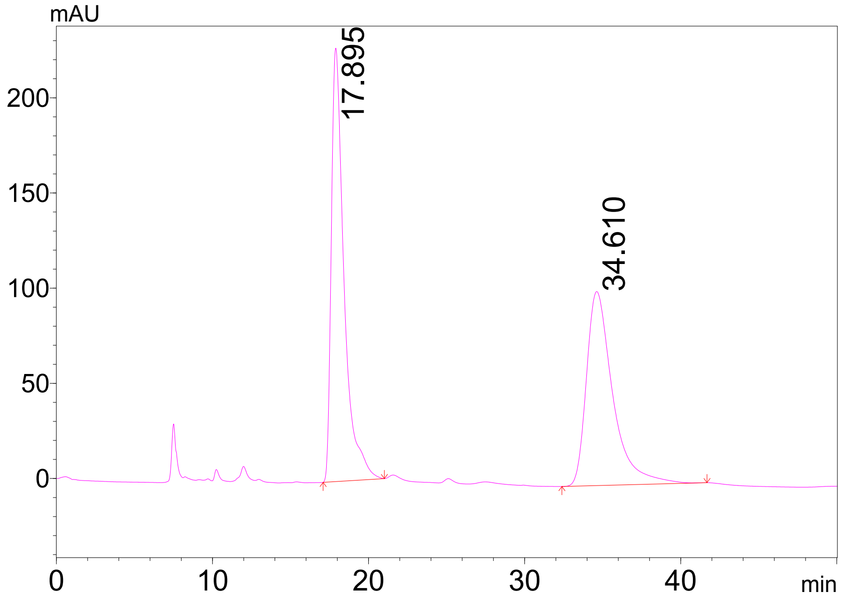

PDA Ch1 254nm 4nm

Peak#	Ret. Time	Area	Height	Area %	Height %
1	17.895	12929305	227703	51.983	69.079
2	34.610	11942963	101925	48.017	30.921
Total		24872268	329627	100.000	100.000

Figure S75: Chiral HPLC profile of (*S*)-**3db** at 20 days (300 K)

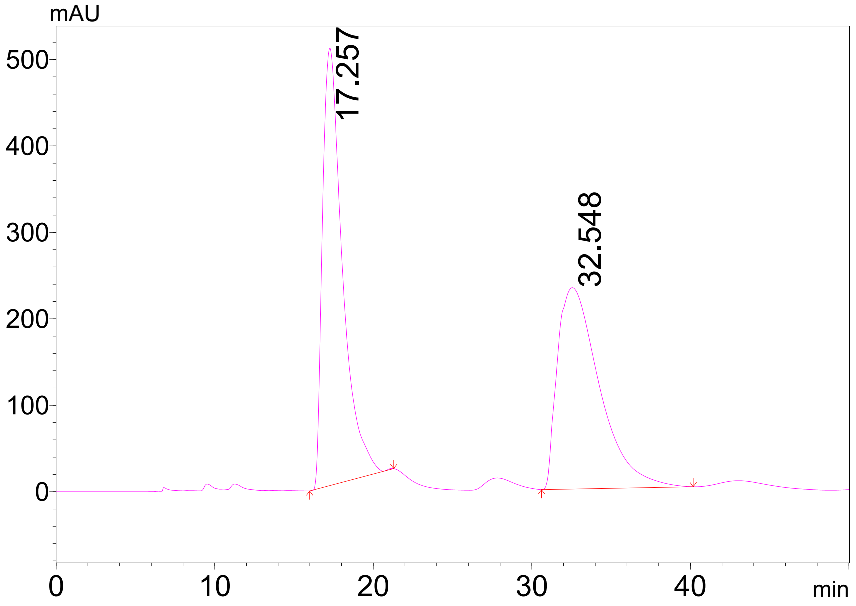

PDA Ch1 254nm 4nm

Peak#	Ret. Time	Area	Height	Area %	Height %
1	17.257	44631687	505903	50.868	68.466
2	32.548	43108591	233009	49.132	31.534
Total		87740278	738912	100.000	100.000

Figure S76: Chiral HPLC profile of (*S*)-**3db** at 23 days (300 K)

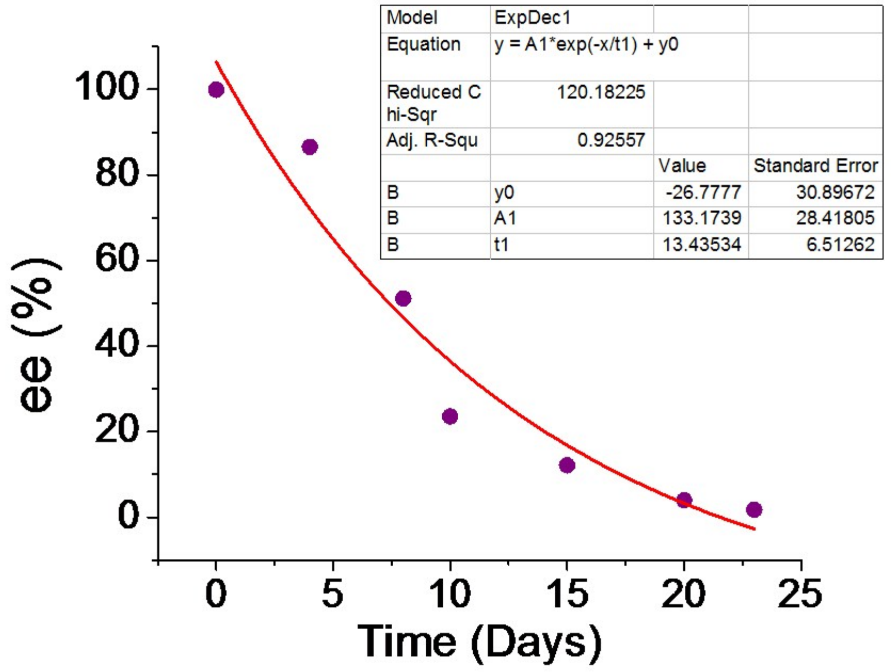

Figure S77: Plot of ee (%) of (*S*)-**3db** as a function of time (days) at 300 K

[d] Racemization kinetics study of (*S*)-3dg at 300 K

Same experimental method employed as in **Section D** for **3dg**. Individual samples were subjected to chiral HPLC analysis by using following condition:

Column: CHIRALCEL OD-H (4.6 mm x 250.0 mm x 5.0 μm)
Solvent: hexane: ethanol (95: 5)
Flow rate: 0.5 mL/min
Mode: Isocratic
Column oven: 298 K
Sample conc.: 1.0 mg/ mL ethanol
Injected volume: 5 μL

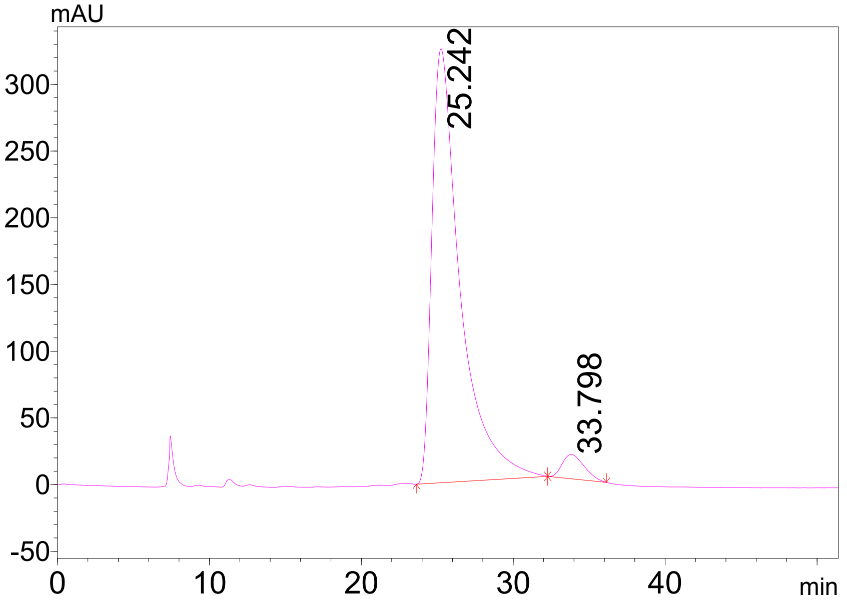

PDA Ch1 254nm 4nm

Peak#	Ret. Time	Area	Height	Area %	Height %
1	25.242	42195368	325223	95.799	94.684
2	33.798	1850344	18258	4.201	5.316
Total		44045711	343481	100.000	100.000

Figure S78: Chiral HPLC profile of (*S*)-**3dg** at 0 h (moment of placing the sample at 300 K) [during the course of isolating optically pure (*S*)-**3dg** from its racemic mixture by HPLC, ee immediately dropped to 91.6%]

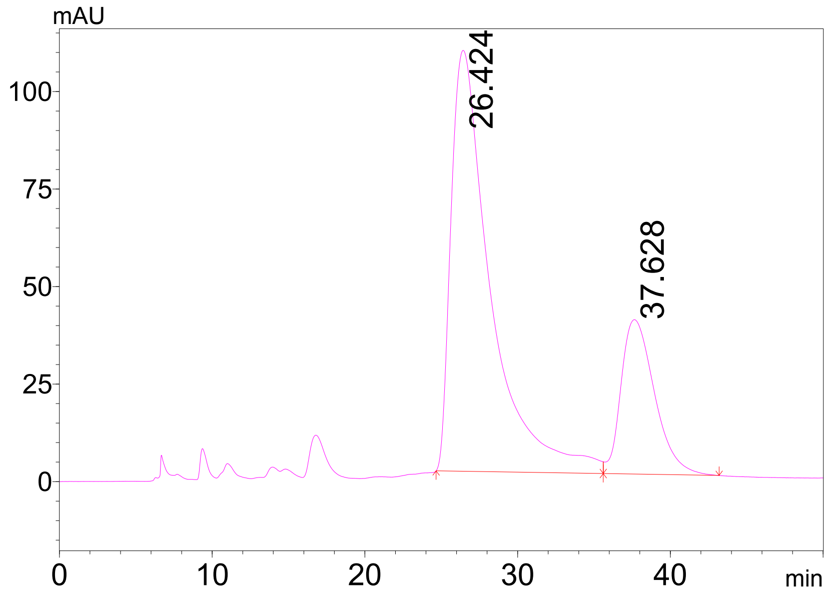

PDA Ch1 254nm 4nm

Peak#	Ret. Time	Area	Height	Area %	Height %
1	26.424	19410755	107890	76.228	73.161
2	37.628	6053262	39580	23.772	26.839
Total		25464017	147469	100.000	100.000

Figure S79: Chiral HPLC profile of (*S*)-**3dg** at 3 h

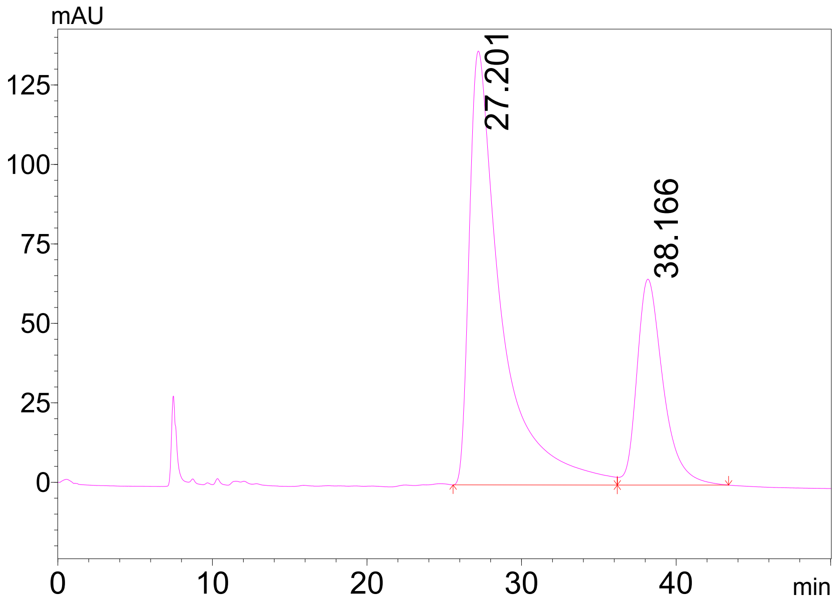

PDA Ch1 254nm 4nm

Peak#	Ret. Time	Area	Height	Area %	Height %
1	27.201	19668331	136492	71.923	67.816
2	38.166	7678178	64775	28.077	32.184
Total		27346509	201267	100.000	100.000

Figure S80: Chiral HPLC profile of (*S*)-**3dg** at 8 h (300 K)

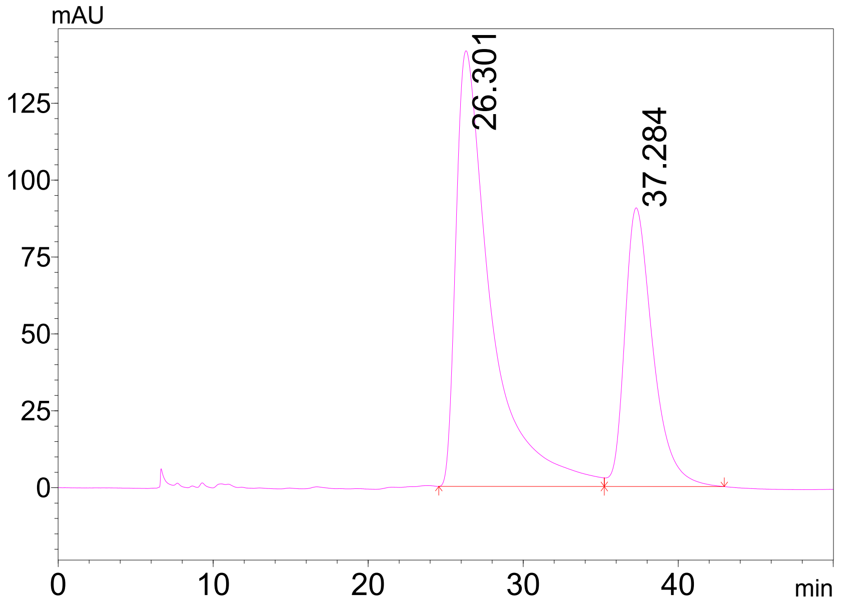

PDA Ch1 254nm 4nm

Peak#	Ret. Time	Area	Height	Area %	Height %
1	26.301	22212913	141636	65.943	60.984
2	37.284	11472243	90617	34.057	39.016
Total		33685156	232253	100.000	100.000

Figure S81: Chiral HPLC profile of (*S*)-**3dg** at 12 h (300 K)

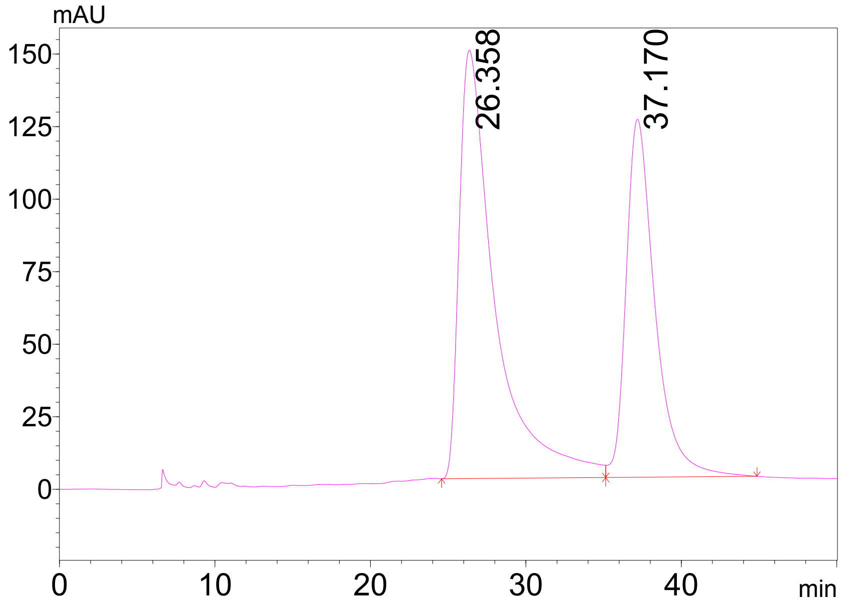

PDA Ch1 254nm 4nm

Peak#	Ret. Time	Area	Height	Area %	Height %
1	26.358	23561871	147681	59.586	54.493
2	37.170	15980672	123328	40.414	45.507
Total		39542543	271009	100.000	100.000

Figure S82: Chiral HPLC profile of (*S*)-**3dg** at 18 h (300 K)

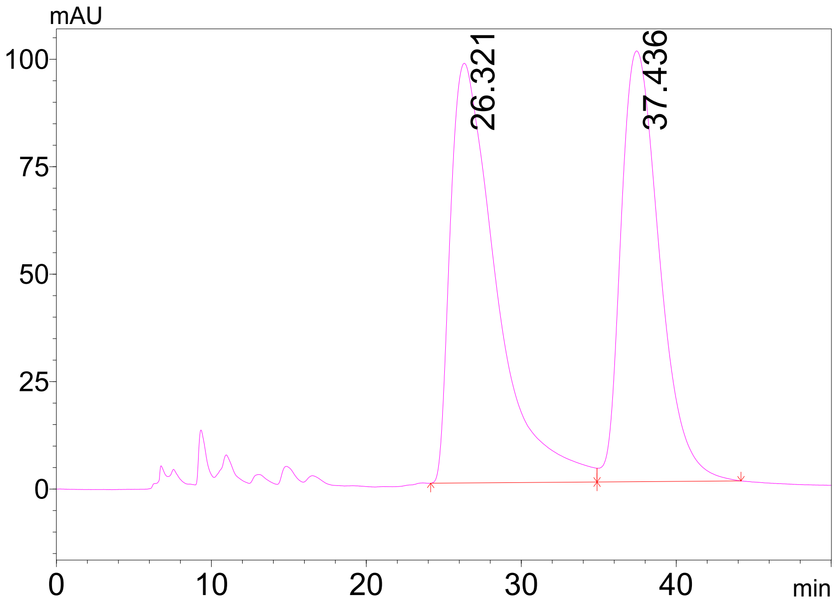

PDA Ch1 254nm 4nm

Peak#	Ret. Time	Area	Height	Area %	Height %
1	26.321	20204999	97637	53.711	49.346
2	37.436	17412698	100227	46.289	50.654
Total		37617697	197864	100.000	100.000

Figure S83: Chiral HPLC profile of (*S*)-**3dg** at 30 h (300 K)

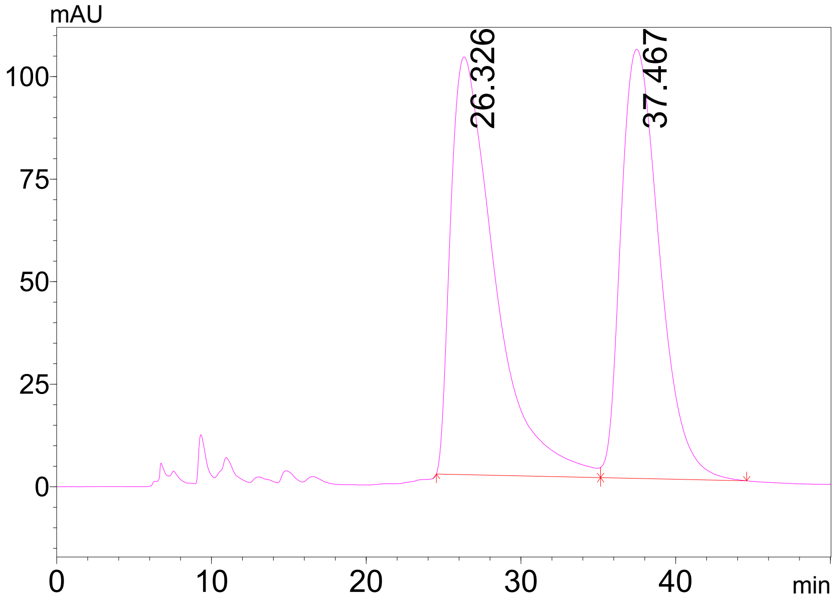

PDA Ch1 254nm 4nm

Peak#	Ret. Time	Area	Height	Area %	Height %
1	26.326	20287479	101830	52.763	49.325
2	37.467	18162673	104618	47.237	50.675
Total		38450152	206448	100.000	100.000

Figure S84: Chiral HPLC profile of (*S*)-**3dg** at 42 h (300 K)

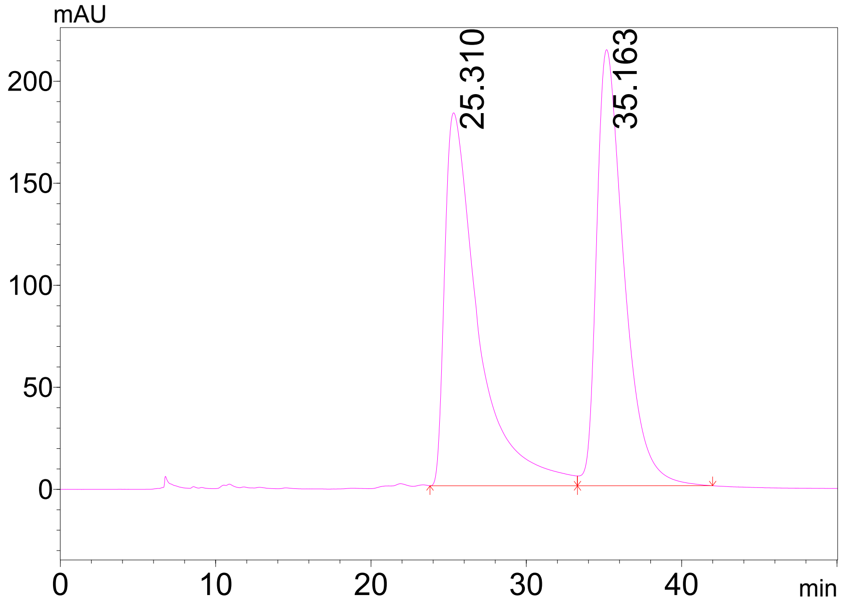

PDA Ch1 254nm 4nm

Peak#	Ret. Time	Area	Height	Area %	Height %
1	25.310	27531822	182753	51.076	46.098
2	35.163	26371436	213687	48.924	53.902
Total		53903258	396439	100.000	100.000

Figure S85: Chiral HPLC profile of (*S*)-**3dg** at 48 h (300 K)

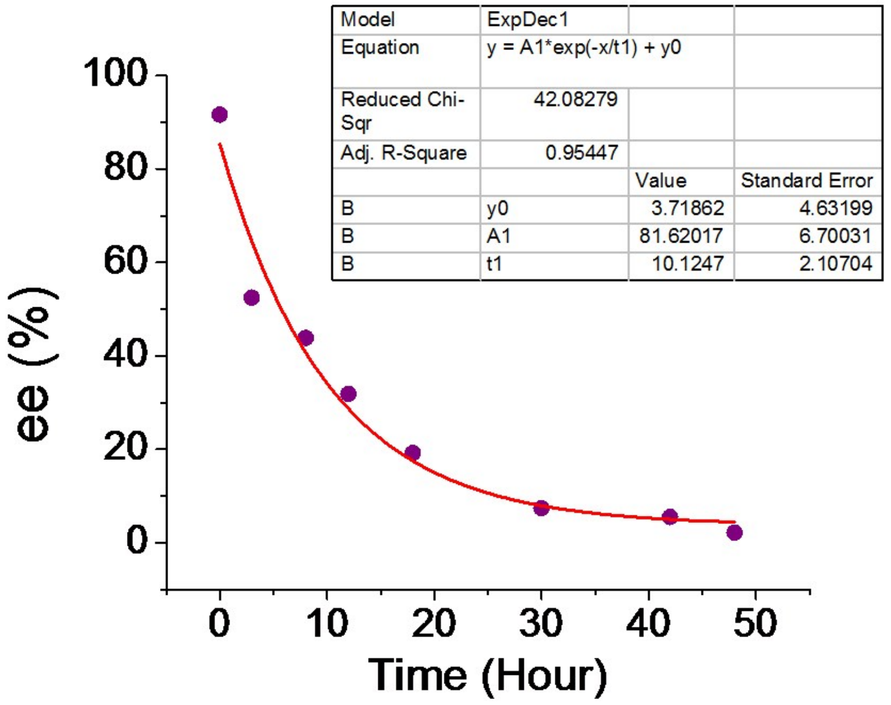

Figure S86: Plot of ee (%) of (*S*)-**3dg** as a function of time (h) at 300 K

[e] Racemization kinetics study of (*S*)-3dh at 300 K

Same experimental method was employed as in **Section D** for **3dh**. Individual samples were subjected to chiral HPLC analysis by using following condition:

Column: CHIRALCEL OD-H (4.6 mm x 250.0 mm x 5.0 μm)
Solvent: hexane: ethanol (90: 10)
Flow rate: 0.5 mL/min
Mode: Isocratic
Column oven: 298 K
Sample conc.: 1 mg/ mL ethanol
Injected volume: 5 μL

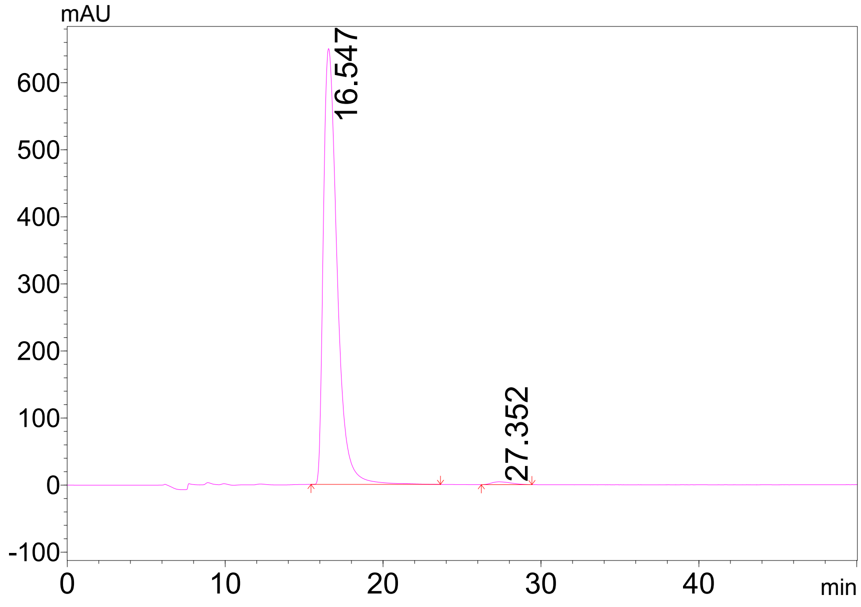

PDA Ch1 254nm 4nm

Peak#	Ret. Time	Area	Height	Area %	Height %
1	16.547	39347341	649835	99.091	99.355
2	27.352	360981	4216	0.909	0.645
Total		39708322	654050	100.000	100.000

Figure S87: Chiral HPLC profile of (*S*)-**3dh** at 0 day (the moment of placing the sample at 300 K)

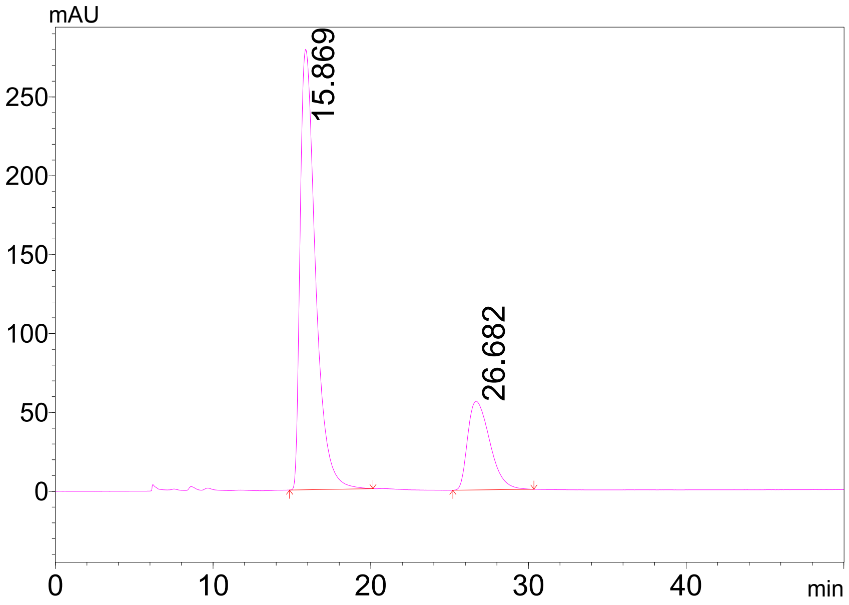

PDA Ch1 254nm 4nm

Peak#	Ret. Time	Area	Height	Area %	Height %
1	15.869	18928146	279208	77.506	83.243
2	26.682	5493224	56204	22.494	16.757
Total		24421370	335412	100.000	100.000

Figure S88: Chiral HPLC profile of (*S*)-**3dh** at 2 days (300 K)

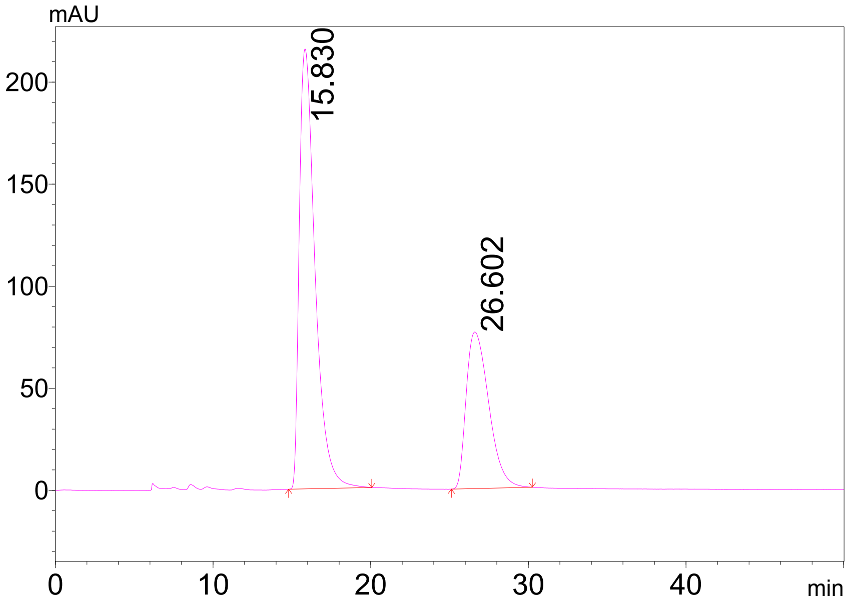

PDA Ch1 254nm 4nm

Peak#	Ret. Time	Area	Height	Area %	Height %
1	15.830	15002031	215505	66.265	73.745
2	26.602	7637553	76723	33.735	26.255
Total		22639584	292228	100.000	100.000

Figure S89: Chiral HPLC profile of (*S*)-**3dh** at 4 days (300 K)

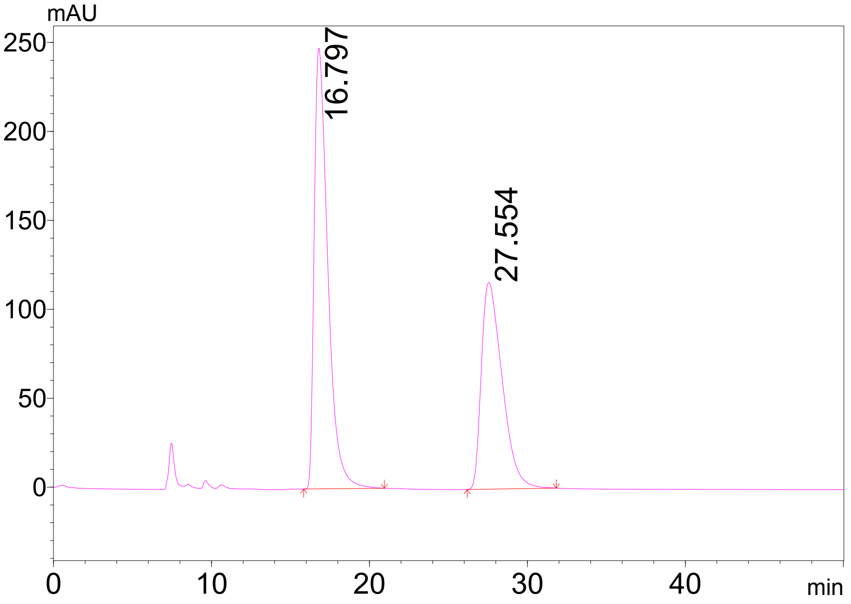

PDA Ch1 254nm 4nm

Peak#	Ret. Time	Area	Height	Area %	Height %
1	16.797	14873154	247773	58.296	68.057
2	27.554	10639994	116291	41.704	31.943
Total		25513148	364064	100.000	100.000

Figure S90: Chiral HPLC profile of (*S*)-**3dh** at 6 days (300 K)

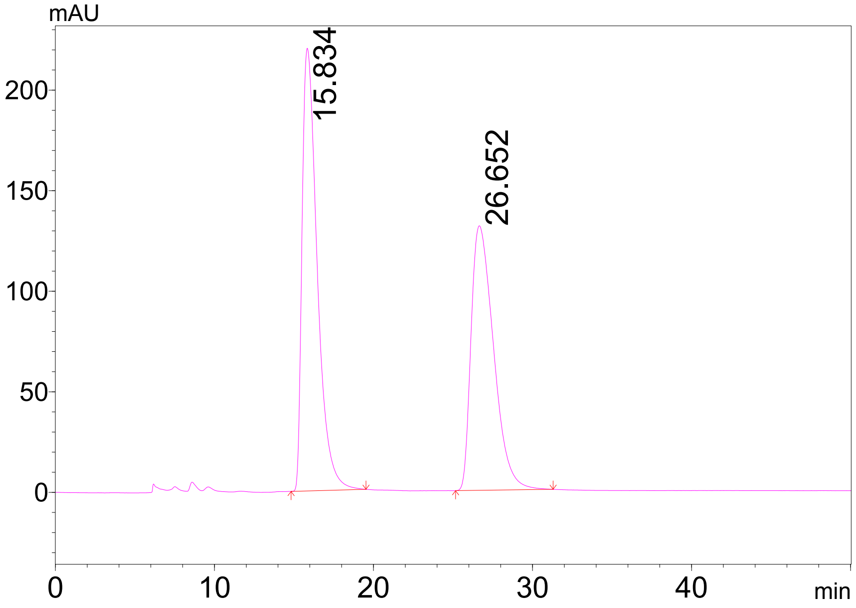

PDA Ch1 254nm 4nm

Peak#	Ret. Time	Area	Height	Area %	Height %
1	15.834	14589978	220163	53.012	62.600
2	26.652	12932050	131538	46.988	37.400
Total		27522027	351701	100.000	100.000

Figure S91: Chiral HPLC profile of (*S*)-**3dh** at 8 days (300 K)

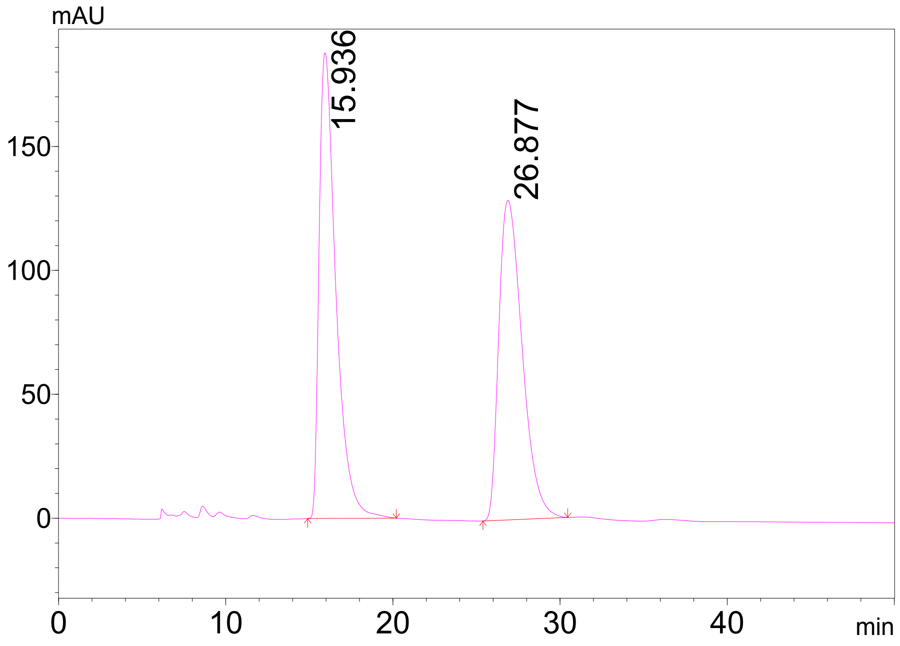

PDA Ch1 254nm 4nm

Peak#	Ret. Time	Area	Height	Area %	Height %
1	15.936	13118432	187913	51.129	59.301
2	26.877	12539106	128969	48.871	40.699
Total		25657538	316882	100.000	100.000

Figure S92: Chiral HPLC profile of (*S*)-**3dh** at 10 days (300 K)

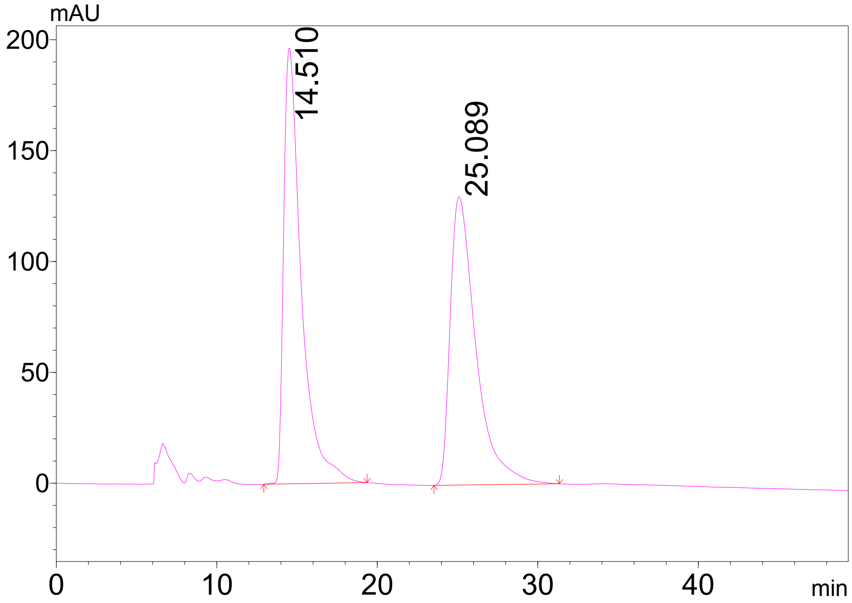

PDA Ch1 254nm 4nm

Peak#	Ret. Time	Area	Height	Area %	Height %
1	14.510	15076071	196530	51.067	60.194
2	25.089	14445829	129963	48.933	39.806
Total		29521900	326493	100.000	100.000

Figure S93: Chiral HPLC profile of (*S*)-**3dh** at 12 days (300 K)

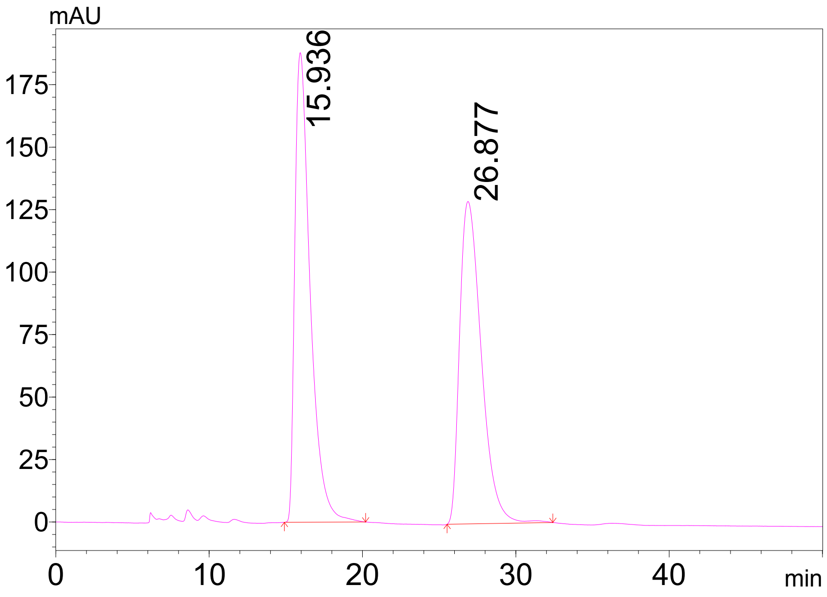

PDA Ch1 254nm 4nm

Peak#	Ret. Time	Area	Height	Area %	Height %
1	15.936	13118432	187913	50.829	59.288
2	26.877	12690276	129035	49.171	40.712
Total		25808708	316948	100.000	100.000

Figure S94: Chiral HPLC profile of (*S*)-**3dh** at 14 days (300 K)

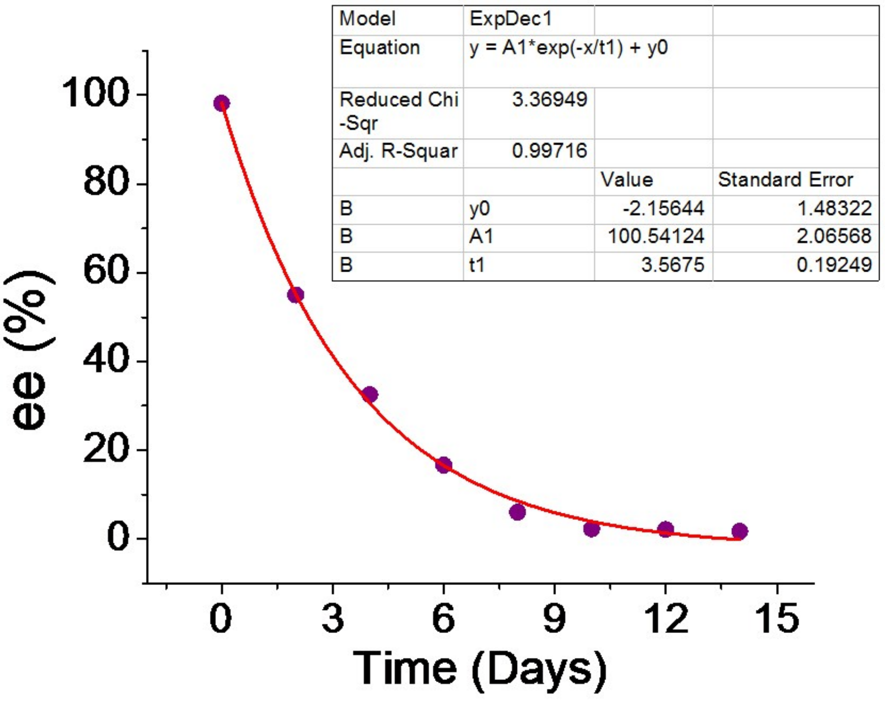

Figure S95: Plot of ee (%) of (*S*)-**3dh** as a function of time (days) at 300 K

[f] Racemization kinetics study of (*S*)-3di at 300 K

Same experimental method was employed as in **Section D** for **3di**. Individual samples were subjected to chiral HPLC analysis by using following condition:

Column: CHIRALCEL OD-H (4.6 mm x 250.0 mm x 5.0 µm)

Solvent: hexane: ethanol (90: 10)

Flow rate: 0.5 mL/min

Mode: Isocratic

Column oven: 298 K

Sample conc.: 1 mg/ mL ethanol

Injected volume: 5 µL

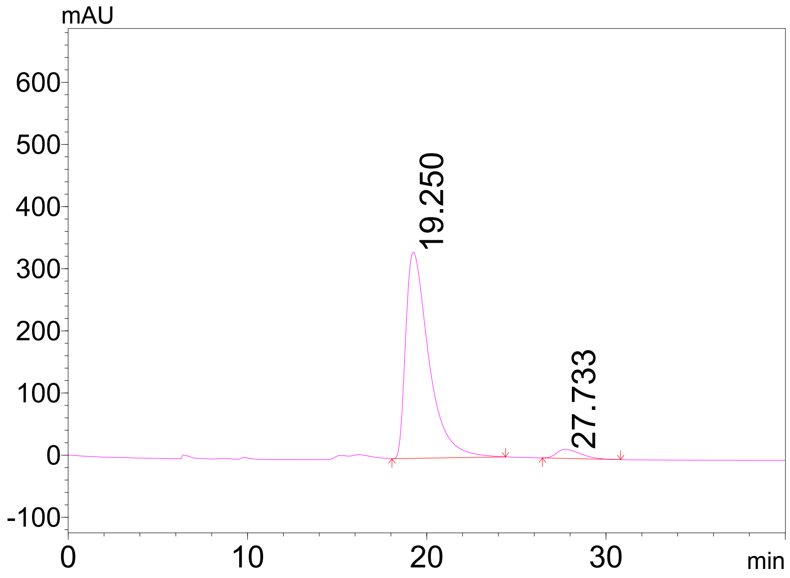

PDA Ch1 254nm 4nm

Peak#	Ret. Time	Area	Height	Area %	Height %
1	19.250	29893374	331643	95.639	95.729
2	27.733	1362974	14797	4.361	4.271
Total		31256349	346440	100.000	100.000

Figure S96: Chiral HPLC profile of (*S*)-**3di** at 0 h (moment of placing the sample at 300 K) [during the course of isolating optically pure (*S*)-**3di** from its racemic mixture by HPLC, ee immediately dropped to 91.3%]

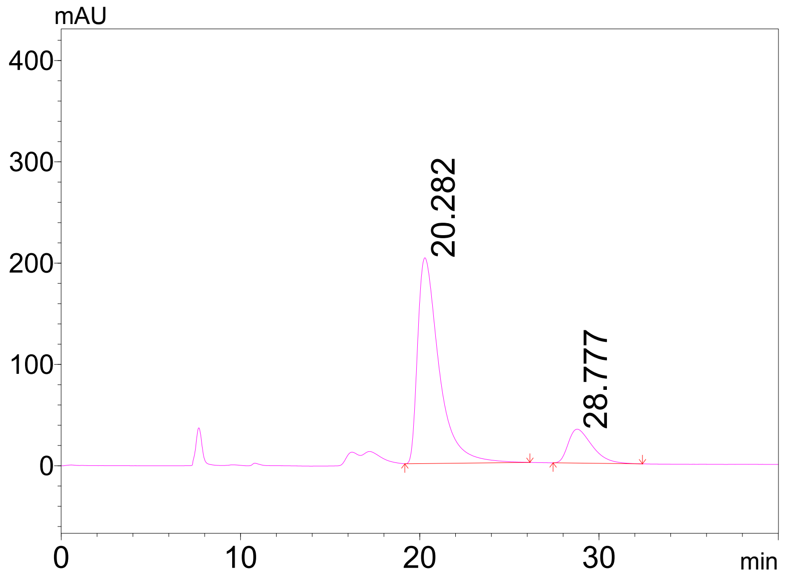

PDA Ch1 254nm 4nm

Peak#	Ret. Time	Area	Height	Area %	Height %
1	20.282	16887866	203095	84.507	85.858
2	28.777	3096208	33452	15.493	14.142
Total		19984074	236546	100.000	100.000

Figure S97: Chiral HPLC profile of (*S*)-**3di** at 1 h (300 K)

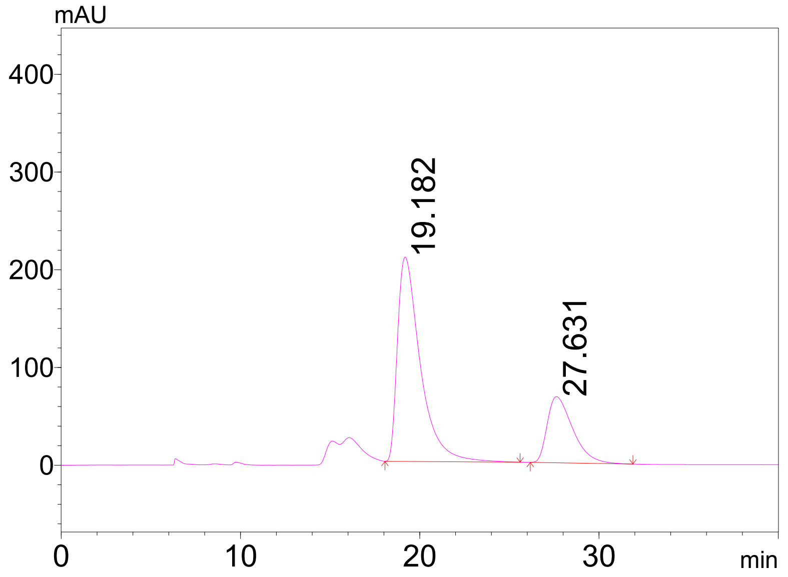

PDA Ch1 254nm 4nm

Peak#	Ret. Time	Area	Height	Area %	Height %
1	19.182	18571085	209125	73.499	75.539
2	27.631	6695937	67720	26.501	24.461
Total		25267022	276845	100.000	100.000

Figure S98: Chiral HPLC profile of (*S*)-**3di** at 2 h (300 K)

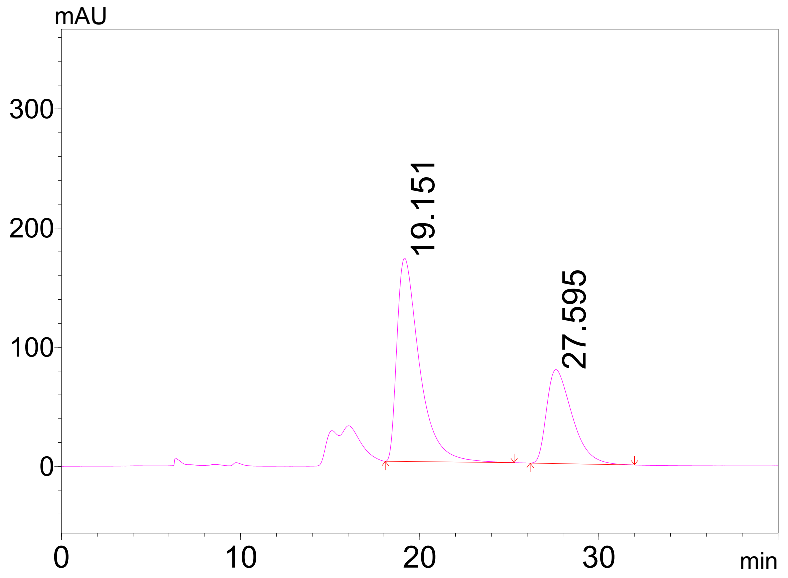

PDA Ch1 254nm 4nm

Peak#	Ret. Time	Area	Height	Area %	Height %
1	19.151	14939994	170684	65.705	68.384
2	27.595	7798058	78913	34.295	31.616
Total		22738052	249597	100.000	100.000

Figure S99: Chiral HPLC profile of (*S*)-**3di** at 3 h (300 K)

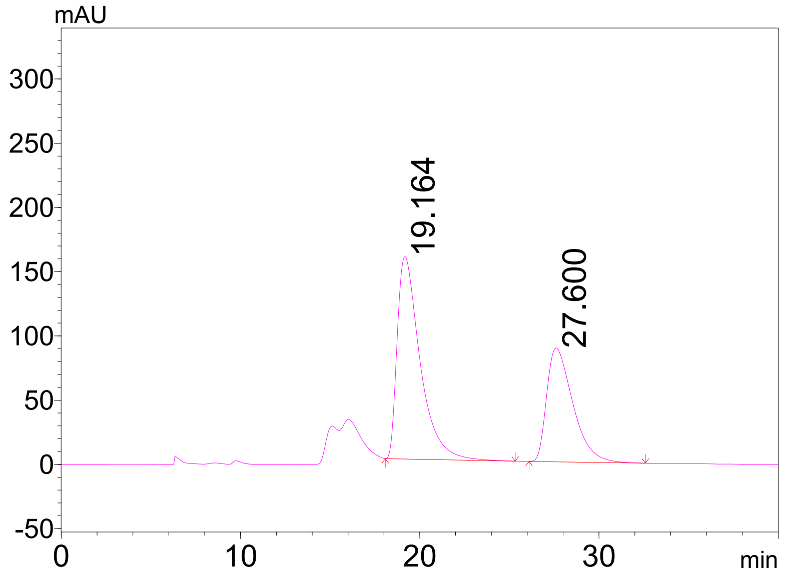

PDA Ch1 254nm 4nm

Peak#	Ret. Time	Area	Height	Area %	Height %
1	19.164	14028625	157488	60.930	63.998
2	27.600	8995682	88595	39.070	36.002
Total		23024306	246083	100.000	100.000

Figure S100: Chiral HPLC profile of (*S*)-**3di** at 4 h (300 K)

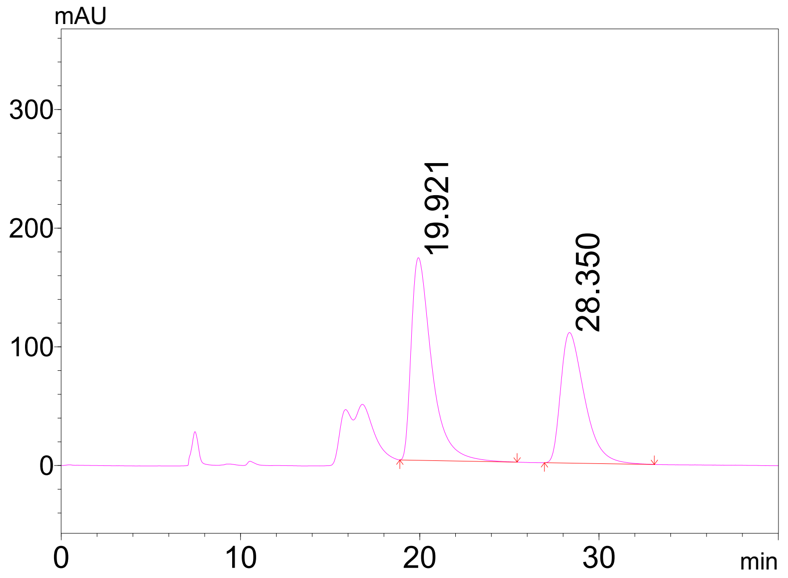

PDA Ch1 254nm 4nm

Peak#	Ret. Time	Area	Height	Area %	Height %
1	19.921	13594606	170715	57.236	60.804
2	28.350	10157035	110049	42.764	39.196
Total		23751641	280764	100.000	100.000

Figure S101: Chiral HPLC profile of (*S*)-**3di** at 5 h (300 K)

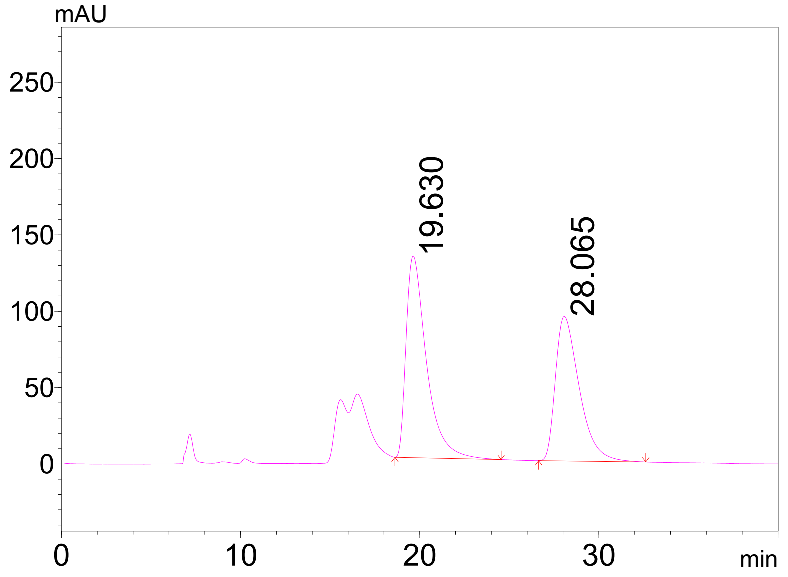

PDA Ch1 254nm 4nm

Peak#	Ret. Time	Area	Height	Area %	Height %
1	19.630	10447859	132071	54.372	58.238
2	28.065	8767738	94706	45.628	41.762
Total		19215597	226778	100.000	100.000

Figure S102: Chiral HPLC profile of (*S*)-**3di** at 6 h (300 K)

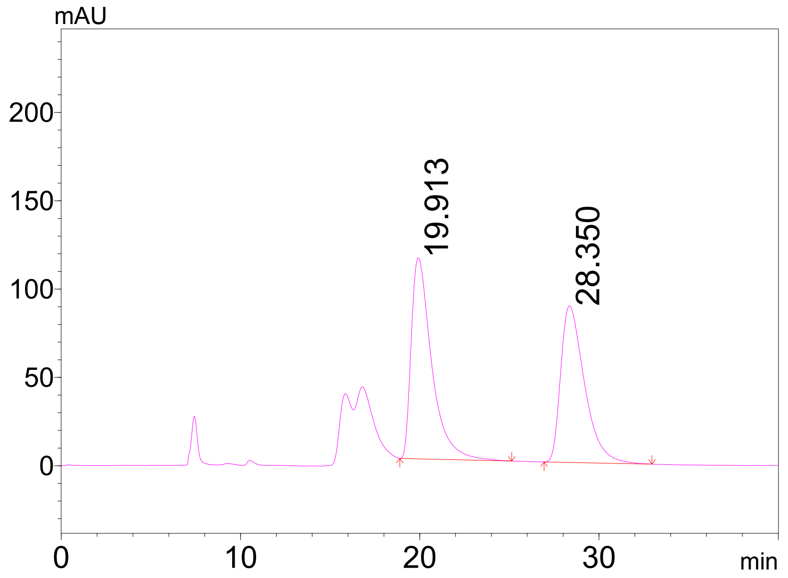

PDA Ch1 254nm 4nm

Peak#	Ret. Time	Area	Height	Area %	Height %
1	19.913	9056158	113812	52.541	56.209
2	28.350	8180297	88667	47.459	43.791
Total		17236455	202479	100.000	100.000

Figure S103: Chiral HPLC profile of (*S*)-**3di** at 7 h (300 K)

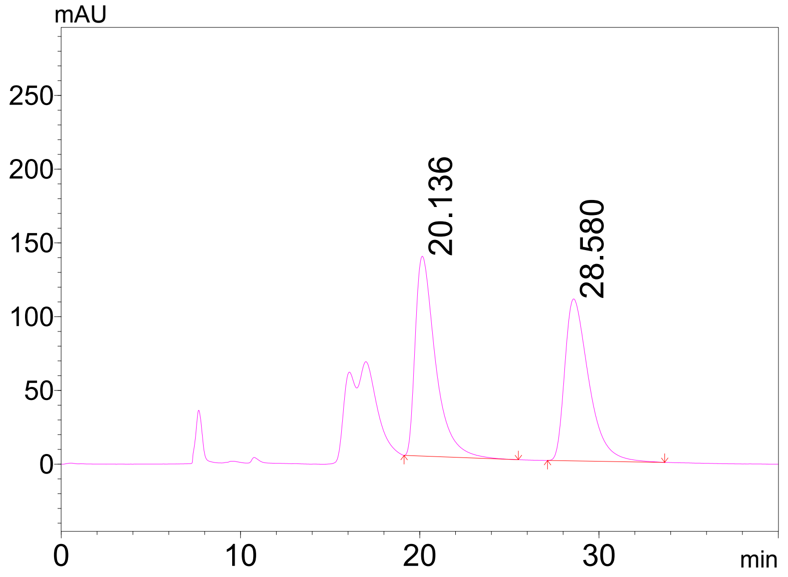

PDA Ch1 254nm 4nm

Peak#	Ret. Time	Area	Height	Area %	Height %
1	20.136	10658564	135466	51.013	55.226
2	28.580	10235402	109826	48.987	44.774
Total		20893965	245293	100.000	100.000

Figure S104: Chiral HPLC profile of (*S*)-**3di** at 8 h (300 K)

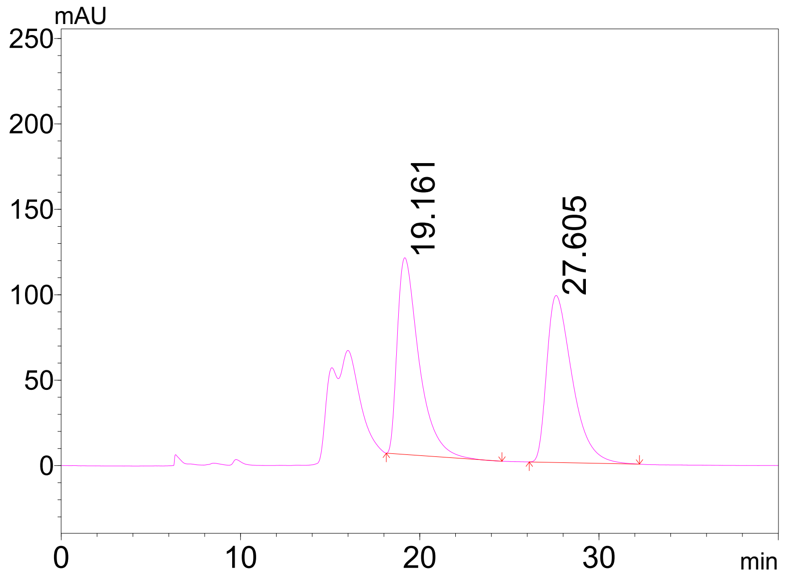

PDA Ch1 254nm 4nm

Peak#	Ret. Time	Area	Height	Area %	Height %
1	19.161	9699857	115138	50.077	54.084
2	27.605	9670089	97749	49.923	45.916
Total		19369945	212887	100.000	100.000

Figure S105: Chiral HPLC profile of (*S*)-**3di** at 9 h (300 K)

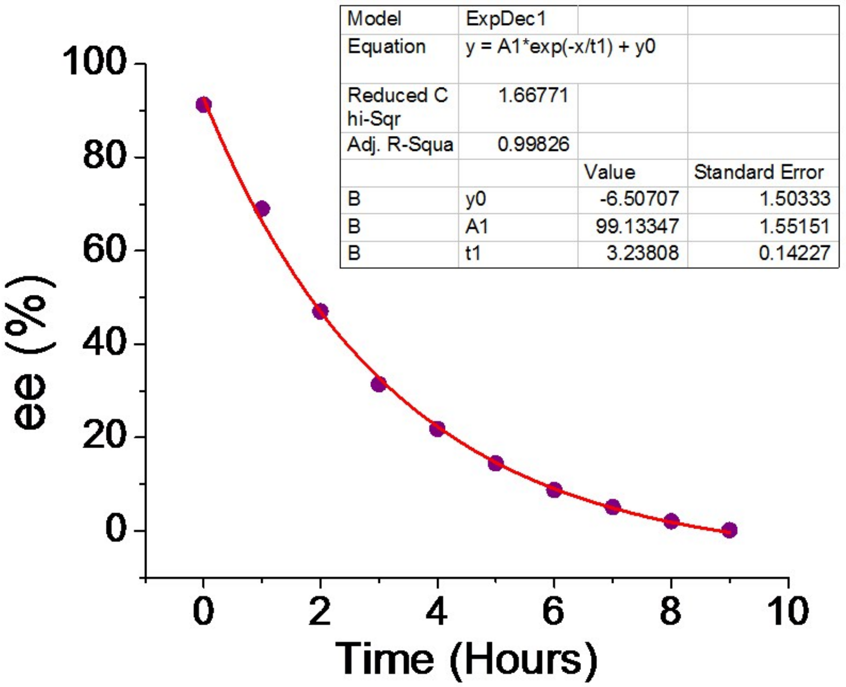

Figure S106: Plot of ee (%) of (*S*)-**3di** as a function of time (h) at 300 K

Section E: Determination of kinetic and thermodynamical parameters of 3db, 3dg, 3dh, and 3di from HPLC analysis

Decrease of enantiomeric excess (*ee*) of (*S*)-**3db** as a function of time (min for 353 K and 333 K, days for 300 K) were plotted at different temperatures (fig. S59, fig. S69 and fig. S77). The decay constants (t_1), rate constants (k_{rac}), and enantiomerisation rate constant (k_{enant}) were determined from the exponential decay curves for each of the temperatures by using the following equations Eqn.1 and 2 respectively.[5,6]

Eqn. 1: $y = A1 * \exp(-\frac{x}{t_1}) + y_0$ (where, A1 = amplitude, t_1 = decay constant and y_0 = offset)

Eqn. 2: $k_{rac} = \frac{1}{t_1} = 2k_{enant}$ (k_{rac} = racemization rate constant, k_{enant} = enantiomerisation rate constant)

The energy ($\Delta G^{\neq}$) barrier for racemization was further calculated using the following Eyring equation (Eqn. 3).

Eqn. 3: $\Delta G^{\neq} = -RT\ln(\frac{hk}{\kappa T k_B})$ (where h = Planck constant, κ = transmission coefficient, T = temperature and k_B = Boltzmann constant)

The activation enthalpy ($\Delta H^{\neq}$) and activation entropy ($\Delta S^{\neq}$) of the isomerization of atropisomer **3db** were further determined employing the Eyring equation (Eqn 4).

Eqn. 4: $\ln\frac{k}{T} = -\frac{\Delta H^{\neq}}{R}\frac{1}{T} + \ln\frac{k_B}{h} + \frac{\Delta S^{\neq}}{R}$

<u>3db at 353K</u>

decay constant = t_1 = 10.23782 min =614.2692 sec
$k_{rac} = 1/ t_1 = 0.001627$
$k_{enant} = 8.13\times10^{-4}$
$\Delta G^{\neq}$ = 25.758 Kcal.mol^{-1}

<u>3db at 333K</u>

decay constant = t_1 = 115.4697 min =6928.182 sec
$k_{rac} = 1/ t_1 = 1.4433\times10^{-4}$
$k_{enant} = 7.21\times10^{-4}$
$\Delta G^{\neq}$ = 25.86 Kcal.mol^{-1}

3db at 300K

decay constant = t_1 = 13.43 day = 1160352 sec
$k_{rac} = 1/ t_1 = 8.61\times10^{-7}$
$k_{enant} = 4.305\times10^{-7}$
$\Delta G^{\neq}$ = 26.29 Kcal.mol^{-1}

This protocol was also applied to determine activation barrier of racemization ($\Delta G^{\neq}$) for **3dg**, **3dh and 3di**.

3dg at 300K

decay constant = t_1 = 10.1247 hour = 36448.92 sec
$k_{rac} = 1/ t_1 = 2.7435\times10^{-5}$
$k_{enant} = 1.371\times10^{-5}$
$\Delta G^{\neq}$ = 24.22 Kcal.mol^{-1}

3dh at 300K

decay constant = t_1 = 3.5675 day = 308232 sec
$k_{rac} = 1/ t_1 = 3.244\times10^{-6}$
$k_{enant} = 1.622\times10^{-6}$
$\Delta G^{\neq}$ = 25.49 Kcal.mol^{-1}

3di at 300K

decay constant = t_1 = 3.23808 hour = 11657.088 sec
$k_{rac} = 1/ t_1 = 8.578\times10^{-5}$
$k_{enant} = 4.289\times10^{-5}$
$\Delta G^{\neq}$ = 23.54 Kcal.mol^{-1}

Section F: Temperature dependent CD spectral analysis of 3db

[a] CD spectra of (*S*)-3db at 353 K with the course of time

To perform the Electronic Circular Dichroism (ECD) analysis, 1.03 mM solution of (*S*)-**3db** was prepared by dissolving 1.0 mg of (*S*)-**3db** in 1.0 mL EtOH. 400 μL of the above solution was taken in a screw cap quartz cuvette (screw cap is necessary to stop the change of concentration of sample due to solvent evaporation) and placed inside the CD spectrophotometer equipped with thermoelectric temperature controller. Sample was placed inside the preheated CD spectrophotometer at 353 K. CD spectra were recorded with respect to variable time regime up to 30 min at fixed temperature of 353 K. The spectral data was obtained is represented below.

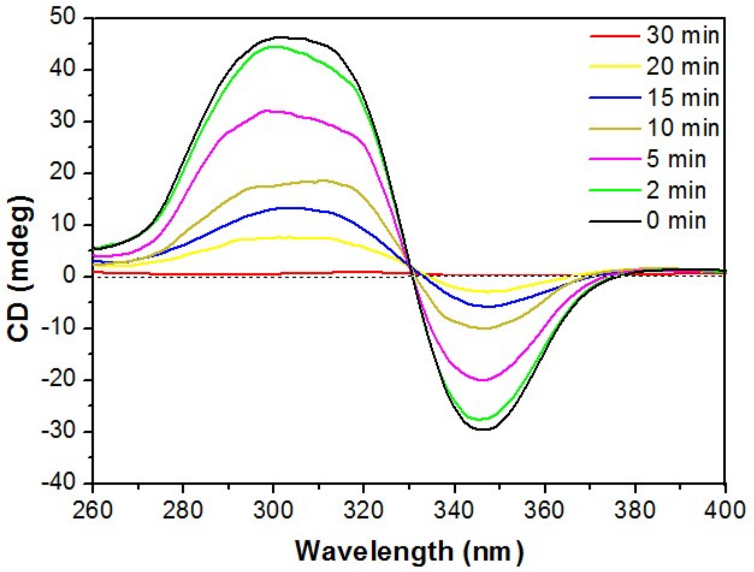

Figure S107: CD spectra of (*S*)-**3db** at 353 K in different time scale

Table S1: CD intensities and calculated enantiomeric excess (*ee*) at 353 K in different time scale.

Time (min)	0	2	5	10	15	20	30
**CD_{max} [(-) mdeg]**	29.58	27.56	20.04	10.03	5.76	2.90	racemic
**ee (%)[a]**	>99.0	92.24	67.07	33.57	19.28	9.70	racemic

[a] Calculated from CD_{max} [(-) mdeg] intensity by unitary method considering 29.58 (-) mdeg value of CD_{max} as >99.0 % ee of 1.03 mM enantiopure *(S)*-**3db**.

[b] CD spectra of (*S*)-3db at 333 K with the course of time

The above indicated CD spectral analysis was also applied to record at 333 K. Sample was placed inside the preheated CD spectrophotometer at 333 K. CD spectra were recorded with respect to variable time regime up to 10 h at fixed temperature of 333 K. The spectral data was obtained is represented below.

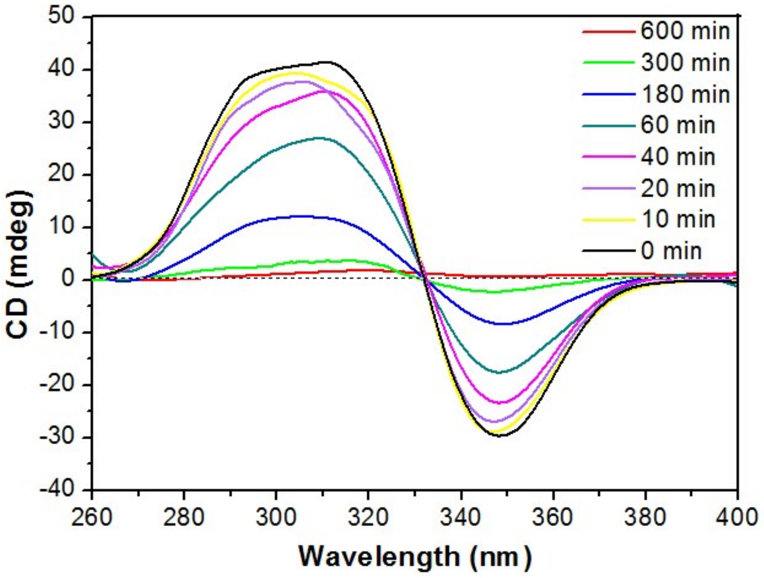

Figure S108: CD spectra of (*S*)-**3db** at 333 K in different time scale

Table S2: CD intensities and calculated enantiomeric excess (*ee*) at 333 K in different time scale.

Time (min)	0	10	20	40	60	180	300	600
CD_{max} (mdeg)	29.65	28.87	26.92	23.37	17.58	8.39	2.24	racemic
ee (%)[a]	>99.0	96.39	89.88	78.03	58.69	28.01	7.48	racemic

[a] Calculated from CD_{max} [(-) mdeg] intensity by unitary method considering 29.65 (-) mdeg value of CD_{max} as >99.0 % ee of 1.03 mM enantiopure *(S)*-**3db**.

Section G: Verification of kinetic and thermodynamical parameters for 3db by ECD analysis

In order to verify the racemization energy and other kinetic and thermodynamical parameters obtained from HPLC analysis, time dependent CD spectral analysis was recorded on enantiopure (*S*)-**3db** following the experimental protocol mentioned in **section D.** The decrease of negative CD intensity [(-) mdeg] with time at 353 K and 333 K incubation temperature were plotted as a function of variable time regime, as shown in figure S108. From the exponential decay curve (shown in figure S109), activation barrier to racemization ($\Delta G^{\neq}$) and other parameters were determined from equations 1-4 described in **section E**. The results obtained from ECD are in well agreement with the HPLC results performed at different temperatures.

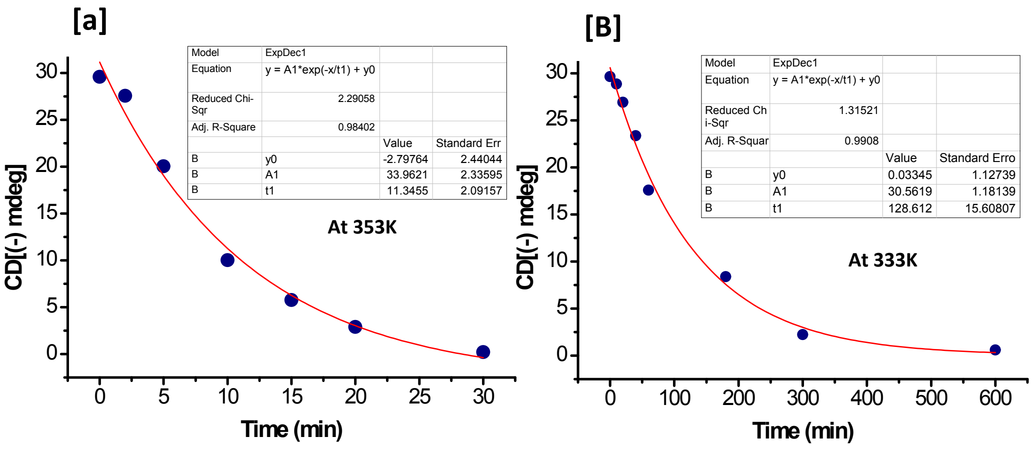

Figure S109: Plot of CD [(-) mdeg] of (*S*)-**3db** as a function of time (min) at (a) 353 K and (b) 333 K

3db at 353K

decay constant = t_1 = 11.34 min = 680.4 sec
$k_{rac} = 1/\ t_1 = 1.47\times10^{-3}$
$k_{enant} = 7.35\times10^{-4}$
$\Delta G^{\neq} = 25.82\ \text{Kcal.mol}^{-1}$

3db at 333K

decay constant = t_1 = 128.61 min = 7716.6 sec
$k_{rac} = 1/\ t_1 = 1.29 \times 10^{-4}$
$k_{enant} = 6.45\times10^{-5}$
$\Delta G^{\neq} = 25.93\ \text{Kcal.mol}^{-1}$

Table S3: Different kinetic and thermodynamic parameters of 3db, 3dg, 3dh and 3di as a function of temperature.

Molecule	Mode of analysis		Physical Parameters				
		Temp (T, K)	Decay Constant (t_1)	Rate Constant (k_{rac}, sec^{-1})	Enantimerisation Constant ($2K_{enant}$= k_{rac})	Half Life ($t_{1/2}$)	Activation Barrier of Racemization ($\Delta G^{\neq}$, kcal.mol^{-1})
**3db**	HPLC	353	10.61 min	1.62×10^{-3}	8.135×10^{-4}	7.13 min	25.75
		333	115.47 min	1.44×10^{-4}	7.21×10^{-5}	80.20 min	25.86
		300	13.43 days	8.62×10^{-7}	4.305×10^{-7}	9.30 days	26.29
	CD	353	11.34 min	1.47×10^{-3}	7.35×10^{-4}	7.85 min	25.82
		333	128.61 min	1.29×10^{-4}	6.45×10^{-5}	78.41 min	25.93
**3dg**	HPLC	300	10.12 h	2.74×10^{-5}	1.371×10^{-5}	7.02 h	24.22
**3dh**	HPLC	300	3.56 days	3.24×10^{-6}	1.62×10^{-6}	2.47 days	25.49
**3di**	HPLC	300	3.23 h	8.57×10^{-5}	4.289×10^{-5}	2.24 h	23.54

Section H: Determination of activation enthalpy ($\Delta H^{\neq}$) and activation entropy ($\Delta S^{\neq}$) of racemization:

The activation enthalpy (ΔH≠) and activation entropy (ΔS≠) of the isomerization of atropisomer **3db** were further determined employing the Eyring equation:

Eqn. 3: $$\ln\frac{k}{T} = -\frac{\Delta H^{\neq}}{R}\frac{1}{T} + \ln\frac{k_B}{h} + \frac{\Delta S^{\neq}}{R}$$

The values for ΔH≠ and ΔS≠ were determined from kinetic data obtained from a $\ln\frac{k_{enant}}{T}$ vs. $\frac{1}{T}$ plot considering temperature (T) as 353, 333 and 300 K (based on HPLC results) as well as 353 K and 333 K (based on ECD results). The equation is a straight line with negative slope, $-\frac{\Delta H^{\neq}}{R}$, and a y-intercept, $\ln\frac{k_B}{h} + \frac{\Delta S^{\neq}}{R}$. The activation enthalpy (ΔH≠) and activation entropy (ΔS≠) of the racemization process were determined as 28.86 kcal mol^{-1} and 8.12 cal mol^{-1} K^{-1} respectively.

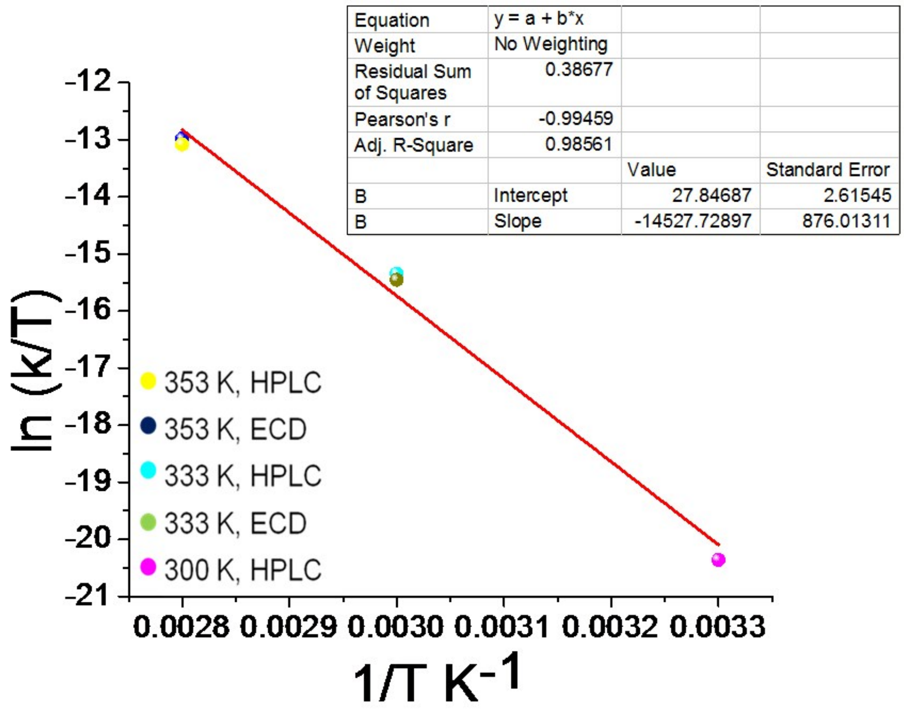

Equation	y = a + b*x		
Weight	No Weighting		
Residual Sum of Squares	0.38677		
Pearson's r	-0.99459		
Adj. R-Square	0.98561		
		Value	Standard Error
B	Intercept	27.84687	2.61545
B	Slope	-14527.72897	876.01311

Figure S110: Eyring plot for the racemization of **3db**

Section I: X-ray crystal structure determination for 3db, 3dg, 3dh and 3di

[a] X-ray crystal structure determination for 3db (CCDC 1852313)

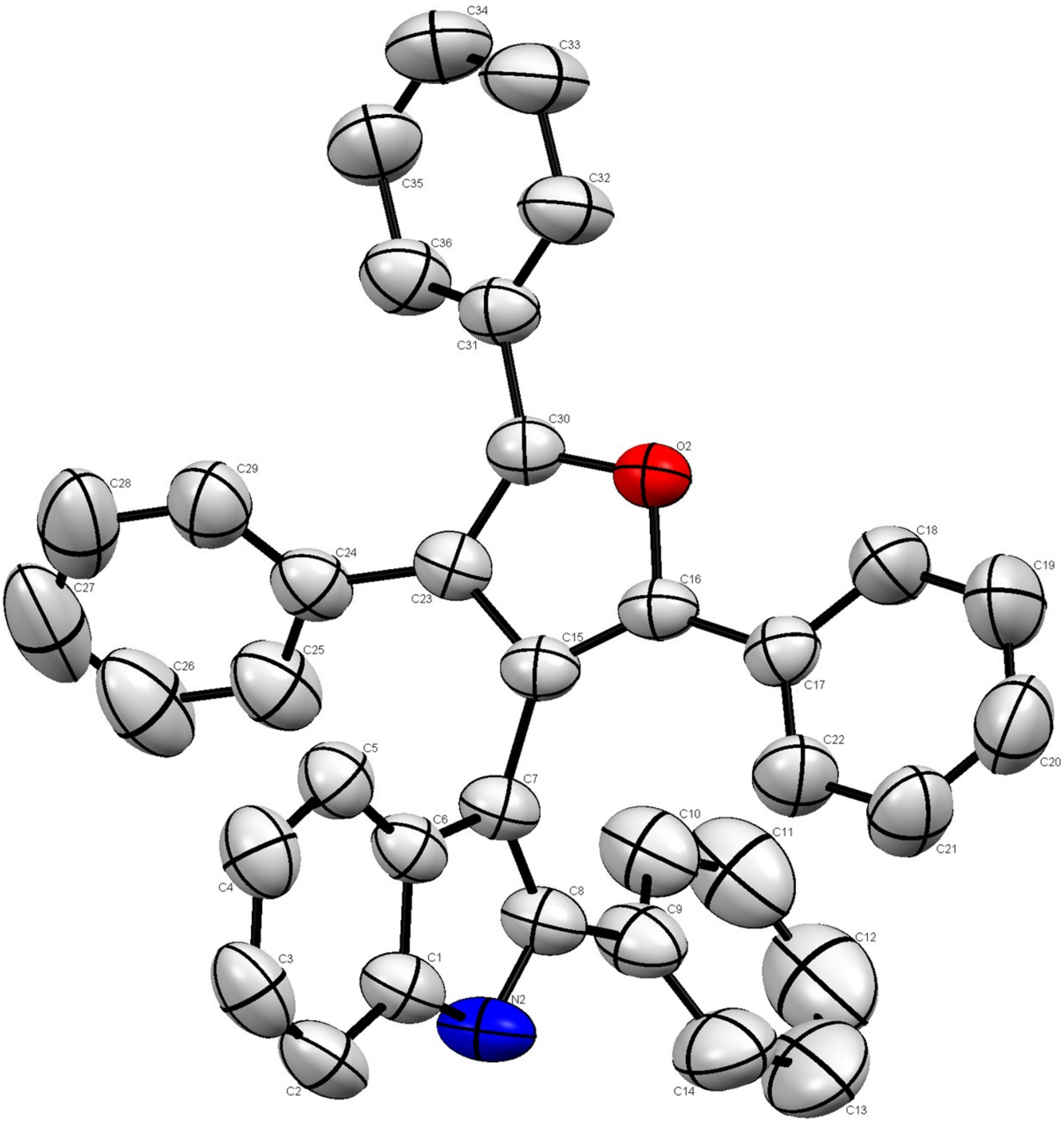

Suitable single crystal with approximate dimensions of 0.15 × 0.12 × 0.04 mm^3 was used for X-ray diffraction analyses by mounting on the tip of a glass fiber in air. Data were collected on a Bruker kappa apex 2 with Mo Kα (λ=0.71073 Å) at 296.15 K. The structure was solved by direct method using program SHELXL-97 and subsequent Fast Fourier Transform technique. Crystallographic data and experimental details for **3db** are summarized in **Table S4**.

Table S4. Crystal data and structure refinement for **3db**

Empirical formula	$2(C_{36}H_{25}NO)$
Formula weight	975.14

Temperature/K	298
Crystal system	monoclinic
Space group	P 2_1/n (14)
a/Å	9.8397(15)
b/Å	38.561(6)
c/Å	14.122(2)
α/°	90
β/°	100.382(8)
γ/°	90
Volume/Å^3	5270.6(14)
Z	4
ρ_{calc}g/cm^3	1.229
μ/mm^{-1}	0.073
F(000)	2048
Crystal size/mm^3	0.04 × 0.12 × 0.15
Radiation	MoKα (λ = 0.71073)
2Θ range for data collection/°	1.1 to 28.5
Index ranges	$-13 \leq h \leq 12$, $-50 \leq k \leq 50$, $-18 \leq l \leq 18$
Reflections collected	76731
Independent reflections	12254 [R_{int} = 0.073]
Observed data [I> 0.0 σ (I)]	5938
Data/restraints/parameters	12254/1/686
Goodness-of-fit on F^2	0.980
Final R indexes	R_1 = 0.0728, wR_2 = 0.2574
Maximum and average shift/error	0.00/ 0.00
Largest diff. peak/hole / e Å^{-3}	-0.26/ 0.26

[b] X-ray crystal structure determination for 3dg (CCDC 1852312)

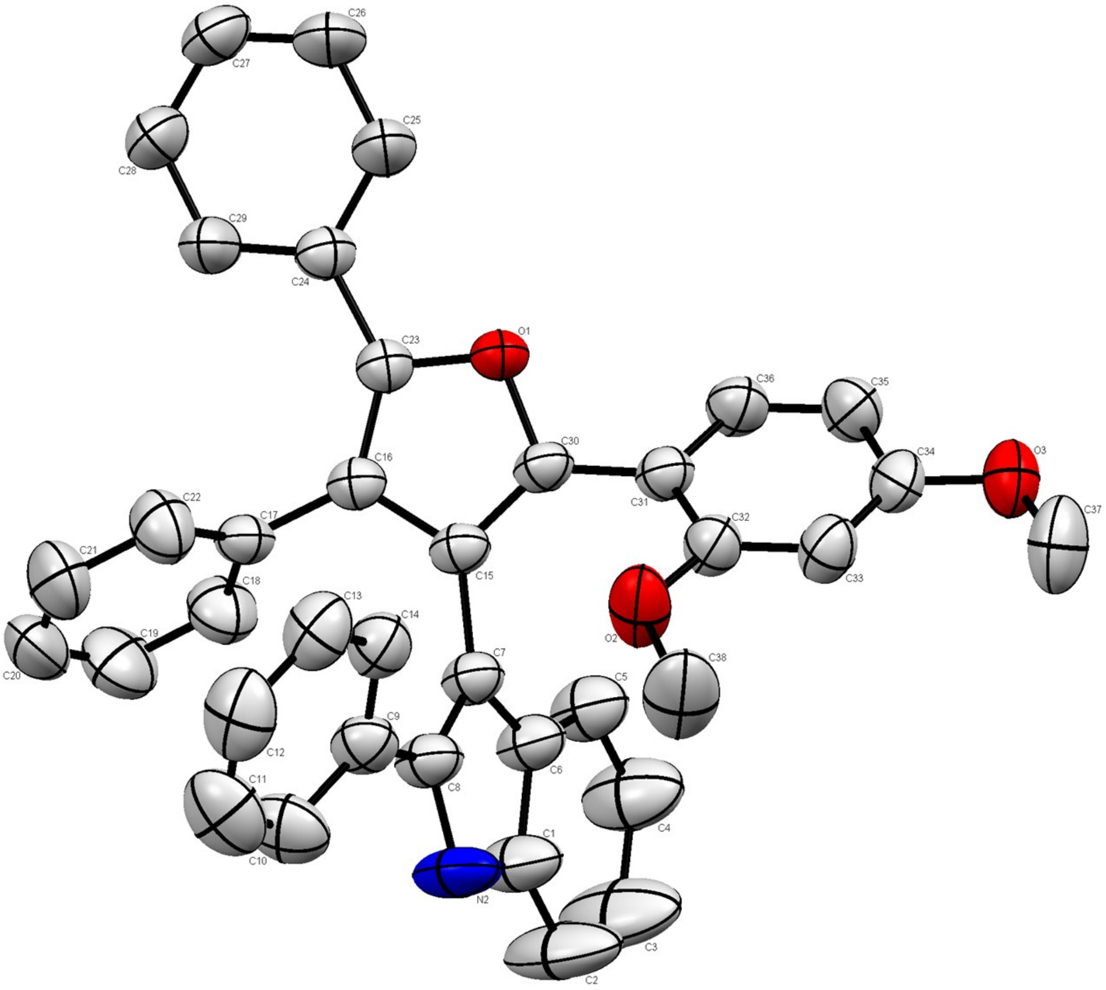

Suitable single crystal with approximate dimensions of 0.12 × 0.10 × 0.05 mm^3 was used for X-ray diffraction analyses by mounting on the tip of a glass fiber in air. Data were collected on a Bruker kappa apex 2 with Mo Kα (λ=0.71073 Å) at 296.15 K. The structure was solved by direct method using program SHELXL-97 and subsequent Fast Fourier Transform technique. Crystallographic data and experimental details for **3dg** are summarized in **Table S5**.

Table S5. Crystal data and structure refinement for **3dg**

Empirical formula	$2(C_{38}H_{29}NO_3)$
Formula weight	1095.24
Temperature/K	298
Crystal system	triclinic
Space group	P-1

a/Å	11.7682(9)
b/Å	13.1204(11)
c/Å	19.9515(16)
α/°	77.621(4)
β/°	77.484(3)
γ/°	85.254(4)
Volume/Å^3	2935.3(4)
Z	2
ρ_{calc}g/cm^3	1.239
μ/mm^{-1}	0.078
F(000)	1152
Crystal size/mm^3	0.05 × 0.10 × 0.12
Radiation	MoKα (λ = 0.71073)
2Θ range for data collection/°	1.1 to 27.6
Index ranges	$-15 \leq h \leq 15$, $-17 \leq k \leq 16$, $-25 \leq l \leq 26$
Reflections collected	60770
Independent reflections	13116 [R_{int} = 0.045]
Observed data [I> 0.0 σ (I)]	7885
Data/restraints/parameters	13116/1/761
Goodness-of-fit on F^2	1.04
Final R indexes	R_1 = 0.0590, wR_2 = 0.1612
Maximum and average shift/error	0.00/ 0.00
Largest diff. peak/hole / e Å^{-3}	0.268/-0.181

[c] X-ray crystal structure determination for 3dh (CCDC 1852314)

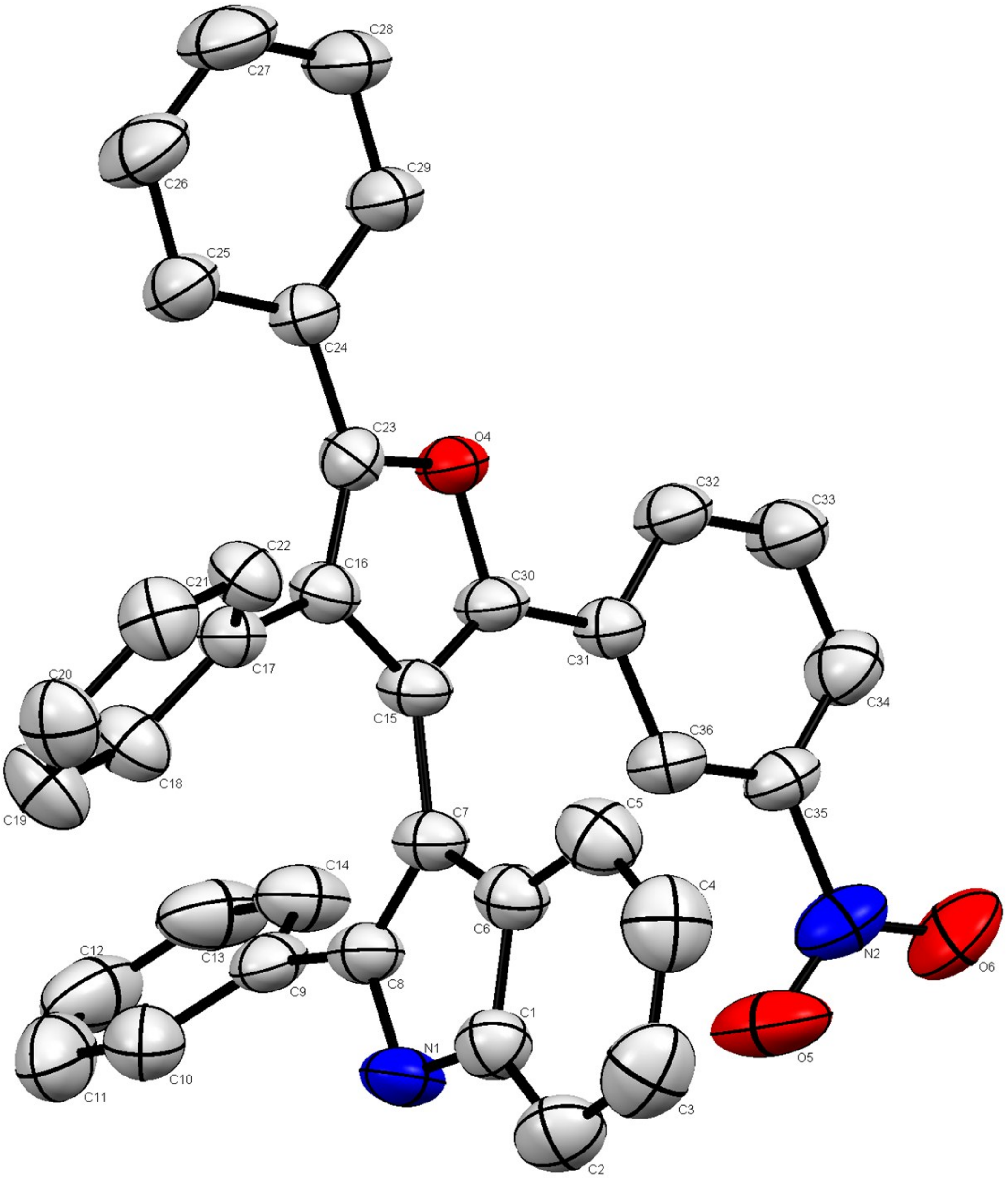

Suitable single crystal with approximate dimensions of 0.2 × 0.05 × 0.05 mm^3 was used for X-ray diffraction analyses by mounting on the tip of a glass fiber in air. Data were collected on a Bruker kappa apex 2 with Mo Kα (λ=0.71073 Å) at 296.15 K. The structure was solved by direct method using program SHELXL-97 and subsequent Fast Fourier Transform technique. Crystallographic data and experimental details for **3dh** are summarized in **Table S6**.

Table S6. Crystal data and structure refinement for **3dh**

Empirical formula	$C_{36}H_{24}N_2O_3$
Formula weight	532.57
Temperature/K	296.15
Crystal system	monoclinic
Space group	P 2_1/n (14)
a/Å	24.204(3)
b/Å	12.6309(13)
c/Å	18.083(2)
α/°	90
β/°	90.124(7)
γ/°	90
Volume/Å^3	5528.3(11)
Z	8
ρ_{calc}g/cm^3	1.280
μ/mm^{-1}	0.082
F(000)	2224
Crystal size/mm^3	0.05 × 0.05 × 0.2
Radiation	MoKα (λ = 0.71073)
2Θ range for data collection/°	0.841 to 24.997
Index ranges	-28≤ h ≤ 28, -14 ≤ k ≤ 15, -21 ≤ l ≤ 21
Reflections collected	119377
Independent reflections	9638 [R_{int} = 0.132]
Observed data [I> 0.0 σ (I)]	5127
Data/restraints/parameters	9638/1/740
Goodness-of-fit on F^2	1.018
Final R indexes	R_1 = 0.0536, wR_2 = 0.1562
Maximum and average shift/error	0.00/ 0.00

Largest diff. peak/hole / e Å^{-3}	0.237/-0.236

[d] X-ray crystal structure determination for 3di (CCDC 1888443)

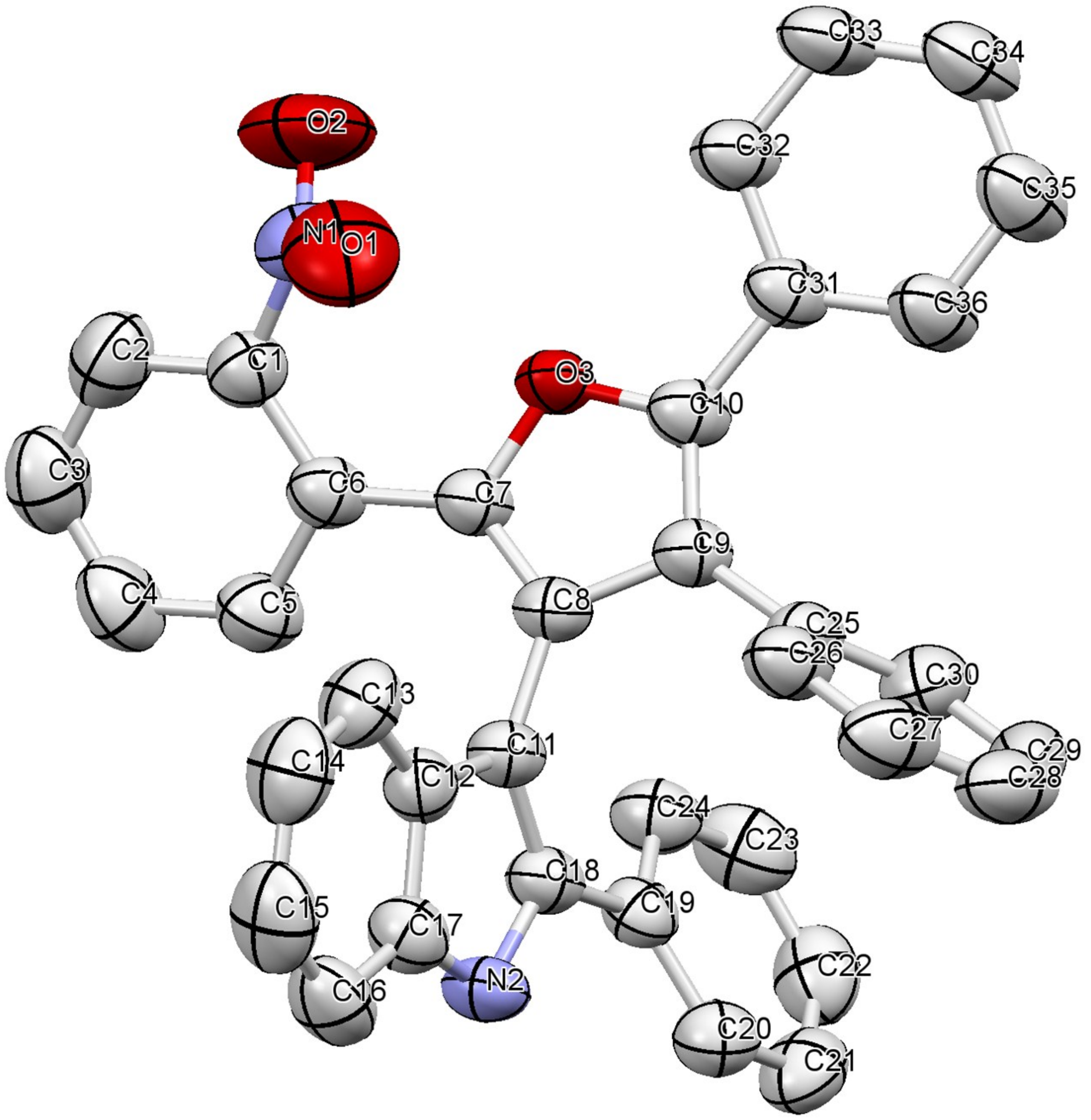

Suitable single crystal with approximate dimensions of 0.8 × 0.7 × 0.12 mm^3 was used for X-ray diffraction analyses by mounting on the tip of a glass fiber in air. Data were collected on a 'Bruker APEX-II CCD' diffractometer with Mo Kα (λ=0.71073 Å) at 296.15 K. Using Olex2 , the structure was solved with the ShelXT structure solution program using Intrinsic Phasing and refined with the ShelXL refinement package using Least Squares minimization. Crystallographic data and experimental details for **3di** are summarized in **Table S7**.

Table S7. Crystal data and structure refinement for **3di**

Empirical formula	C36H24N2O3
Formula weight	532.57
Temperature/K	296.15
Crystal system	triclinic
Space group	P-1
a/Å	10.259(7)
b/Å	11.282(7)
c/Å	13.341(9)
α/°	70.807(8)
β/°	84.165(9)
γ/°	71.005(8)
Volume/Å3	1378.9(16)
Z	2
ρcalcg/cm3	1.283
μ/mm 1	0.082
F(000)	556.0
Crystal size/mm3	0.8 × 0.7 × 0.12
Radiation	MoKα (λ = 0.71073)
2Θ range for data collection/°	4.828 to 54.696
Index ranges	-13 ≤ h ≤ 13, -14 ≤ k ≤ 14, -17 ≤ l ≤ 16
Reflections collected	24460
Independent reflections	6030 [Rint = 0.0517, Rsigma = 0.0526]
Data/restraints/parameters	6030/0/371
Goodness-of-fit on F2	1.092

Final R indexes [I>=2σ (I)]	R1 = 0.0963, wR2 = 0.3352
Final R indexes [all data]	R1 = 0.1389, wR2 = 0.3580
Largest diff. peak/hole / e Å-3	0.34/-0.30

Section J: Additional computational details and results

Methods: All modelling for this work was done with Gaussian09 or Gaussian16 (citations at the end of this section). The CD spectra were optimised and predicted at the B3LYP/6-311+G** level of theory in PCM solvent ethanol, and used the TD=(nstates=18) and IOP(9/40=2) options. All energies and optimizations given below are at the M062X/6-311G** level of theory. Transition states were optimized with the Opt=(TS, NoEigenTest, CalcAll) settings. Transition states and minima were verified with frequency calculations. Free energies are predicted at 298.15 K. All axial torsions were measured from furan C2 to indole C3 (highlighted below). All models below are optimized in vacuo, save for the solvated **3db** models (Table S8) which used default PCM solvent representations.

Table S8. Predicted axial torsions, relative energies, and thermodynamic paramters various models of optimized and transition state structures for **3da-3di**. For structures of **core-1**, **core-2**, and **3da-vatiant**, see below.

molecule	structure	Axial Torsion (degrees)	Relative Energy (kcal/mol)	Relative Free Energy (kcal/mol)	Relative Enthalpy (kcal/mol)
**core-1**	min	-147	0.0	0.0	0.0
	TS2	0	2.1	2.8	1.4
	TS2	-96	1.6	2.2	1.0
	TS1	180	0.7	1.5	0.1
**core-2**	min	-146	0.0	0.0	0.0
	TS1	0	2.2	3.1	1.6
	TS2	-98	1.6	2.2	0.8
	TS3	180	0.8	1.5	0.0
**3da variant**	min	-46	0.0	0.0	0.0
	TS-0	15	11.9	13.4	11.3
	TS-180	180	12.6	12.4	12.1

**3bb**	min	-130	0.0	0.0	0.0
	TS-0	6	23.8	25.1	23.2
	TS-180	176	22.9	24.4	22.3
**3db**	min	-132	0.0	0.0	0.0
	TS-0	8	27.2	28.2	26.3
	TS-180	-173	25.5	26.0	24.3
**3db-water**	min	-131	0.0	0.0	0.0
	TS-0	8	26.6	28.2	25.8
	TS-180	-174	25.1	26.7	24.2
**3db-ethanol**	min	-131	0.0	0.0	0.0
	TS-0	8	26.6	28.2	25.9
	TS-180	-174	25.1	26.7	24.3
**3dc**	min	-48	0.0	0.0	0.0
	TS-0	-8	22.6	23.1	21.7
	TS-180	-179	21.7	22.4	20.9
**3dd**	min	-131	0.0	0.0	0.0
	TS-0	8	26.1	28.3	25.6
	TS-180	175	25.4	26.4	24.4
**3de**	min	-132	0.0	0.0	0.0
	TS-0	7	26.3	26.6	25.0
	TS-180	-175	25.5	25.6	24.1
**3dg**	min-1	-132	0.0	0.0	0.0
	min-2	-133	3.6	3.4	4.0
	TS-0	1	23.7	25.2	22.7
	TS-180	-173	26.9	27.6	26.2
**3dh**	min	-130	0.0	0.0	0.0
	TS-0	3	21.4	25.3	20.8
	TS-180	-174	25.5	27.7	24.6
**3di**	min-1	-132	0.0	0.0	0.0
	min-2	134	2.2	0.7	2.2
	TS-0	8	22.5	23.4	21.7
	TS-180	-175	25.2	25.4	24.3

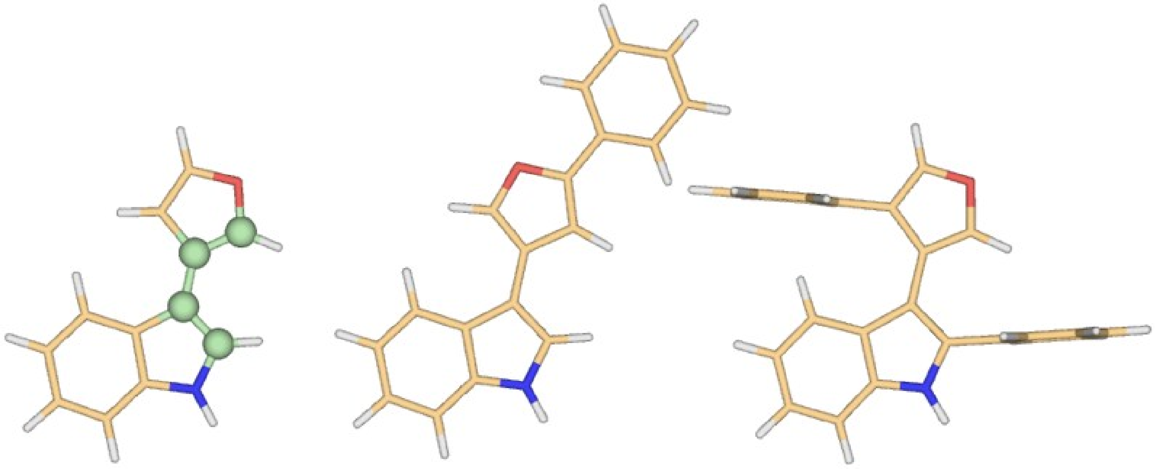

Figure S111: Left: structures of **core-1**, highlighting the carbons used to measure the axial torsions in the above table; Middle: **core-2**; Right: **3da variant** missing the furan C5 phenyl group.

Comparative study of isomerization energy barrier of 2-methyl indole substitution (3bb) with 2-phenyl indole substitution (3db) through DFT modelling

The predicted isomerization energy barrier of Molecule **3bb**, which has a methyl group at the 2-indole position, is 22.9 kcal/mol (24.4 kcal/mol $\Delta G^{\ddagger}$). This is 2.6 kcal/mol lower than the barrier of **3db**, which has a phenyl at the same position (1.6 kcal/mol lower in $\Delta G^{\ddagger}$). That the methyl substituted species presents a lower barrier might seem counterintuitive, as a methyl is sterically bulkier than a phenyl (when measured normal to the plane of the aromatic ring). However, the furan bond angles at the 2 and 4 positions orient the phenyl groups towards the indole. The predicted transition states show the phenyl groups acting as rigid leaver arms, which must pass the indole to allow axial inversion. In the more favourable of the two **3bb** transition states, the 4-furan phenyl slides over the 2-indole methyl group causing the angle between the 4-furan carbon, 1-phenyl carbon, and 4-phenyl carbon to bend out of plane to 175.7° (Figure S112). By comparison, in the corresponding **3db** transition state, nonplanarity of the matching atoms increases to 170.5° (Figure S112 right).

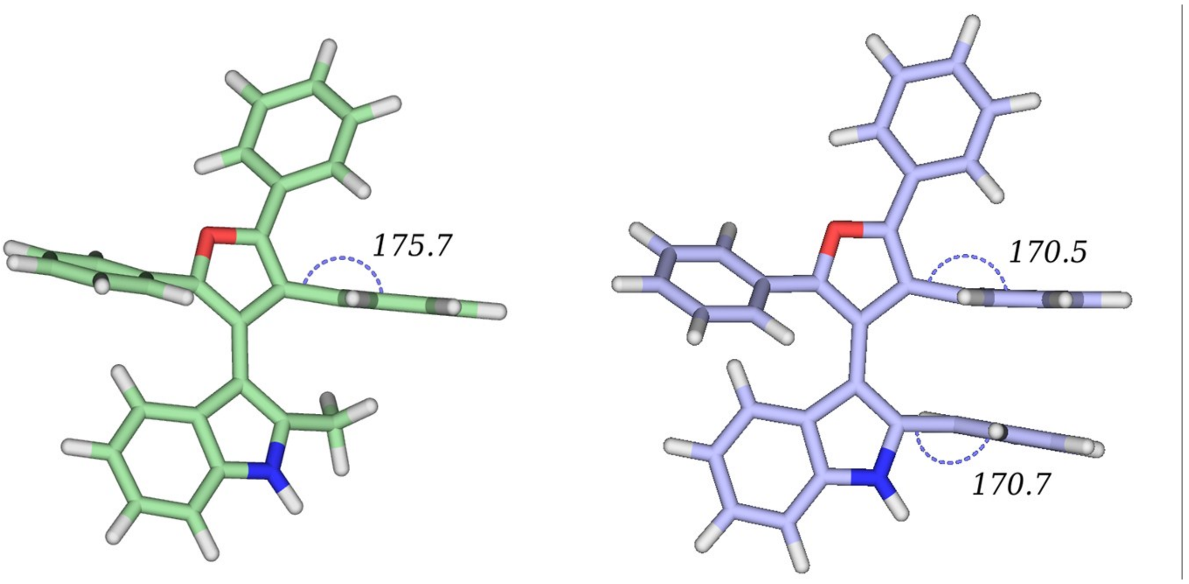

Figure S112. Transition states of **3bb** (left, 22.9 kcal/mol) and **3db** (right, 25.5 kcal/mol), highlighting the nonplanarity of relevant phenyl groups.

<u>Additional analysis regarding the barrier of rotation of 3dg</u>

Molecule **3dg** interconverts relatively rapidly compared to similar species. However, its overall barrier height is predicted to be in the same range as the more stable molecules (predicted $\Delta G^{\ddagger}$ of 27.6 kcal/mol vs. 26.0 for **3db** $\Delta G^{\ddagger}$). A notable observation from **3dg** modelling is that the 2-methoxy phenyl substitution leads to a second local minimum pose which is ~4 kcal/mol above the predicted global minimum (Figure S113, bottom). In the higher energy structure, the methoxy oxygen sits proximal to the furan ring oxygen. The higher energy structure is predicted to be metastable—a potential energy surface for rotation around the furan-dimethoxyphenyl bond shows a barrier in the range of 2 kcal/mol (Figure S113, top).

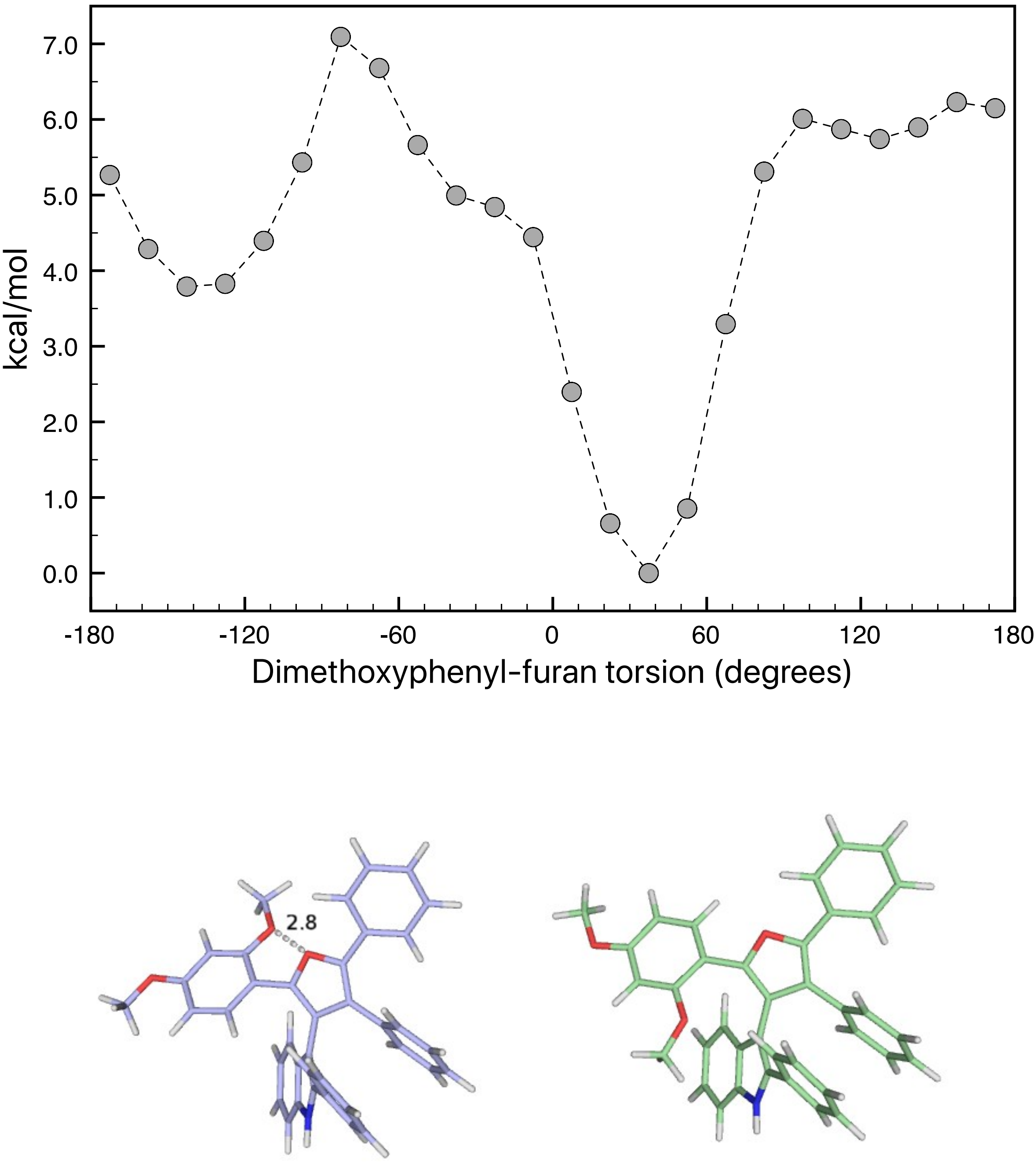

Figure S113. Top: Potential energy surface of rotation about **3dg** furan-dimethoxyphenyl bond calculated at the M062X/6-311G** level of theory. Bottom: Structures of the higher (left) and lower (right) energy local minima.

Predicted low barrier of rotation for 3di

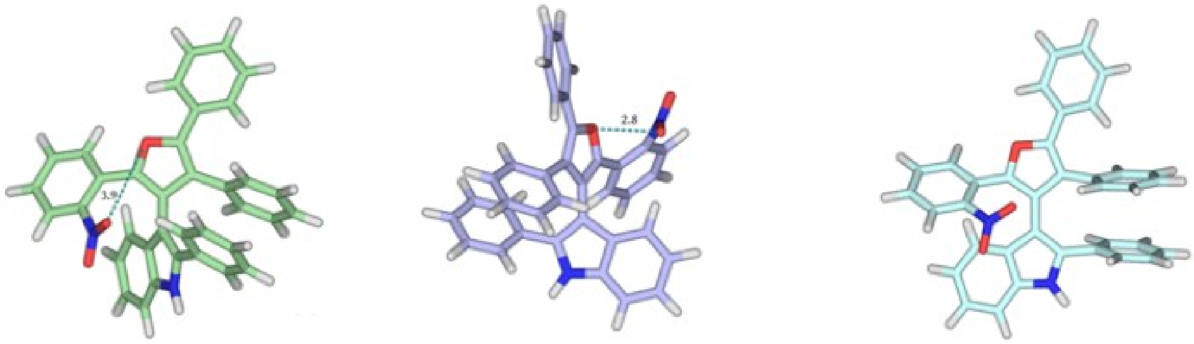

Figure S114. Local minima (left, middle) and the lowest energy transition state (right) of molecule **3di**. Also shown are the closest furan oxygen to nitro oxygen distance (for the minima) and the relative ΔG of the poses left to right are: 0.0, 0.7, and 23.4 kcal/mol, respectively. The nitro orientation in the middle structure resembles that of the crystal structure.

Tables of selected atom coordinates and absolute energies:

Optimized coordinates of 3db min in vacuo (Ground State):

Table S9. Cartesian coordinates (in Å) of the M062X/6-311G** optimized structure of the electronic ground state of **3db**. (Total energy: M062X/6-311G**: -1516.64146655 Hartrees, 0 imaginary frequencies)

Atom	x	y	z
N	-2.27295	-2.75756	0.06073
C	-1.83811	2.50829	0.64232
C	-2.93182	4.64912	0.90832
C	-4.04842	4.02039	1.44965
C	-4.05589	2.63656	1.5931
C	-2.96017	1.88207	1.19717
C	-0.67291	1.73506	0.21858
C	2.79404	2.14006	-0.49771
C	1.46529	1.56279	-0.2933
C	0.94594	0.30364	-0.40401
C	-0.4576	0.41639	-0.08059
C	3.14608	3.3181	0.16939
C	4.40169	3.8808	-0.01515
C	5.32114	3.27792	-0.86672

C	4.97279	2.11166	-1.54121
C	3.71759	1.54585	-1.36501
C	-1.41838	-0.68814	-0.11533
C	-2.61991	-0.76	-0.90535
C	-3.13688	-2.06556	-0.75856
C	-1.25296	-1.92289	0.46925
C	-0.18877	-2.39837	1.36085
C	0.28245	-1.57562	2.38809
C	1.30775	-2.01078	3.21624
C	1.87178	-3.27011	3.03049
C	1.40782	-4.09194	2.00943
C	0.38436	-3.65892	1.17597
C	1.65336	-0.94699	-0.74133
C	2.76981	-1.34382	-0.00129
C	3.42179	-2.53427	-0.29252
C	2.96158	-3.34813	-1.32312
C	1.84544	-2.96436	-2.05985
C	-3.29652	0.16187	-1.71526
C	-4.46507	-0.23551	-2.3368
C	-4.97182	-1.53755	-2.16876
C	-4.31898	-2.46908	-1.38164
C	1.19228	-1.77095	-1.77048
C	-1.83388	3.90166	0.50642
O	0.48859	2.42798	0.08342
H	5.67854	1.64517	-2.2182
H	-2.44538	-3.66555	0.4607
H	-2.9101	1.16812	-1.83454
H	-5.89242	-1.81606	-2.6672
H	-2.91682	5.72658	0.79443
H	-4.91833	2.14002	2.02121
H	-2.96816	0.80668	1.32407
H	2.42899	3.78217	0.8353
H	4.66345	4.79127	0.51067
H	6.30136	3.71715	-1.00908
H	3.4454	0.6496	-1.90863
H	-0.1653	-0.59854	2.53157
H	1.66464	-1.36771	4.01192

H	2.6726	-3.60729	3.67777
H	1.85652	-5.0653	1.84864
H	0.05657	-4.27871	0.3483
H	3.11415	-0.71402	0.81163
H	4.28325	-2.83205	0.29391
H	3.46902	-4.27856	-1.54958
H	1.48315	-3.59357	-2.86453
H	-5.00603	0.46478	-2.96124
H	-4.70892	-3.47247	-1.25698
H	0.31833	-1.4713	-2.33916
H	-0.96574	4.38804	0.07956
H	-4.90633	4.6051	1.75941

Optimized coordinates of 3db T.S.-I in vacuo (~0 degree axial torsoin):

Table S10. Cartesian coordinates (in Å) of the M062X/6-311G**: optimized structure of the electronic transition state-I of **3db**. (Total energy M062X/6-311G**: -1516.59816795 Hartrees, 1 imaginary frequency)

Atom	x	y	z
N	-2.37663	2.80878	0.27814
C	-1.72726	-1.93864	0.19188
C	-3.03762	-3.02736	1.90852
C	-3.87800	-3.55335	0.93386
C	-3.64455	-3.27216	-0.40829
C	-2.56851	-2.47454	-0.77958
C	-0.46214	-1.23680	-0.13685
C	2.92639	-2.32161	-0.22168
C	1.72303	-1.48154	-0.18825
C	1.49086	-0.13952	-0.10576
C	0.04064	0.04685	-0.11502
C	4.10456	-1.96138	0.44151
C	5.21358	-2.79570	0.39724
C	5.16434	-3.99993	-0.29730
C	3.99038	-4.37194	-0.94338
C	2.87880	-3.54144	-0.90530

C	-0.70573	1.32881	-0.07384
C	-0.18094	2.69799	-0.15842
C	-1.25788	3.57421	0.10998
C	-2.07369	1.47790	0.10718
C	-3.27670	0.63327	-0.11125
C	-3.71846	0.48953	-1.42889
C	-4.93407	-0.12590	-1.70088
C	-5.71847	-0.60141	-0.65624
C	-5.28286	-0.46197	0.65746
C	-4.07199	0.16312	0.93162
C	2.58898	0.82269	0.11710
C	2.65365	1.53966	1.31241
C	3.69598	2.42856	1.54330
C	4.69126	2.60079	0.58635
C	4.64514	1.87190	-0.59884
C	1.01997	3.31180	-0.56940
C	1.12901	4.68936	-0.58517
C	0.05999	5.52022	-0.21415
C	-1.15675	4.96706	0.12275
C	3.60095	0.98515	-0.83112
C	-1.96465	-2.22668	1.53772
O	0.55047	-2.14295	-0.22051
H	3.93916	-5.31171	-1.48029
H	-3.32186	3.15331	0.32241
H	1.85840	2.73428	-0.91704
H	0.18137	6.59658	-0.22558
H	-3.21047	-3.24909	2.95520
H	-4.30185	-3.67593	-1.16943
H	-2.37716	-2.26173	-1.82481
H	4.14846	-1.03804	1.00441
H	6.11819	-2.50644	0.91876
H	6.03274	-4.64717	-0.32736
H	1.96156	-3.83120	-1.40298
H	-3.10020	0.87259	-2.23334
H	-5.26985	-0.22947	-2.72599
H	-6.66661	-1.08377	-0.86324
H	-5.88668	-0.84269	1.47271

H	-3.73025	0.27538	1.95400
H	1.86716	1.41174	2.04807
H	3.72762	2.99327	2.46737
H	5.50084	3.29860	0.76394
H	5.42156	1.99734	-1.34442
H	2.06355	5.13310	-0.90693
H	-2.01673	5.58123	0.36382
H	3.55593	0.41672	-1.75342
H	-1.29145	-1.82550	2.28734
H	-4.71431	-4.18147	1.21821

Optimized coordinates of 3db TS-II in vacuo (~180 degrees axial torsoin):

Table S11. Cartesian coordinates (in Å) of the M062X/6-311G**: optimized structure of the electronic transition state-II of **3db**. (Total energy M062X/6-311G**: -1516.60077176 Hartrees, 1 imaginary frequency)

Atom	x	y	z
N	-1.78017	-3.19480	0.18459
C	-2.41734	2.12824	0.23313
C	-4.12677	3.76054	-0.26543
C	-4.87577	3.31088	0.81805
C	-4.38602	2.28529	1.62042
C	-3.15968	1.69948	1.33517
C	-1.10539	1.53794	-0.06036
C	2.21959	2.77282	-0.28243
C	1.06347	1.87214	-0.17335
C	0.89129	0.52215	-0.03685
C	-0.54773	0.27691	-0.03796
C	2.02312	4.03284	-0.86435
C	3.07441	4.92846	-0.99202
C	4.34437	4.58768	-0.53916
C	4.54655	3.34515	0.04982
C	3.49901	2.44217	0.18224
C	-1.27741	-1.00687	-0.05865
C	-2.72103	-1.20395	-0.23041
C	-2.98224	-2.57676	-0.02477
C	-0.75194	-2.28260	0.09003

C	0.56834	-2.92563	-0.13922
C	1.30950	-3.51688	0.88131
C	2.43265	-4.27820	0.57685
C	2.81000	-4.47262	-0.74752
C	2.06971	-3.89150	-1.77081
C	0.95328	-3.12236	-1.46813
C	2.04226	-0.33906	0.33366
C	2.29751	-0.53129	1.69397
C	3.47307	-1.14153	2.11430
C	4.40662	-1.56895	1.17586
C	4.14732	-1.40319	-0.18110
C	-3.80335	-0.42385	-0.68272
C	-5.06181	-0.98630	-0.79304
C	-5.29727	-2.33430	-0.48016
C	-4.25113	-3.15258	-0.10728
C	2.97293	-0.78901	-0.60007
C	-2.90023	3.17754	-0.55291
O	-0.14068	2.48261	-0.20917
H	5.52825	3.07240	0.41851
H	-1.62603	-4.18990	0.17028
H	-3.67178	0.60196	-0.98530
H	-6.29718	-2.74196	-0.56680
H	-4.50011	4.56473	-0.88818
H	-4.96075	1.94047	2.47156
H	-2.77603	0.89521	1.95222
H	1.03508	4.30331	-1.21386
H	2.90069	5.89614	-1.44791
H	5.16609	5.28658	-0.63906
H	3.68316	1.49190	0.66286
H	1.01136	-3.36574	1.91242
H	3.01479	-4.71892	1.37762
H	3.68208	-5.07235	-0.98081
H	2.35769	-4.04051	-2.80485
H	0.36171	-2.67142	-2.25734
H	1.57934	-0.16251	2.41853
H	3.66784	-1.26797	3.17305
H	5.33017	-2.03482	1.49947

H	4.86518	-1.74761	-0.91653
H	-5.88106	-0.37016	-1.14313
H	-4.39489	-4.20920	0.08611
H	2.78025	-0.63456	-1.65550
H	-2.31381	3.51708	-1.39863
H	-5.83460	3.76395	1.03996

Gaussian citations:

Gaussian 09, Revision D.01,
M. J. Frisch, G. W. Trucks, H. B. Schlegel, G. E. Scuseria,
M. A. Robb, J. R. Cheeseman, G. Scalmani, V. Barone, B. Mennucci,
G. A. Petersson, H. Nakatsuji, M. Caricato, X. Li, H. P. Hratchian,
A. F. Izmaylov, J. Bloino, G. Zheng, J. L. Sonnenberg, M. Hada,
M. Ehara, K. Toyota, R. Fukuda, J. Hasegawa, M. Ishida, T. Nakajima,
Y. Honda, O. Kitao, H. Nakai, T. Vreven, J. A. Montgomery, Jr.,
J. E. Peralta, F. Ogliaro, M. Bearpark, J. J. Heyd, E. Brothers,
K. N. Kudin, V. N. Staroverov, T. Keith, R. Kobayashi, J. Normand,
K. Raghavachari, A. Rendell, J. C. Burant, S. S. Iyengar, J. Tomasi,
M. Cossi, N. Rega, J. M. Millam, M. Klene, J. E. Knox, J. B. Cross,
V. Bakken, C. Adamo, J. Jaramillo, R. Gomperts, R. E. Stratmann,
O. Yazyev, A. J. Austin, R. Cammi, C. Pomelli, J. W. Ochterski,
R. L. Martin, K. Morokuma, V. G. Zakrzewski, G. A. Voth,
P. Salvador, J. J. Dannenberg, S. Dapprich, A. D. Daniels,
O. Farkas, J. B. Foresman, J. V. Ortiz, J. Cioslowski,
and D. J. Fox, Gaussian, Inc., Wallingford CT, 2013.

Gaussian 16, Revision B.01,
M. J. Frisch, G. W. Trucks, H. B. Schlegel, G. E. Scuseria,
M. A. Robb, J. R. Cheeseman, G. Scalmani, V. Barone,
G. A. Petersson, H. Nakatsuji, X. Li, M. Caricato, A. V. Marenich,
J. Bloino, B. G. Janesko, R. Gomperts, B. Mennucci, H. P. Hratchian,
J. V. Ortiz, A. F. Izmaylov, J. L. Sonnenberg, D. Williams-Young,
F. Ding, F. Lipparini, F. Egidi, J. Goings, B. Peng, A. Petrone,
T. Henderson, D. Ranasinghe, V. G. Zakrzewski, J. Gao, N. Rega,
G. Zheng, W. Liang, M. Hada, M. Ehara, K. Toyota, R. Fukuda,
J. Hasegawa, M. Ishida, T. Nakajima, Y. Honda, O. Kitao, H. Nakai,
T. Vreven, K. Throssell, J. A. Montgomery, Jr., J. E. Peralta,
F. Ogliaro, M. J. Bearpark, J. J. Heyd, E. N. Brothers, K. N. Kudin,
V. N. Staroverov, T. A. Keith, R. Kobayashi, J. Normand,
K. Raghavachari, A. P. Rendell, J. C. Burant, S. S. Iyengar,
J. Tomasi, M. Cossi, J. M. Millam, M. Klene, C. Adamo, R. Cammi,
J. W. Ochterski, R. L. Martin, K. Morokuma, O. Farkas,
J. B. Foresman, and D. J. Fox, Gaussian, Inc., Wallingford CT, 2016.

Section K: References

1. R.N. Gupta, V.A. Vaidya, N.M. Bhatia, C.R. Kokare, I. Singhvi and M.S. Bhatia. *Asian Journal of Chemistry.* 2006, **18**, 937.

2. Y. Yang, M. Gao, L. M. Wu, C. Deng, D. X. Zhang, Y. Gao, Y. P. Zhu, A. X. Wu. *Tetrahedron,* 2011, **67***,* 5142.

3. H. S. P. Rao, B. K. Gorityala, and K. Vasantham, *Ind. Journal of Heterocyclic Chem,* **2006**, *15,* 331

4. D. E. Smith, I. Marquez, M. E. Lokensgard, A. L. Rheingold, D. A. Hecht, and J. L. Gustafson , *Angew.Chem.Int.Ed.* 2015, **54**, 11754.

5. E. Kumarasamy, R. Raghunathan, M. P. Sibi, and J. Sivaguru, *Chem. Rev.* 2015, **115**, 20, 11239.

6. S. Chatterjee, G. L. Butterfoss, M. Mandal, B. Paul, S. Gupta, R. Bonneau and P. Jaisankar, *RSC Adv.,* 2016, **6**, 71245

7. D. V. Kosynkin, A. L. Higginbotham, A. Sinitskii, J. R. Lomeda, A. Dimiev, B. K. Price and J. M. Tour, *Nature*, 2009, **458**, 872.

8. E. Marsili, D. B. Baron, I. D. Shikhare, D. Coursolle, J. A. Gralnick and D. R. Bond, *Proc. Natl. Acad. Sci. U.S.A,* 2008, **105**, 3968.

9. W. Verstraete and K. Rabaey, *Environ. Sci. Technol.,* 2006, **40**, 5181.

10. A. J. Bard and L. R. Faulkner, *Electrochemical Methods: Fundamentals and Applications , 2 edn., John Wiley and Sons,* 2001.
